# Society of Nematologists 1961

**DOI:** 10.2478/jofnem-2023-0047

**Published:** 2023-10-16

## Evaluation of nematicides for managing plant-parasitic nematodes in Louisiana sugarcane

Aguilar, Iris M. and T. Watson

Dept. of Plant Pathology & Crop Physiology, Louisiana State University Agricultural Center, Baton Rouge, LA 70803

### Abstract

*Tylenchorhynchus* (stunt), *Pratylenchus* (lesion), *Mesocriconema* (ring), and *Helicotylenchus* (spiral) are plant-parasitic nematode (PPN) genera commonly found in sugarcane fields in Louisiana. PPN have represented an underestimated threat to this economically important crop for many years. In this study, greenhouse pot experiments were conducted to evaluate the efficacy of two nematicides to manage PPN on sugarcane. Soil was collected from seven commercial sugarcane fields with moderate nematode infestations based on prior survey work. Collected field soil from each site was subjected to the following treatments: (1) no treatment, (2) steam-sterilization, (3) Mocap 15G at an equivalent rate of 26 lb/A (active ingredient: ethoprop), and (4) Nimitz at an equivalent rate of 7 pints/A (active ingredient: fluensulfone). Single sugarcane stalk cuttings (HoCP 96-540) were planted in pots filled with soil. Both nematicides were applied at planting. Mocap 15G was applied by mixing the granules into the soil and Nimitz was applied to the soil as a 25-mL drench application. After three months in a greenhouse, PPN were extracted from the soil and roots of each plant to quantify nematode population densities. Plant growth was also evaluated by measuring shoot height, number of tillers, root and shoot weight, and total biomass. The entire experiment was performed twice. Lesion and stunt nematodes were the most abundant PPN in all seven of the sugarcane fields. Untreated soil had the highest density of PPN across all seven sites, whereas low or no nematodes were detected in the steam-sterilized soil. There was a significant main-factor effect of experimental replicate on root parasitism by lesion nematode, which were more abundant in experiment 1 when temperatures were warmer, suggesting that the cooler temperatures in experiment 2 may have negatively affected the rate of nematode reproduction and/or parasitism. Nimitz showed positive results by decreasing lesion nematode population in soil by 47% in the experiment 1 and 24% in the experiment 2, while Mocap 15G reduced lesion nematode by 61% and 33% in soil for experiment 1 and 2 respectively. For stunt nematode, Mocap presented better results in decreasing the number of nematodes by 55% in experiment 1 and 35% in experiment 2. However, in experiment 2, less biomass production and plant height were observed on sugarcane plants treated with Mocap 15G. These data suggest that Mocap 15G may have a negative effect on sugarcane growth. No differences were observed in tiller production between Nimitz and steam-sterilized soil. In conclusion, data suggest that PPNs may influence sugarcane plant growth, and the nematicides Mocap 15G and Nimitz showed potential to manage PPNs on sugarcane.

## Utility of soil samples taken in early January for detecting and predicting damage from plant-parasitc nematodes in South Carolina

Ahmed, Saleh M., T. Fickling, W. Bonnette and J. D. Mueller

Clemson University, Edito Research and Education Center. 64 Research Rd, Blackville, SC 29817

### Abstract

A total of 109 soil samples were collected between January 13 and January 20, 2023 from four farms in Barnwell County, South Carolina. All farms were within 12 miles of each other and have been clustered for analysis. In the 2022 growing season 9 fields were planted to corn, 52 to cotton, 17 to soybean, 22 to peanut and nine to sunflowers or left fallow. Most fields were 10 to 20 ha. Samples were taken using a 2.54-cm diameter sampling tube inserted 25-cm into the soil using a hydraulic piston mounted on a small all-terrain vehicle. At least 12 cores were collected per field and pooled. Nematodes were extracted using differential sieving and centrifugal flotation. All samples containing suitable amounts of *Meloidogyne* juveniles which were identified to species using molecular methods based on mitochondria DNA *(mtDNA)* using two pairs of primers, TRNAH/MRH106 and MORF/MTHIS. All root–knot nematodes were determined to be *Meloidogyne incognita*. All other nematodes were identified to genera using morphological traits. *Meloidogyne incognita* was the most common nematode detected in 39% of all fields and 27% were over threshold. These levels were similar to those reported from fall samples taken from these crops. Columbia lance nematode, *Hoplolaimus columbus*, was detected in 17% of all fields sampled and 5% were over threshold. Stubby root (*Paratrichodorus* spp.), lesion (*Pratylenchus* spp.), and sting (*Belonolaimus* spp.) nematodes were all detected in less than 5% of the samples. Growers are normally encouraged to use post-harvest samples taken in October or November of the previous year to determine levels of plant-parasitic nematodes relative to thresholds established for those sampling times. Nematode densities are normally considered to be too low in January to be used for predictive purposes, especially for root-knot nematodes. However, in 2023, January samples provided growers with useful information on fields that harbor potentially damaging levels of nematodes for row crops in South Carolina.

## Evaluation of winter cover crops for host suitability of *Mesocriconema xenoplax*

Alarcon-Mendoza, Ivan, C. Khanal and D. Harshman

Clemson University, Dept. of Plant and Environmental Sciences, Clemson, SC 29634

### Abstract

The ring nematode (*Mesocriconema xenoplax*) has become an increasing problem in peach orchard production because of its association with the Peach Tree Short Life (PTSL) disease complex. Because host plant resistance is currently lacking in peach rootstocks and nematicides do not seem to provide a long-term protection, planting poor or non-host cover crops would help manage *M. xenoplax*. Additionally, cover crops improve soil aeration, increase carbon content, and promote antagonistic microorganisms leading to a healthier soil. To determine the host suitability of winter cover crops for *M. xenoplax*, a series of greenhouse experiments were conducted at Clemson University. The crops evaluated were Wrens Abruzii Rye, Saluda wheat, Barley, Triticale, Coker 227 Oat, Australian winter pea, Crimson clover, Balansa clover, Hairy vetch, and Daikon radish, including peach rootstocks Guardian^®^ (control) and MP-29. The experiments were established as a randomized complete block design with five replications. Each crop was grown in 1.5 kg stem-sterilized soil in 15 cm plastic pots and inoculated with 500 *M. xenoplax* per pot a week after planting. The soil was steam sterilized in three cycles of 121^o^C for 45 min prior to filling the pots. Experiments were terminated two months post-inoculation. The reproduction of *M. xenoplax* ranged from 180 to 5,850 individuals per pot, with Australian winter pea and Saluda wheat supporting the greatest and the least reproduction. Nematode reproduction on Hairy vetch, Crimson clover, MP-29, Australian winter pea, and Balansa clover crop was 2955, 3120, 3840, 3930, and 5850, respectively, with reproduction being statistically similar to those on the control. Oat, triticale, and wheat had significantly lower reproduction. The reproduction factor (the ratio of final population and initial inoculum, RF) ranged from 11.7 to 0.4. Balansa clover had the highest RF, while Saluda wheat had the lowest RF. The RF for Hairy vetch, Crimson clover, MP-29, Australian winter pea, and Balansa clover was 5.9, 6.2, 7.7, 7.9, and 11.7, respectively. In addition to Saluda wheat, Coker 227 Oat and triticale had significantly lower RF than the control, with RF being 0.9, 0.72, and 0.4, respectively. Results of the current study demonstrated that the common peach rootstocks Guardian^®^ and MP-29 are good hosts for *M. xenoplax* suggesting a need for the implementation of effective nematode management programs to keep the multimillion-dollar peach industry safe from this nematode. Although planting cover crops in peach orchards is not a common practice, the employment of poor hosts such as oat, triticale, and wheat in peach fields as cover crops can help keep *M. xenoplax* populations below the damage threshold. Additionally, the results from the current study suggest that peach growers should avoid planting cover crops that are good hosts for *M. xenoplax*.

## Erosion of potato resistance to *Globodera pallida*

Amiri, Zahra-Bita and L. M. Dandurand

University of Idaho, Dept. of Entomology, Plant Pathology and Nematology, Moscow, ID 83844

### Abstract

The potato cyst nematodes (PCN), *Globodera rostochiensis* and *G. pallida*, are some of the world’s most important plant-parasitic nematodes, reducing potato yield by 80%. Difficult to control, both species are regulated in the United States by USDA-APHIS. *Globodera rostochiensis* was found in New York in 1941 and *G. pallida* was found in Idaho in 2006. PCN is classified into various pathotypes based on different levels of virulence on several potato genotypes. Although only two pathotypes of *G. rostochiensis* have been found in the US, five pathotypes, Ro1 to Ro5, have been described. According to the Kort classification scheme, three pathotypes, Gpa1-3, of *G. pallida* have been defined. However, according to a scheme devised by Saenz and Scurrah, six pathotypes from South America have been described. Thought to have been spread from Europe, the Idaho *G. pallida* population is a pathotype Pa2/3. The most cost effective and environmentally friendly way of controlling PCN is the use of resistant varieties. Resistant genes such as H1-3, Gro1-4, and Gpa1-5 confer resistance to different pathotype, originated from wild potato cultivars, and have been introgressed into varieties used in Europe but not into russet type varieties suitable for Northwest growers. Of concern is that continuous use of resistant varieties in Europe (Germany and Netherlands) has led to emerging resistance breaking populations of *G. pallida*. The aim of this project is to identify the behavior, fitness, and aggressiveness of progeny *G. pallida* from resistant or susceptible hosts to understand the potential risk of resistance breaking populations for development and deployment of resistance in Idaho.

## Leaf galls induced by *Ditylenchus gallaeformans* support greater bacterial diversity and different species composition than healthy leaves in *Miconia* spp.

Azevedo de Oliveira, Samara^1^, B. J. Campbell^2^, P. Agudelo^1^ and S. J. DeWalt^2^

^1^Department of Plant and Environmental Sciences, Clemson University, Clemson, SC 29634

^2^Department of Biological Sciences, Clemson University, Clemson, SC 29634

### Abstract

*Ditylenchus gallaeformans*, induces several morphological and physiological changes on *Miconia* spp. leaves and it is known that their physical and chemical structures are key factors shaping their bacterial community. Using high throughput 16S rRNA gene amplicon sequencing, we compared the bacterial diversity and species composition of three species of healthy and galled *Miconia* leaves from Trinidad. Our results show higher diversity, species richness, and evenness of bacteria present in galled leaves when compared to healthy *Miconia* spp. leaves. Furthermore, substantially different bacterial composition was observed between healthy and galled leaves and among plant species. We found that the most differentially abundant orders present in galled leaves are Micrococcales, Betaproteobacteriales, Tepidisphaerales, Chitinophagales, Obscuribacterales, Sphingobacteriales, and Xanthomonadales; in healthy leaves, the orders Rhizobiales and Isophaerales were the most differentially abundant. At the family level, *Burkholderiaceae* and *Micrococcaceae* were more abundant in galled leaves than healthy leaves, while *Beijerinckiaceae* and *Isosphaeraceae* were more abundant in healthy leaves. To our knowledge, this is the first study showing bacterial community changes in leaf microbiome due to galls induced by a plant-parasitic nematode.

## Identification and characterization of putative effectors from *Meloidogyne chitwoodi*

Bali, Sapinder^1^, P. Vieira^2^ and C. Gleason^1^

^1^Washington State University, Department of Plant Pathology, Pullman, WA 99164

^2^USDA-ARS, Beltsville, MD 20705

### Abstract

*Meloidogyne chitwoodi* is a root-knot nematode found in potato growing areas of the Northwestern USA. It is a nematode that can infect both potato roots and tubers, and in the case of tuber infections, it causes small pimple-like blemishes on the skin, giving the potato a rough, bumpy appearance and thus adversely impacting the market value of the crop. The goal of this research is to understand how *M. chitwoodi* can successfully infect potatoes. To facilitate the compatible interaction between the host and the nematode, the nematode secretes small molecules called effectors. The effectors are involved in suppressing host defense responses and/or generating and maintaining the nematode giant cells. There are hundreds of predicted secreted proteins in *M. chitwoodi*, but their roles, if any, in parasitism is unknown. Our goal has been to identify and characterize *M. chitwoodi* effectors. Based on transcriptome analyses of pre-parasitic and parasitic *M. chitwoodi*, we have identified genes that contain secretion signal peptide sequences and no transmembrane domains, indicating secretion by the nematode. Next, we used in situ hybridization to localize the effectors’ transcripts to *M. chitwoodi* esophageal glands. Based on BLAST analysis, gland-localized genes that were specific to *M. chitwoodi* were further characterized. Here we show preliminary data on the characterization of two of the nematode genes (Mc1154 and Mc8727). The goal is to functionally validate these putative effectors and use them to pull out their plant interaction partners to further understand their role in parasitism.

## PTI-based callose deposition plays a role in host-nematode interactions

Bali, Sapinder, H. Peng and C. Gleason

Washington State University, Department of Plant Pathology, Pullman, WA 99164

### Abstract

Root-knot nematodes (*Meloidogyne* spp.) are plant-parasitic nematodes that cause billions of dollars of crop loses every year. These microscopic worms infect plant roots and secrete proteins called effectors to manipulate host immune responses and facilitate parasitism. Nematode attack can trigger basal plant defense responses (PTI), including callose deposition, in turn nematodes secrete effectors that manipulate these defense responses to facilitate parasitism. Gleason et al. (2017) characterized an effector from *Meloidogyne hapla* called Mh265. This effector was localized to the esophageal glands of the nematode and was highly expressed during pre- and early parasitic life stages. Transgenic *Arabidopsis* plants constitutively expressing Mh265 were more susceptible to nematode infections and had significantly lesser PTI-based callose deposits. In the present study, we are elaborating our understanding of whether callose deposition plays any significant role in potato-nematode interactions. We found that although Mh265 suppresses the callose deposition response in *Arabidopsis*, it does not directly interact with the plant callose synthases. We treated wild type *Arabidopsis* plants (Col0) with 2-deoxy-D-glucose (DDG), an inhibitor of callose synthesis, and interestingly the plants were more susceptible to *M. hapla* compared to the untreated controls. We then expanded our approach to potato and found that callose synthesis inhibitor, DDG, significantly enhanced its susceptibility to nematode attack. Further, we focused on potato Glucan Synthase gene (StGSL05) because of its significant role in PTI-based callose synthesis and developed *stgsl05* potato knockout lines using RNAi based gene silencing. We have two knockdown lines (*stgsl05*-1.9 and *stgsl05*-3.32) which showed significant enhanced susceptibility to *M. hapla* in nematode assays. Preliminary microscopic evaluations of the transgenic feeding sites showed that the giant cells were much more pronounced (bigger) as compared to those in wild type potato. The long-term goal of the study is to utilize the information to manipulate the deposition of callose or other natural defenses to design nematode control strategies in economically important crops like potato.

## Iron nanoparticle and biological nematicide agent in the management of *Meloidogyne javanica* in soybean

Benedetti, Tatiana^1^ and I. A. Zasada^2^

^1^Department of Horticulture, Oregon State University, Corvallis, OR, 97330

^2^USDA-ARS, Corvallis, OR 97330

### Abstract

*Meloidogyne* spp. have a wide geographical distribution and constitute the most aggressive and economically important genus of nematodes worldwide. Numerous studies have been conducted with the aim to find new tools for nematode management. One such tool is biological control, which has been shown to be less harmful to the environment compared to chemical control. However, the efficiency of biocontrol agents requires precise application of the bacteria to the soil or as a seed inoculation. In addition, other issues can arise such as quality control, short shelf-life, interference of biotic and abiotic factors in the field, and, higher cost of biocontrol agents compared with conventional products. These disadvantages require improvement to maximize biocontrol technical performance. Nanoparticles, due to their specific properties, may represent a tool to minimize the limitations of biocontrol agents; nanoparticles have also shown potential in the management of plant diseases. This research aimed to: a) synthetize iron oxide nanoparticle from environmental waste, b) evaluate the potential of nanoparticles to improve nematode control in combination with a biocontrol agent, and c) measure reactive oxygen species (ROS) as a possible mode-of-action by which the iron nanoparticle controls nematode. The addition of iron nanoparticle at a concentration of 75 mg/kg enhanced the effect of the biocontrol agent by improving the control of *Meloidogyne javanica*, resulting in a 88% reduction in the density of nematode females on the roots of soybean. The mechanism that promoted *M. javanica* mortality appeared to be linked to the generation of ROS. Co-inoculation of iron nanoparticles and a biocontrol agent demonstrated good potential as a *M. javanica* management strategy in soybean.

## Digital drawings of nematodes – from camera lucida to adobe illustrator

Bernard, Ernest C.^1^ and B. Im^2^

^1^University of Tennessee, Department of Entomology & Plant Pathology, Knoxville, TN 37996

^2^Pennsylvania State University, Department of Plant Pathology and Environmental Microbiology, State College, PA 16801

### Abstract

Accurate, informative illustrations of nematodes and their relevant morphological details are necessary components in species descriptions, higher-order revisions and instructional materials. Most nematode since the beginning of the science have been line drawings made either free-hand or with the aid of an optical device such as a camera lucida. Interestingly, a review of drawings over the past century seems to indicate that drawings of small soil nematodes are more accurately rendered than those of large animal parasites, due in large part to more easily observed features. However, many papers have been illustrated with drawings of strongly flattened specimens or those distorted by preservation in alcohol. Classic drawing perfection, not since equaled nor surpassed, is found in those by N. A. Cobb’s associate, W. E. Chambers, but good illustration can be achieved without Chambers’ skill set. Studying how features are emphasized and how layering is achieved in good illustrations will improve one’s own ability without taking graphic arts courses. In addition, many imaging tools now exist to provide deeper understanding of morphology. *En face* views and very fine body details are easily obtained with scanning electron microscopy (remember glycerin jelly?). Differential interference and confocal microscopy provide the means to examine and image nematodes layer by layer to achieve a deeper understanding of internal morphology; these images can be assembled into plates or translated into drawings to provide emphasis on the most important features. Adobe Illustrator is a major advance in illustration because of its vector graphics, which allow images to be scaled up or down without compromise to its dimensions. The scale bar is also easily incorporated with help of the microscope software, which uses hardware position and objective lens to calculate magnification. Additionally, Adobe’s cloud storage autosaves work and allows for remote access. One might think that this way is superior to the conventional method of hand drawing, but there are pros and cons. Without some background in digital media, it may be tough to achieve a finer level of detail. However, the option of combining photo editing software like photoshop after making the initial line drawing could enhance the depth and detail of the final image. Once complete the drawings can easily be combined into a tile set or saved as any file format.

## Seeking order out of chaos – *Thelastoma* spp. (Oxyuridomorpha), commensals in arthropod intestines

Bernard, Ernest C.^1^, G. J. Phillips^1^, C. T. McAllister^2^, J. K. Moulton^1^, J. Turner^1^ and J. Kolape^3^

^1^University of Tennessee, Department of Entomology & Plant Pathology, Knoxville, TN 37996

^2^Science and Mathematics Division, Eastern Oklahoma State College, Idabel, OK 74745

^3^University of Tennessee, Advanced Microscopy and Imaging Center, Science and Engineering Research Facility, Knoxville, TN 37916

### Abstract

The genus *Thelastoma* contains about 70 described species, most of which are commensal in the intestine of millipedes, cockroaches or beetle larvae, with single species described from mole crickets, scorpions and earthworms. This number represents nearly 9% of all oxyuridomorph nematodes. A few species have been reported from millipedes and beetles, millipedes and cockroaches, or beetles and cockroaches. Most cockroach-inhabiting species have been described from India, largely from *Periplaneta americana* (American cockroach). Males have been described for about three-fourths of the species. Numbers of specimens studied for the descriptions varies from 1 to 97. The members of this genus are extraordinarily similar in overall morphology and morphometrics; species have been separated largely on the basis of body, esophagus and tail lengths, and length and width of the esophageal basal bulb. None of the descriptions contain statistical analysis of variability within characters. Dissection of several *Narceus americanus* (American giant millipede), collected from a single locality in southeastern Oklahoma, at the far western end of its distribution, yielded numerous specimens of *Thelastoma* that initially appeared to represent several species based on tail shape and length criteria. However, molecular analysis of individuals encompassing the full range of tail lengths and shapes established that they all represented a single species, one we consider undescribed. This result casts doubt on the use of tail shape and length as a primary criterion for distinguishing species in this genus. Therefore, we constructed tables summarizing all the data provided in the original descriptions for each species, as well as data presented for species mentioned in subsequent papers. Few papers had all of the standard measurements and ratios typically reported for new species in modern treatises, but we were able to calculate many of them from the illustrations. In some cases, text and illustrations could not be reconciled, so calculations were made from the latter, which we considered likely more accurate. A large proportion of papers failed to adequately compare purported new species with existing ones, or comparisons lacked rigor. The “a” value (body length/width) appeared to often be calculated from flattened specimens, so we disregarded this morphometric value. Among the potentially useful characters are the width of the first body annule, shape of the basal stomal teeth, and the V’ ratio (length of head to vulva/head to anus), which eliminates tail length variability. In all cases, suspected new taxa or even those believed to be already identified need to be examined molecularly. Unfortunately, so few *Thelastoma* species have been characterized molecularly that neither barcoding nor phylogenetic studies are feasible at the present time.

## Pathotype and virulence of *Globodera* species from Peru to assess diversity of South American populations

Bhatta, Bhupendra and L. M. Dandurand

Department of Entomology, Plant Pathology and Nematology, University of Idaho, Moscow, ID 83844.

### Abstract

*Globodera pallida* and *Globodera rostochiensis*, the two species of potato cyst nematodes, are quarantine pests that are detrimental to potato production. Both *G. pallida* and *G. rostochiensis* are native to South America and have spread to the major potato growing areas worldwide. In the U.S., *G. rostochiensis* was first detected in New York in 1941 and *G. pallida* was detected in Idaho in 2006. There are potato varieties that are resistant to *G. rostochiensis*, and New York has a strategy in place to contain and control this pest. A program to contain and eradicate *G. pallida* in Idaho exists since its detection. Soil fumigation has been the major component of the eradication program in Idaho. However, ban of the effective fumigants such as methyl bromide has led to search for alternative control strategies. Currently, efforts to develop *G. pallida* resistant russet potatoes varieties are underway. *Globodera pallida* and *G. rostochiensis* have different pathotypes and development of the varieties resistant to these diverse pathotypes is desirable. Furthermore, an understanding of their genetic diversity is essential for development of broad-spectrum resistance in the event of a new introduction and for the durability of resistance. This work aims to evaluate the genetic diversity of the *Globodera* species, which is crucial for plant breeders to select an effective, broad-spectrum, and durable source of resistance. For this, our objective is to identify the *Globodera* species collected from 10 fields of Peru and assess their pathotype and virulence. Identification of the *Globodera* species was done by morphological and molecular methods. Differential potato clones, which are potato clones with different resistance genes, have been used to distinguish between *Globodera* species with different virulence genes affecting their reproduction. We conducted a bioassay with potato varieties/clones having different sources of resistance to *Globodera* species for pathotype and virulence assessment. After 8 weeks of growth, all life stages of the nematode in the roots were enumerated. Number of females and relative susceptibility score compared to a susceptible potato cultivar Desiree was used to evaluate the virulence and pathotype of the populations. Multiplex PCR results identified populations from 7 fields as *G. pallida* and 3 as a mix of *G. pallida* and *G. rostochiensis*. Our bioassay results indicate that *Globodera* populations are highly diverse, and as a group contain more pathotypes and a wider degree of virulence than what was encompassed by the resistance genes that are available for breeders to use in the U.S. The narrow selection of genetic resistance available to U.S. breeders places the potato industry at further jeopardy to these invasive pests were another introduction to be made. Since it may take more potato clones with different resistance sources to fully represent this diversity, we are conducting trials using additional potato clones.

## Impact of soybean (PI 437654) after wheat harvest on soybean yield in cyst nematode-infested fields in Michigan

Bird, George

Michigan State University, College of Agriculture and Natural Resources, Department of Entomology, East Lansing, MI 48824

### Abstract

Various non-cash crop plants have been used for management of Heteroderidae species including *Globodera rostochiensis*, *G. pallida* and *Heterodera schachtii*. This, however, has not been done for *H. glycines* (SCN). Soybean (PI 437654) was selected as a non-cash crop plant for seeding soon after wheat harvest in SCN-infested fields to determine its impact on yield of the next soybean cash crop. Six fields are being used in the research. Three of the sites had SCN populations greater than 1,000 per 100 cubic centimeters (cm^3^) of soil and three had SCN populations less than 1,000 per 100 cm^3^ soil. The experimental design consists of replicate strips of PI 437654 and fallow planted in late summer after wheat harvest. Site size ranges from about 0.10 to 54 acres. The PI 437654 was killed by the first fall frost following planting. The sites were planted to soybeans the following spring or the year after a year of corn. One of the three sites with the higher population density of SCN will not be planted to soybeans until 2025. There was no significant difference in the bean yields between the PI 437654 and fallow in the three fields with SCN population densities less than 1,000 per 100 cm^3^ of soil. Bean yields, however, were significantly greater following PI 437654 compared to fallow in the two fields with population densities of SCN greater than 1,000 per 100 cm^3^ of soil. While PI 437654 may have utility for use as a non-cash crop plant for management of SCN under Michigan conditions, there are at least four potential limiting factors. These include: 1) the lack of readily available PI 437654 seed, 2) having an appropriate stand of the non-cash crop plant for an impact on SCN, 3) the fact that following wheat, most SCN-infested fields in Michigan have relatively low populations of SCN and 4) the potential risk of development of aggressive populations of SCN that reproduce on and result in low bean yields of future PI 437654-derived soybean cultivars.

## Potato nematodes of North America: with special reference to soil health dynamics

Bird, George

Michigan State University, College of Agriculture and Natural Resources, Department of Entomology, East Lansing, MI 48824

### Abstract

Potatoes are the number one non-grain food crop. In North America they are grown on about 1.5 million acres with an annual farm value greater than $4 billion. Annual average tuber yields among the ten Proveniences and eighty-two states of Canada, Mexico and the United States range from less than 150 to more than 600 cwt per acre. The below ground architecture of the potato plant is unique and consists of shoot (stem, stolon and tuber) and root (basal, nodal, stolon and tuber) tissue. More than twenty species of plant parasitic nematodes have been formally reported as associated with potatoes in North America. Four of these, *Globodera rostochiensis, G. pallida, G. ellingtonae* and *Ditylenchus destructor* are regulated quarantine species. In addition, six *Meloidogyne* spp. and *Nacobbus aberrans* invade tuber tissue and cause extensive hyperplastic symptoms. As migratory endoparasites, *Pratylenchus* spp. function as predisposition agents for the potato early-die disease complex and Trichodoridae spp. vector the tobacco rattle virus that causes corky ringspot. In some potato growing regions, most of the acreage is fumigated or treated with a non-fumigant nematicide. In thirty potato field trials conducted between 1974 and 2012, potato tuber yields were an average of 114 and 60 cwt greater following soil fumigation or non-fumigant nematicide application, respectively, compared to the non-treated control. In a 2011 soil health study of 48 fumigated and 48 non-fumigated commercial potato fields, *P. penetrans* populations, active carbon, aggregate stability and nitrogen mineralization potential were significantly less following soil fumigation, compared to the non-fumigated fields. When 68 of the fields were re-evaluated in 2022, using the 2011 geo-positioned sampling points, all of the fields had increases in soil organic matter, active carbon and soil water holding capacity, indicating that individual indicators can be useful in studying the dynamics of soil health.

## Papaya ground seed as a biofumigant against root-knot and reniform nematodes in Hawai’i

Braley, Lauren and K. -H. Wang

University of Hawaii at Manoa, Honolulu, HI 96822

### Abstract

Plant-parasitic nematodes (PPN) such as root-knot nematodes (RKN; *Meloidogyne* spp.) and reniform nematodes (RN; *Rotylechulus reniformis*) pose a serious threat to food security, causing an estimated $80 billion in agricultural losses annually. Growers in Hawaii are especially challenged by PPN pressure all year round. Rising cost of agriculture production inputs is calling food producers in Hawaii to adopt sustainable pest management tactics using local resources. Papaya seeds are considered an agricultural waste in Hawaii. Objectives of this study were to examine the potential use of ground papaya seed (PGS) against RKN and RN, as PGS contains benzyl isothiocyanate (BITC), a biofumigant that is suppressive to many soil-borne pathogens. Three greenhouse trials were conducted to compare 3 PGS treatments (0.5% PGS, 1% PGS, and 0.5% PGS combined with 0.5% boiled water PGS crude extract [PGS+CE]) to a 1% ‘Calientee 199’ brown mustard (*Brassica juncea*) soil amendment (BM) as a positive control, and a non-amended soil (NA) and an autoclaved soil (Auto) as negative controls. Field soil known to be free of plant-parasitic nematodes was used. Each treatment was replicated 4 times. In Trial I, 3 ‘Manoa’ lettuce (*Lactuca sativa*) seedlings were planted right after soil amendment, in Trial II and III, ‘Hirayama’ kai choi (*Brassica juncea*) seedlings and ‘Manoa’ lettuce seedlings, respectively were planted 1 week after soil amendment. In all treatments, except for the autoclaved control, 100 *Meloidogyne incognita* infective juveniles were inoculated into each pot at planting and the experiment terminated 1 month after nematode inoculation. An additional greenhouse trial was conducted to compare PGS amendments (0.5% PGS and 1% PGS) to non-amended, artificially inoculated soil (NA) and an autoclaved control (Auto). Each treatment was replicated 4 times using sterile sand: soil mix. ‘Black Eye #5’ cowpea (*Vigna unguiculata*) seedlings were planted 1 week after soil amendment, followed by inoculating 200 *R. reniformis* vermiform nematodes into each pot. The experiment was terminated 6 weeks after RN inoculation. In all the *M. incognita* trials, significant reduction in root gall formation was found for all PGS treatments compared to the non-amended treatment, with PGS treatments being comparable to BM and autoclaved treatments. In Trial II, through acid fusion staining, a reduction in number of *M. incognita* infecting the roots was also observed in the 1% PGS and PGS+CE roots. In the *R. reniformis* trial, through acid fusion staining, a reduction in RN females was observed in PGS-treated roots compared to the non-amended treatment. This research presents a viable biofumigant alternative, using BITC produced by PGS against RKN and RN infection on kai choi, lettuce, and cowpea. PGS also provides the added advantage of being compatible with organic production while avoiding the needs of an extensive plant growth period when using biofumigant cover crops (such as brown mustard) prior to soil amendment.

## Effects of summer and fall cover crop combinations on sting nematode, stubby root nematodes, and freeliving nematodes in potato

Budhathoki, Sabina and Z. J. Grabau

Department of Entomology and Nematology, University of Florida, Gainesville, FL, 32611

### Abstract

Sting (*Belonolaimus longicaudatus*) and stubby-root nematodes (primarily *Nanidorus minor* and *Nanidorus obtusus*) are major plant-parasitic nematodes (PPNs) limiting the yield of spring potatoes (*Solanum tuberosum*) in northeast Florida. There are limited nematode management options in this area and farmers rely on chemical nematicides. Cover cropping is a non-chemical approach which may affect PPNs and soil ecology. A field trial was conducted in 2022 to determine the efficacy of integrating summer and fall cover crops for sting and stubby-root nematode management as well as impacts on the free-living nematode community. The experiment had a split plot arrangement with summer cover crops as main plot treatments and fall cover crops including controls as sub-plot treatments. Summer cover crops included 1) sunnhemp (*Crotalaria juncea* cv. Crescent Sun) and 2) sorghum-sudangrass (*Sorghum bicolor* × *S. sudanense* cv. SX19) terminated and incorporated in soil 4 months after planting. After that, fall cover crops treatments were implemented: 1) caliente mustard (*Brassica juncea* cv. Rojo), 2) arugula (*Eruca sativa* cv. Nemat), 3) weedy fallow, and 4) weedy fallow followed by fumigation using 1,3-D at 66.2 kg a.i./ha at 25 days prior to potato planting. Fall cover crops were grown for approximately 2 months then terminated and incorporated into soil. Potatoes were planted 3 weeks after fall cover crop termination. Nematode soil abundances were assessed at termination of each cash/cover crop and at midseason of potato production. Summer cover crops did not significantly affect sting, stubby-root and free-living nematode populations in summer or fall. However, after fall cover crops, sting and stubby-root nematode abundances were significantly greater on arugula compared to fallow (73 and 85%, respectively). Similarly, caliente mustard significantly increased sting and stubby-root nematode abundances compared to fallow (51 and 60%, respectively). Total free-living nematodes, fungivore and bacterivore abundances were significantly higher in arugula than other cover crops while omnivore and predator abundances were not significantly different across the treatments. There was no summer by fall treatment interaction in summer or fall. These initial results indicate that arugula and caliente mustard may be hosts for sting and stubby-root nematodes, but arugula cover cropping could also improve soil health as shown by increased free living nematode populations.

## Searching for natural enemies of plant-parasitic nematodes in California

Casey, Veronica

University of California, Davis, Department of Entomology and Nematology, 367 Briggs Hall, One Shields Avenue, Davis, CA 95616

### Abstract

Plant-parasitic nematodes pose a significant threat to agriculture as they feed on plant tissues leading to reduced nutrient uptake and scar tissue formation on the roots. Some of the most destructive nematodes include root-knot nematodes (*Meloidogyne* spp.), lesion nematodes (*Pratylenchus* spp.), and ring nematodes (*Criconema* spp.). The plant’s first line of defense begins in the soil rhizosphere, where an active microbiome can suppress infection by pathogens. While synthetic chemicals, resistant crops, soil solarization, and crop rotation are commonly used to mitigate nematode problems, these methods can be ineffective and harmful to the environment and workers. With toxic nematicides like methyl bromide being phased out, sustainable alternatives are urgently needed. Biological control, which utilizes natural enemies in the ecosystem, is an increasingly favorable option. Promising candidates that naturally antagonize plant-parasitic nematodes may be found in suppressive soils. My research focuses on identifying a potential biological control agent that targets plant-parasitic nematodes. Starting with almond orchards in California, I will extract soil samples, isolate plant-parasitic nematodes, and identify the bacteria associated with these nematodes using 16S rRNA gene sequencing. Additionally, I will use a soil solution to bait bacteria with native nematodes and with lab cultures of *Meloidogyne* spp. After identifying the nematode-associated bacteria, I will conduct bioassays to evaluate any nematicidal activity. Finally, I aim to uncover the molecular pathway involved in the biological control agent’s nematicidal activity. This project will test the hypothesis that suppressive soils contain microbes that exhibit nematicidal activity by: 1) characterizing nematode-associated microbes in soil, 2) identifying bacteria with antagonistic effects on plant-parasitic nematodes, and 3) understanding the molecular mechanisms of control.

## Trade offs between resistance breaking and fitness cost in root-knot nematodes

Coomer, Alison^1^, P. Shakya^1^, M. Winter^3^, D. Lunt^3^, V. M. Williamson^1^ and S. Siddique^2^

^1^University of California Davis, Dept. of Plant Pathology, Davis, CA, USA

^2^University of California Davis, Dept. of Entomology and Nematology, Davis, CA, USA

^3^School of Nature Natural Sciences, University of Hull, Hull, UK

### Abstract

Root-knot nematodes (RKNs), are among the most devastating pathogens of crops, causing substantial yield and economic losses worldwide. These parasitic organisms can infect over a hundred different plant species and can evade plant defense mechanisms by secreting a concoction of effectors. For decades, the *Mi-1* resistance gene has been effective in detecting and inhibiting RKNs in tomatoes. However, the underlying mechanisms by which *Mi-1* detects these pathogens remain largely unknown. In recent years, resistance-breaking populations have emerged in both greenhouse and field settings, posing a threat to the potency and effectiveness of the *Mi-1* gene and, consequently, the tomato industry. We used two strains of *M. javanica*, one strain VW4, which is recognized by *Mi-1*, and another strain, VW5, which was selected from VW4 and can overcome resistance mediated by *Mi-1*. Utilizing the newly constructed reference genome for *M. javanica* (VW4), we compared genomes of VW4 and VW5 and identified an approximately 650 kb region that is present in VW4 but missing in VW5. This missing region contains ten protein-coding genes, three of which encode putative effectors and are currently being tested as potential avirulence genes for *Mi-1*. In addition, we have conducted a series of infection assays on different host plants lacking *Mi-1,* and the results revealed a significantly lower egg count in VW5 when compared to VW4. We plan to expand these assays by testing additional *M. javanica* resistance-breaking strains collected from fields all over California to determine if this trade-off is consistent across other strains. Overall, our results suggest that although VW5 can overcome *Mi-1*, there is a trade-off in the form of compromised reproduction. This research helps to better understand the mechanism and components of *Mi-1* and develop strategies for addressing resistance-breaking populations.

## Is resistance in plant-parasitic nematodes a concern with new-generation nematicides?

Crow, William T.

University of Florida, Entomology and Nematology Dept., Gainesville, FL 32611

### Abstract

Plant-parasitic nematodes are considered to have low risk for developing nematicide resistance and until recently there have been no documented cases of nematicide resistance in plant-parasitic nematodes. However, the recent discovery of reduced sensitivity to fluopyram in plant-parasitic nematodes from golf greens in Florida following repeated application of fluopyram nematicide has brought resistance concerns to the forefront. Most older-generation nematicides are either fumigants or acetylcholinesterase inhibiting non-fumigants with modes-of-action that are not conducive to resistance. However, new-generation nematicides tend to have more targeted activity and in some cases the mode-of-action is unknown, resistance may be of greater concern for these chemistries. Factors such as persistence, movement, application frequency, application method, and non-nematicide applications of the same active ingredient or actives with similar modes-of-action can impact the development of resistance. This paper will examine these factors and how they can be managed to reduce the likelihood of resistance. It is critical that there be cooperation between researchers and extension specialists to identify situations where nematicide resistance is likely to occur and help develop and implement nematicide rotation and other integrated pest management tactics for these situations.

## Systems approach to controlling nematodes in U.S. potato production

Dandurand, Louise-Marie^1^, I. Zasada^2^, C. Gleason^3^, J. Kuhl^4^, W. S. DeJong^5^ and P. Watson^6^

^1^875 Perimeter Drive MS 2329, University of Idaho, Moscow, ID

^2^USDA-ARS, 3420 NW Orchard Ave, Corvallis, OR

^3^1772 NE Stadium Way, Washington State University, Pullman, WA

^4^Plant Science Department, University of Idaho, Moscow, ID

^5^1110 Bradfield Hall, Ithaca, NY

^6^Ag Economics Department, University of Idaho, Moscow, ID

### Abstract

Among the many nematodes threatening potato production in the U.S., the potato cyst nematodes (PCN) *Globodera pallida* and *Globodera rostochiensis*, and the root knot nematodes (RKN) *Meloidogyne chitwoodi* and *Meloidogyne hapla*, continue to pose serious threats to productivity. RKN can infect tubers and cause cosmetic damage that reduces potato market value, whereas PCN are quarantined pests in the U.S. and, if left uncontrolled, can cause 80% yield loss. Since there are few or no potato varieties resistant to PCN or RKN, growers must rely on nematicides as the most effective means for control. Unfortunately, many front-line nematicides have been banned or voluntarily withdrawn from the market while others have suffered supply chain problems. Most nematicides also pose substantial environmental risks to applicators and bystanders, which can lead to disruption of soil biology. Development of potato varieties with nematode resistance, discovery of novel nematicidal compounds, establishment of damage thresholds, and decision support systems are critical needs for management of nematodes. For potato to be sustainably produced, a robust strategy for controlling these devastating pests must be implemented. Recently funded by the NIFA-SCRI program, our goal for this coordinated agricultural project is to develop a systems approach to control plant-parasitic nematodes that threaten the potato industry. To achieve this, our objectives are four pronged: 1) develop decision support tools by fast tracking diagnostic methods and developing predictive models to assist in development of an action plan for farmers dealing with nematode infestations; 2) increase our understanding of plant defenses and use markers to develop resistant varieties; 3) discover and develop novel nematicides; and 4) pass our information on to benefit all sectors of the potato industry that are impacted by these devastating pests.

## Evaluating alternative management strategies for the root lesion nematode, *Pratylenchus penetrans*, and the root-knot nematode, *Meloidogyne hapla*, in specialty root cropping systems

Darling, Elisabeth^1^, S. Thapa^1,2^, L. Parrado^1^, K. Poley^1,3^, H. Chung^1^ and M. Quintanilla-Tornel^1^

^1^Michigan State University, Department of Entomology, East Lansing, MI 48824

^2^Corteva Agrisciences, 9330 Zionsville Road, Indianapolis, IN 46268

^3^Corn Marketing Program of Michigan, Lansing, MI, 48906

### Abstract

Infestations of two important plant-parasitic nematode genera, *Pratylenchus* and *Meloidogyne*, can result in severe yield reductions and unmarketable characteristics of specialty root vegetable crops. The grower standard, oxamyl, is a toxic nematicide that may be subject to increased environmental bans in the future, which emphasizes the need to investigate alternative strategies, including: less toxic chemically based nematicides, biologically based nematicides, manure-based compost soil amendments, and cover crop incorporation. To evaluate these potential management options, a series of small plot field trials were established in Michigan carrots and parsnips. In greenhouse trials, several oilseed radish varieties were evaluated for their host status for both *P. penetrans* and *M. hapla*. *Pratylenchus* nematodes pose a significant risk to carrot production and other root vegetables, but challenges associated with species identification, culturing, and limitations of field studies can exacerbate problems with managing them. In parsnips, *M. hapla* populations were sufficiently managed with fluensulfone, heat-killed *Burkholderia* spp., and oxamyl. Additionally, incorporating one of the poor host cover crops of *P. penetrans, M. hapla*, or both, may be an effective technique for controlling populations between seasons.

## Using marker-assisted selection to transfer *Meloidogyne incognita* resistance in sorghum

Davis, Richard F.^1^, K. R. Harris-Shultz^1^, J. E. Knol^1^ and H. Wang^2^

^1^USDA-ARS, P.O. Box 748, Tifton, GA 31793

^2^USDA-ARS, 4007 Throckmorton Hall, Manhattan, KS 66506

### Abstract

*Meloidogyne incognita* is a wide-spread and damaging pathogen of many important crops in the southern United States, and most sorghum genotypes allow significant levels of reproduction by the nematode. A series of greenhouse evaluations were conducted to determine whether a quantitative trait locus (QTL) that imparts a high level of resistance to *M. incognita* in sorghum can effectively be transferred into diverse sorghum genotypes using marker-assisted selection. Using marker-assisted selection, the resistance QTL, QTL-Sb.RKN.3.1, from ‘Honey Drip’ sorghum was crossed into five different sorghum backgrounds that included forage, sweet, and grain sorghum until the BC_1_F_6_ generation. Repeated greenhouse experiments documented that the recurrent parent genotypes were all susceptible to *M. incognita* and statistically similar to each other. In contrast, the BC_1_F_6_ genotypes were all highly resistant and similar to each other and similar to the resistant standard, ‘Honey Drip’. These results suggest that this resistance QTL could be introgressed using marker-assisted selection into many sorghum genotypes and confer a high level of resistance to *M. incognita*. Thus, this QTL and its associated markers will be useful for sorghum breeding programs to incorporate *M. incognita* resistance into their sorghum lines.

## Foliar nematode distribution and characterization in ornamental plants in Florida

Demesyeux, Lynhe, A. Habteweld and W. T. Crow

University of Florida, Dept. of Entomology and Nematology, Gainesville, FL 32608

### Abstract

Foliar nematodes of the genus *Aphelenchoides* can cause severe damage to plants. Reported symptoms include vein limited leaf lesions, leaf crinkling, bud and leaf abscission, and reduced plant vigor. Although not usually lethal to their hosts, they can cause substantial economic losses in crops sold for their aesthetic value such as ornamentals. In Florida, ornamental production is a $21 billion industry and plant parasitic *Aphelenchoides* spp. represent a major threat to this industry due to their wide host range. To characterize the foliar nematodes in Florida ornamentals, a survey of botanical gardens was conducted across the state in Summer 2022 in 6 different counties (Alachua, Polk, Pinellas, Lake, Gadsden, and Indian River). From each site, samples were collected from plants showing suspected foliar nematode symptoms. Each sample was stored and processed separately. Nematodes were extracted from infected leaves by incubating them in water. In Alachua County, *Echinacea* spp. [FL-Isolate-2 and FL-Isolate-7], *Rudbeckia* spp. [FL-Isolate-9], *Davallia fejeensis* [FL-Isolate-9], and *Dryopteris erythrosora* tested positive for *Aphelenchoides*. In Polk County *Aphelenchoides* were recovered from *Gerbera* spp. [FL-Isolate-3FBT], *Berlandiera lyrata* [FL-Isolate-5EBT], *Rudbeckia* spp. [FL-Isolate-7], and *Arachniodes* spp. In Lake County *Aphelenchoides* were recovered from *Verbena* spp. and *Salvia rosmarinus*. *Aphelenchoides* were isolated from *Rudbeckia* spp. [FL-Isolate-3G, FL-Isolate-3H, FL-Isolate-4C, FL-Isolate-4D] in Gadsden County and from *Orchidacea* in Indian River County. No *Aphelenchoides* were found from the botanical Gardens in Pinellas County. Eight to 10 nematodes were randomly selected from each of these plants and were subjected to DNA extraction and PCR analysis. Molecular sequence analysis using the D2–D3 expansion segments of 28S sequences identified all tested nematodes as *A. pseudobesseyi.* The Florida *A. pseudobesseyi* populations in the present study showed 98 to 100% sequence homology with several of *A. pseudobesseyi* sequences from the GenBank such as MT271871 and MT271870 from *Dryopteris erythrosora* and *Farfugum japanicum* in Florida, respectively, and MT271872 from North Carolina. The phylogenetic analysis of D2–D3 grouped all the Florida populations collected during the survey in a separate clade closer to the other *A. pseudobesseyi* populations obtained from the GenBank. These results indicate that the recently described *A. pseudobesseyi* may be the most common foliar nematode infecting ornamental plants in Florida.

## Nematode management in Florida Organic Strawberries – a mountain to climb

Desaeger Johan^1^, H. Bui^1^, D. Moreira^1^, C. Oliveira^1^, J. Carter^1^ and W. Elwakil^2^

^1^University of Florida, Department of Entomology and Nematology, Gulf Coast Research and Education Center, Wimauma, FL 33598, USA

^2^University of Florida IFAS Extension, Hillsborough County, Seffner, FL, 33584

### Abstract

Organic agriculture is in its infancy in Florida, but interest is growing. Especially for strawberries; consumer demand of organically grown fruits is increasing, and many major retailers are now expecting Florida strawberry growers to provide them with a certain percentage of organic fruit in addition to non organic fruit. As a result, organic strawberry production has seen a rapid increase in the past 5 years, and currently accounts for about 10% of the total strawberry acreage (~ 4,856 ha) grown in Florida. Strawberry production in Florida is valued at over US$350 million, and the state accounts for almost all of the strawberry winter production in the US. Most strawberry production in Florida occurs in open field and on plastic-mulch raised beds in combination with drip irrigation. This so-called plasticulture system provides an ideal habitat for nematodes like root-knot (*Meloidogyne hapla*) and sting (*Belonolaimus longicaudatus*), which are widely distributed throughout Florida and are one of the major constraints to overall strawberry production in Florida. Soil fumigation is the standard practice in conventionally managed strawberry fields, and in most cases provides good control of sting nematodes. Organic growers do not have this option, and in recent years sting nematodes have emerged as one of their most serious and difficult to manage problems. Several growers have abandoned their organic fields because they are simply unable to manage sting nematodes. It is clear that organic growers in Florida face a formidable challenge trying to manage sting nematodes and other soilborne diseases in their fields. There is limited experience with organic production in Florida and especially research into organic commercial strawberry production has been lacking. If organic food production systems are to become a reality in Florida, more effective non-fumigant pest management options are needed, which will require a significant increase in biologically-based soilborne disease and nematode management research. A recently established organic certified field at the University of Florida Gulf Coast Research and Education Center (GCREC) is a first step in building such a program. Currently, the research is focused on investigating integrated nematode management practices, including (1) cover crops, (2) host resistance, (3) biologically-based soil disinfestation practices, and (4) organic nematicides. Opportunities and limitations of these different practices will be discussed.

## Assessment of soybean cyst nematode suppressive soil from soybean fields in North Dakota

Dhakal, Roshan, A. Plaisance and G. Yan

North Dakota State University, Department of Plant Pathology, Fargo, ND 58108

### Abstract

The soybean cyst nematode (SCN; *Heterodera glycines*) is a damaging pest affecting soybean production. Nematicides, resistant soybean cultivars, and crop rotation are the common means to control SCN, but these methods have their own limitations. Therefore, alternative approaches such as identification and use of suppressive soil are important to manage SCN. Nematode-suppressive soil does not support nematode establishment and is also transferrable. The objective of this study was to identify and investigate SCN-suppressive soil in North Dakota (ND). Initially, 23 soybean fields previously recorded with low SCN populations were sampled for SCN densities and 10 fields with very low SCN populations (0–6 eggs per cc of soil) were selected for further greenhouse experiments. The experiment was conducted in a randomized complete block design with four replications in a growth chamber for 60 days. The susceptible soybean cultivar Barnes was used as the host plant and the SCN population from Richland County was used as inoculum. The SCN cysts, eggs, and juveniles were recorded, and reproductive factors were calculated to assess the reproduction of SCN in the soils. The results from the first two trials showed that four field samples out of 10 produced few SCN (0–10 eggs per cc of soil) in non-inoculated natural field soil. Two of the field samples HG 21-2A and HG 64 showed the potential to suppress SCN as the SCN population was reduced in the inoculated natural field soil compared to the control, inoculated autoclaved field soil. However, mixing the field soil with autoclaved field soil in a 1:10 ratio did not significantly lower the SCN population in the autoclaved field soil for either of them. To further investigate the transferrable trait of the soil, a third trial was conducted in which HG 64 significantly reduced SCN cysts, eggs, juveniles, and reproductive factors in the autoclaved field soil when the field soil was mixed with the autoclaved soil in a 1:1 ratio. These findings indicated that HG 64 was both SCN-suppressive and transferrable. The repetition of the trial is being carried out to validate the results. Microbiome analysis for the microbes that may be involved in decreasing SCN population in these field soils is also underway. For that purpose, DNA was extracted from the field soils before setting up the experiment and after harvest at 60 days. The DNA samples were sent out for sequencing of the V3-V4 region of 16S rRNA for bacteria and the ITS1 region of ITS-rDNA for fungi present in those soils. The analysis will give insights into the microbiome diversity that may be responsible for SCN suppressiveness. This research provides valuable information on the potential and mechanisms of certain field soils to suppress SCN and may be used by the industry and growers in developing sustainable SCN management strategies.

## AI and Big data applications in nematology

DiGennaro, Peter and S. Mishra

University of Florida, Department of Entomology and Nematology, Gainesville, FL 32611

### Abstract

The significant increase in adoption of -omics technologies to address questions in plant nematology has generated an extensive library of large-scale public datasets. New bioinformatic resources has made it feasible to process and analyze such large and complex data to address questions of nematode parasitism on a broader scale. Concurrently, considerable strides in the accessibility and practicality of artificial intelligence and machine learning have opened novel paths to address old issues and revolutionize plant nematology. Here I will explore two potential applications of these incredible resources and explore the capacities needed to fully leverage and appreciate the changes AI and big data are bringing to plant nematology. First, we gathered numerous RKN-host transcriptome datasets from the public domain and developed novel algorithms that specifically tackle cross-species interactions between host and nematodes. After accessing the structure and distribution pattern of all the data, we curated the datasets for quality, and generated data formats required for multi-omics downstream analyses. Elucidating the common plant pathways that could be a target for nematode management is of interest, so the datasets spanning multiple hosts and nematode species were integrated for analyses. Second, we annotated thousands of nematodes from high resolution images of samples sent into the UF Nematode Assay Lab and employed several object detection machine learning algorithms to evaluate the potential of AI-based plant parasitic nematode detection and quantification. Where else can such applications drive innovation in nematology? How can we prepare for and leverage these powerful tools? There is a clear need for capacity building and training on utilizing this next generation of tools.

## Entomopathogenic nematode proteins modulate host immunity during infections

Dillman, Adler R.^1^, O. K. Okakpu^1^, S. C. Parks^2^, A. K. Lima^1^, S. Martinez-Beltran^1^ and P. Azizpor^1^

^1^Department of Nematology, University of California, Riverside, 900 University Ave., Riverside, CA 92521

^2^Department of Microbiology and Immunology, Stanford University, Stanford, CA 94305

### Abstract

Nematode parasites release excretory/secretory (ES) products to manipulate host biology. The complex mixture of nematode ES usually contains hundreds of proteins. Although nematode ES has been shown to have immunosuppressive effects on various hosts, our understanding of the effects of individual proteins remains limited. Entomopathogenic nematodes (EPNs) release a complex mixture of hundreds of ES proteins during the infection of insects, and many of these proteins are conserved in the ES of nematode parasites of plants and vertebrates. We present the results of studying several individual proteins from *Steinernema carpocapsae* ES from different protein families including secreted phospholipase A2 enzymes (sPLA_2_), fatty acid- and retinol-binding proteins (FAR), and Shk-domain-containing proteins. Our data suggest that individual proteins contained in EPN ES have potent immunomodulatory effects on host insects and that EPNs are a powerful model for understanding parasitic nematode ES in general.

## Virulence diversity of soybean cyst nematode in Minnesota

Docherty, Lauren^1^, A. Lorenz^1^ and S. Chen^2,3^

^1^University of Minnesota, Department of Agronomy and Plant Genetics, St. Paul, MN 55108

^2^University of Minnesota, Department of Plant Pathology, St. Paul, MN 55108

^3^University of Minnesota, Southern Research and Outreach Center, Waseca, MN 56093

### Abstract

Soybean cyst nematode (SCN) (*Heterodera glycines*) is the most damaging pest affecting soybean crops, causing an estimated $1 billion in yield loss annually. The most effective method for managing SCN is the use of resistant soybean cultivars. However, SCN populations have the ability to overcome soybean resistance in what is known as a virulence shift. Nearly all soybean cultivars available in Minnesota use a single source of resistance, PI (Plant Introduction) 88788. This reliance on a single source leaves farmers at risk of virulence shifts and losing their ability to manage this pest. This project examines the virulence diversity of SCN in Minnesota to better understand the potential for virulence shifts. This will be done using 180 inbred lines of SCN descended from field populations. These field populations were originally collected from soybean fields in Minnesota. The SCN lines were inbred for at least ten generations to increase their homogeneity and improve test repeatability. These inbred lines are being tested for their virulence on 9 soybean lines with diverse resistance genes. Many of these soybean lines are not part of the standard virulence test, thus their effectiveness is unknown and may include highly effective resistance to a broad sample of SCN populations. For this test, SCN eggs are inoculated on a soybean seed and placed in a growth room for 35 days; long enough for the nematodes to complete a life cycle. The resulting adult females (cysts) are extracted and counted, and compared to cyst counts on a susceptible soybean plant to calculate a female index (FI). The FI is a measure of how successfully an SCN line can infect and reproduce on a soybean line. Results obtained so far indicate that many of the SCN lines can overcome the resistance provided by PI 88788. PI 90763 shows the most promise, with very few SCN lines overcoming its resistance. The results also show that there is little overlap in virulence among different soybean lines, suggesting different virulence genes are needed. The information provided by this project will help farmers make management decisions and help soybean breeders develop cultivars with durable SCN resistance. The data from this project will also be used in conjunction with genomic data to identify regions of the SCN genome associated with virulence.

## Making digital mosaic micrographs of nematodes

Eisenback, Jonathan D.

School of Plant and Environmental Sciences, Virginia Tech, Blacksburg, VA 24061

### Abstract

Amazing images of nematodes are possible with digital cameras that are attached to research quality microscopes. These digital photomicrographs are the primary means of recording images of nematodes; however, not all microscopists take advantage of the digital format. The most life-like and clearest images come from specimens that are paralyzed rather than killed with heat. Likewise, the nematodes that are mounted on agar pads instead of other mounting methods are most natural and are also usually level and lateral. These mounts serve as a foundation for making high resolution micrographs of nematodes utilizing several additional techniques that take full advantage of the digital image. Several images can be taken along the full length of the nematode and stitched together to form one mosaic photograph. Also, several high-resolution images, taken while focusing through the specimen, can be stacked into one image with amazing levels of focus; and for some specimens, this stack of images can be exported as a three-dimensional model of that specimen. Finally, digital imaging with high dynamic range (HDR) can be used to increase the contrast in images that have a wide variation in brightness. Digital images are systematic resources that can have a big impact on the taxonomy of nematodes when used with good technique.

## Modeling and printing 3D models of nematodes

Eisenback, Jonathan D.

School of Plant and Environmental Sciences, Virginia Tech, Blacksburg, VA 24061

### Abstract

Three-dimensional (3D) models of nematodes that utilize a 3D printer for research and educational resources have rarely been used in nematology. Modeling nematodes requires knowledge about their morphology, artistic ability, and an aptitude for using complicated 3D modeling software that usually have very steep learning curves. Beginning with a light or scanning electron micrograph, a realistic 3D model of a nematode can be made with the “lathing” tool in Zbrush® modelling software. Additional details can be added to the model with various brushes in the software palette, and the finalized model can be exported in a file format that is compatible with a 3D printer. Seven basic types of printers are widely used: 1) material extrusion, 2) vat polymerization, 3) powder bed fusion, 4) material jetting, 5) binder jetting, 6) directed energy deposition, and 7) sheet lamination. Material extrusion and vat polymerization have the widest application and comparisons of models printed by each of the two methods will be demonstrated. The software files of each of the nematode models will be added to the SON website and made available for members of the society to use for teaching and extension. Three-dimensional models of nematodes can make a major contribution to the systematics and taxonomy of nematodes.

## Is the adoption of regenerative soil management practices to increase soil organic matter always good for sustainability of temperate orchards and vineyards? A nematological perspective

Forge, Tom

Agriculture and Agri-Food Canada, Summerland Research and Development Centre, Summerland, British Columbia V0H 1Z2, Canada

### Abstract

As interest in regenerative agricultural practices grows, producers of temperate perennial fruit crops are increasingly adopting the use of between-row cover crops and organic amendments to enhance soil organic matter and various aspects of soil health. The increase in soil organic matter inputs and enhanced overall soil biological activity resulting from such practices is often claimed to enhance suppressiveness to soil-borne pests. The objective of this presentation is to review research on how populations of plant-parasitic nematodes (PPNs) of concern for temperate orchards and vineyards actually respond to organic amendments and cover crops being promoted to enhance orchard and vineyard soil health. The available data, which are generally limited to field experiments with timeframes of one to five years, indicate that: 1) organic amendments can result in increased PPN population densities, 2) responses of PPN populations to any given type of amendment can be context-dependent (e.g. soil nutrient levels, mode of irrigation), and 3) amendments suppressing one nematode species can result in enhanced population densities of another species. For example, the use of a livestock manure compost in a sweet cherry orchard over 10 years initially suppressed populations of *Pratylenchus penetrans* but then fostered growth of a *Mesocriconema xenoplax* population in the longer-term. Temperate legumes are good hosts for many PPNs of concern for orchards and vineyards, and their inclusion in alley cover crop mixes, to simultaneously offset the use of mineral fertilizers and enhance organic matter buildup, can also lead to increased population densities of PPNs. Collectively these results indicate that there can be negative longer-term ramifications for orchards and vineyards of adopting practices to optimize soil organic matter buildup without also considering their effects on PPNs. Longer-term studies are needed to determine if soil organic matter-enhancing practices that increase PPN populations in the short term might also result in suppressiveness in a longer time-frame.

## Comparative Mitochondrial Genomics in nematoda: astonishing variation in compositional strand biases and mutation rates driven more by life traits than phylogeny

Gendron, Eli. M.^1^, Q. Xue^2^, J. L. Sevigny^3,4^, H. Li^2^, Z. Liu^2^, E. King^5^, M. Blaxter^5^, T. O. Powers^6^, W. K. Thomas^3,4^ and D. L. Porazinska^1^

^1^Department of Entomology and Nematology, University of Florida, Gainesville, FL

^2^Nanjing Agricultural University, China

^3^Molecular, Cellular, and Biomedical Sciences, University of New Hampshire, Durham, NH

^4^Hubbard Center for Genome Studies, University of New Hampshire, Durham, NH

^5^Wellcome Sanger Institute, Cambridge, UK

^6^Department of Plant Pathology, University of Nebraska, Lincoln, NE

### Abstract

Nematodes are the most ubiquitous, abundant, and diverse metazoans on Earth known to affect ecosystem functioning. A better understanding of their biology and ecology including adaptations to diverse habitats (e.g., soils, marine sediments, or vertebrate hosts) and lifestyles (e.g., free-living, predatory, or parasitic) is key to understanding their response to global change scenarios. Mitochondrial genomes offer high species level representation, low cost of sequencing, and an ease of data handling that can provide insights on nematode evolutionary pressures. Here we examined 271 mitochondrial genomes spanning the entire Nematoda phylum to test the effects of specific factors including phylogeny and life traits on their characteristics. We sourced 223 published genomes from the International Nucleotide Sequence Database Collaboration, but also expanded their phylogenetic range by sequencing additional 48 genomes from new species. Genomes and protein coding genes (PCGs) were analyzed for multiple characteristics with a special focus on nucleotide composition, usage frequency, coding strand compositional skew, nucleotide substitution rates and gene order. Synonymous (dS) and non-synonymous (dN) substitution rates were determined using phylogenetic trees constructed under gene and codon specific substitution rates. We observed strong strand biases with a trend of an increasing G skew from lineages known for more ancestral traits towards lineages with more recently derived traits, particularly in the 3^rd^, and to some extent the 1^st^ codon positions. However, the strand bias profiles exemplified an astonishing variation in skew that were highly clade-specific (e.g., high GC skews in Rhabditida), PCGs-specific (e.g., high GC skews in NAD3 and NAD4L), and even codon position-specific (e.g., high GC skew in the 3^rd^ codon position). Similarly, there was a wide range of variation of both types of substitutions that were both taxon- and PCGs-specific without much of phylogenetically informed patterns. In contrast, strand biases showed significant relationships with nematode feeding habits, with parasitic (animal and plant) species showing strong codon position specific G skews. This was somewhat mirrored in habitats where animal-associated species tended towards higher G skews within their PCGs compared to the free marine/terrestrial species. Interestingly, generally lower rates of non-synonymous substitutions but also higher rates of synonymous substitutions within PCGs were observed in the parasitic nematodes compared to many microbivores. In conclusion, our study suggests that nematode mitochondrial genomes undergo uneven and phylogenetically independent, but likely lifestyle driven pressures. This could provide a hypothesis for why their use for resolving deep branches may be limited, as opposed to resolving close relationships.

## Efficacy of bionematicides against *Meloidogyne incognita* under greenhouse and field conditions

Gitonga, Denis and A. Hajihassani

University of Florida, Dept. of Entomology and Nematology, Fort Lauderdale Research and Education Center, Davie, FL 33314

### Abstract

*Meloidogyne incognita* causes severe damage to most vegetable crops. Due to environmental, health, and regulatory concerns over chemical nematicides, bionematicides could be an effective control approach against *M. incognita*, but little is known about the general efficacy of these products on plant-parasitic nematodes. Greenhouse and field experiments were conducted to evaluate the nematicidal efficacy of commercially available bionematicides against *M. incognita*. The greenhouse experiment screened eight bionematicides: Majestene, TerraNeem, AzaGuard, Molt-X, NemOmex, EcoWorks, Monterey, and Promax in 10 cm diam. plastic pots planted with tomatoes and laid out in a completely randomized design with seven replicates per treatment. Each pot was filled with sterile field soil, inoculated with 2,500 second-stage juveniles (J2), and mixed well to evenly distribute the nematodes in the soil. The bionematicides were soil-drenched three times, at planting and 10 and 28 days after planting. After eight weeks, plants treated with AzaGuard and Majestene had the highest (*P* < 0.05) fresh root weight, while plants treated with Monterey had the lowest root weight compared to negative and positive controls. Plants with Monterey had the lowest (*P* < 0.05) galling index, followed by that with AzaGuard and Molt-X, compared to the positive control. Furthermore, plants treated with AzaGuard and TerraNeem had the lowest (*P* < 0.05) and the highest eggs per gram of roots compared to the positive control. AzaGuard, EcoWorks, NemOmex, Promax, and Pendi were selected for field experiments, and Vydate was used as the standard control. Two plasticulture field trials were conducted in a field naturally infested with *M. incognita*. The trial consisted of two bionematicide application regimes (calendar vs. nematode development based). The experiment utilized a randomized complete block design with five replicates per treatment. The average daily soil temperature at a depth of 5, 10, and 20 cm was assessed for the nematode development-based application. Accumulated degree days of 370 to 380 were achieved for *M. incognita* to complete one generation, at which the nematicides were applied according to the label through the drip irrigation system. Soil nematode abundances were assessed three times at pre-plant, midseason, and harvest, while root galling was assessed at mid-season and harvest. The crop yield was also determined by harvesting cucumber three times during the season. Nematode development-based applications had lower (*P* < 0.1) *M. incognita* density at harvest than calendar-based applications. Moreover, AzaGuard, EcoWorks, and Vydate had the lowest nematode density (*P* < 0.1) compared to other treatments. The gall index and yield differed significantly among all treatments in both application regimes. These results suggest that the nematode development-based application reduces *M. incognita* population density and the frequency of application compared to calendar-based applications. Additionally, AzaGuard and EcoWorks consistently reduced *M. incognita* population density under greenhouse and field conditions; therefore, they can be used in an integrated pest management approach to control *M. incognita*.

## Comparing a traditional soil sampling to an automated precision soil sampler (APSS) to efficiently collect and quantify soybean cyst nematode

González, Richard and H. D. Lopez-Nicora

The Ohio State University, Dept. of Plant Pathology, Columbus, OH 43210

### Abstract

Soybean cyst nematode (SCN), *Heterodera glycines*, the most economically important soybean pathogen in the United States, can cause drastic yield losses. Active management of SCN begins with soil sampling to know current population densities. Commonly, soil samples for SCN testing are collected manually. Although this manual sampling is effective, it has some limitations, such as being time-consuming, sometimes difficult to cover soybean fields of large dimensions, and consequently, costly. This project aimed to evaluate the efficiency of an automated soil sampler compared to traditional hand sampling to quantify the SCN populations in 100 cm^3^ of soil. One field, infested with *H. glycines*, was chosen in Clark County, Ohio, to evaluate both methods. In collaboration with Integrated Ag Services, an automated precision soil sampler (APSS) was used for the automated sampling. For the traditional method, a cylindrical stainless-steel soil probe was used to collect 7–8 cores around the automated sampling lines. A total of 366 samples were collected in a grid pattern with 14 m and 4 m spacing among sampling points, divided into 183 samples for each method. Soil volume ranged from 30 to 100 cm^3^ for the APPS and 100 cm^3^ for the traditional soil sampling. These samples were elutriated, ground through a 60-mesh sieve, and stained to determine the number of eggs per 100 cm^3^ soil. Number of SCN eggs from samples that did not reach that amount, were standardized to100 cm^3^ of soil. A concordance correlation coefficient (CCC) analysis was used to measure agreement between the two methods. CCC ranges from −1 to 1, with 1 representing perfect positive agreement, a value of 0 showing perfect disagreement, and −1 demonstrating perfect inverse agreement. The result obtained by CCC analysis showed a significant positive relationship for the number of SCN eggs between the soil samples collected by APPS and those taken using traditional soil sampling (*P* < 0.05, *r* = 0.69). This result indicates the possibility of utilizing technologies like the APPS in extensive soybean fields to estimate SCN population densities. The use of automated precision soil sampler machines will facilitate rapid soil sampling to efficiently evaluate SCN populations in soil, identify areas at risk, and actively manage SCN in infested fields.

## Multi-year, field evaluations of fumigant and non-fumigant nematicides for management of *Meloidogyne enterolobii* in sweetpotato production in North Carolina

Gorny, adrienne, P. B. Jeffreys, K. Cox and J. Dotray

Department of Entomology and Plant Pathology, North Carolina State University, Raleigh, NC 27695

### Abstract

*Meloidogyne enterolobii* is an emerging root-knot nematode pathogen in vegetable and field crop production in the Southeastern United States, and is of particular concern on sweetpotato. Due to the current lack of market-accepted sweetpotato cultivars in the U.S. with resistance to *M. enterolobii* and limited non-host rotational crop options, management in the field relies heavily on the use of chemical nematicides. To support economic and environmentally sustainable use of these nematicide products, chemical nematicides (representing fumigant, non-fumigant, registered, and experimental products) were evaluated in the field for their efficacy in managing *M. enterolobii* in sweetpotato in North Carolina in 2021 and 2022. Field evaluations were performed in small plot replicated trials. Sweetpotato (cvs. ‘Covington’ and ‘Beauregard’) were transplanted and nematicide products applied either before (fumigants) or at transplanting (non-fumigants). Sweetpotatoes were maintained according to standard agronomic practices for North Carolina. Plots were harvested, storage roots graded for size, and data on yield and storage root galling severity were collected. Soil samples were collected from each plot at harvest and root-knot nematode populations were quantified. Fumigant nematicides that numerically reduced incidence of nematode damage and increased marketable yield included 1,3-dichloropropene (e.g., Telone II) and metam sodium (e.g., Metam CLR 42%) products, while non-fumigant nematicides that numerically reduced incidence of nematode damage and increased marketable yield included fluopyram (e.g., Velum Prime) and fluazaindolizine (e.g., Salibro) products. Although there were minor to moderate numerical improvements in yield and damage metrics, these differences were not statistically significant in all cases. Results from 2021 and 2022 indicate that future research may benefit from studies on nematicide application timing, rates, methods (e.g. row application vs. broadcast) and consideration of edaphic factors such as soil temperature and moisture. Reflections on lessons learned from the field for improved sweetpotato nematicide testing are also included.

## Do *Meloidogyne arenaria* and peanut leaf spot pathogens have synergistic effects in peanut production?

Grabau, Zane^1^, R. Barocco^2^, R. Sandoval-Ruiz^1^, N. Dufault^3^ and I. Small^2^

^1^University of Florida, Entomology and Nematology Dept., Gainesville, FL 32611

^2^University of Florida, North Florida Research and Education Center-Quincy, Quincy, FL 32351

^3^University of Florida, Department of Plant Pathology, Gainesville, FL 32611

### Abstract

*Meloidogyne arenaria* (peanut root-knot nematode, PRKN) is the most problematic nematode on peanut (*Arachis hypogea*) in the Southeast. Similarly, early leaf spot (*Passalora arachidicola*) and late leaf spot (*Nothopassalora personata*) are the primary foliar pathogens on peanut in the region. Certain nematode-fungal pathogen combinations are known to have synergistic effects that increase crop disease, but the interaction for PRKN and leaf spot pathogen—and, more practically, the interaction of management practices for these pests—needs investigation. The objective of this research was to: 1) evaluate effects of fungicides and PRKN-resistant cultivars on PRKN management, and 2) determine if infection by leaf spot pathogens increase PRKN disease in peanut production. To test this objective, small plot field trials were conducted in Quincy, FL and Live Oak, FL. Cultivars resistant (‘TifNV HighO/L’) and susceptible (‘GA 06G’) to PRKN were evaluated as the main-plot in a randomized complete block split-plot design. Foliar fungicide regime was the sub-plot treatment, which included: 1) untreated, 2) reduced schedule (5 applications), and 3) full schedule (7 applications). Both full and reduced fungicide regimes helped manage peanut leafspot, and ‘TifNV HighO/L’ also managed disease better than ‘GA 06G’. The resistant cultivar, but not fungicides, managed PRKN reproduction and root system damage (galling). The lack of significant fungicide effects on PRKN suggests there was not a strong synergistic effect of peanut leafspot pathogens on PRKN disease. For both cultivars, the reduced and full fungicide regimes increased yield by 35–48% relative to no fungicide. Similarly, the resistant cultivar had 49–54% greater yield than the susceptible, indicating PRKN also affected yield. While these pests may not be synergistic, this study showed that managing both pests is needed to optimize peanut production in the Southeast.

## Influence of subtropical regenerative cropping systems on plant-parasitic nematodes are species-specific

Grabau, Zane

University of Florida, Entomology and Nematology Department, Gainesville, FL 32611

### Abstract

The long growing season in the subtropical climate of the southeastern United States lends itself to the development of a diversity of cropping systems. In particular, in recent years, alternative cropping systems that incorporate cash or cover crops in the fall or winter seasons, when land is typically fallow, have emerged as alternatives to traditional cropping systems. Because they add organic carbon to agroecosystems, these alternative cropping systems can be considered regenerative. Plant-parasitic nematodes are a ubiquitous problem in the Southeast, so impacts of alternative cropping systems on plant-parasitic nematodes can influence grower adoption. This presentation will focus on the influence of two regenerative cropping systems: 1) sod-based rotation that incorporates perennial bahiagrass into annual peanut/cotton production, and 2) winter *Brassica carinata* (carinata) production integrated into annual row crop production. Diversifying a cropping system using sod-based rotation typically improves management of major plant-parasitic nematodes in the Southeast, particularly reniform nematode (*Rotylenchulus reniformis*). Integrating carinata into cropping systems also provides comparable control of reniform nematode relative to more prevalent practices such as winter fallow or small grain cover crops. However, carinata and other brassicas can be detrimental for managing other plant-parasitic nematodes, such as stubby-root nematodes (*Nanidorus* spp.). Regenerative cropping systems can help manage plant-parasitic nematodes, but not universally for all nematodes. Because the most important endemic plant-parasitic nematodes vary by geographic region, this emphasizes the need for region- and system-specific plant nematology research when new regenerative cropping systems are introduced.

## Management of *Meloidogyne enterolobii* in sweetpotato using fumigant and non-fumigant nematicides

Grabau, Zane and C. Liu

University of Florida, Entomology and Nematology Dept., Gainesville, FL 32611

### Abstract

*Meloidogyne enterolobii* has emerged as a substantial problem in sweetpotato production in the southeastern United States. In the short term, nematicide application is one of the main options for managing this nematode. Additionally, non-target effects of nematicides on free-living nematodes are increasingly becoming a consideration when developing management strategies. The objectives of this research were to evaluate fumigant and non-fumigant nematicides for: 1) *M. enterolobii* management, and 2) non-target impacts on free-living nematodes. A replicated (n = 6) small plot field trial was conducted in north central Florida in 2022. A common sweetpotato cultivar (Covington) that is moderately-resistant to *Meloidogyne incognita*, but is susceptible to *M. enterolobii* was grown. Treatments included 1) untreated control, 2) fluensulfone at 1.97 kg/ha, 3) fluensulfone at 3.93 kg/ha, 4) fluazaindolizine at 2.23 kg/ha, 5) 1,3-dichloropropene (1,3-D) at 88.5 kg/ha, and 6) fluopyram at 249 g/ha. Fluensulfone, fluazaindolizine, and fluopyram were applied as broadcast drench at transplanting as liquid formulations. The 1,3-D fumigation treatment was a broadcast shank application at two weeks before planting. Fumigation using 1,3-D was the only nematicide treatment that significantly (Fisher’s protected LSD, α = 0.05) reduced *M. enterolobii* soil abundances relative to untreated at midseason (53 days after planting), but there were no differences in *M. enterolobii* soil abundances at harvest. At harvest, tuber surface galling was significantly less for fluensulfone, fluazaindolizine, and 1,3-D treatments than untreated, but fluopyram was not different from untreated. Total tuber yield was significantly greater for either fluensulfone treatment or 1,3-D than untreated. Only 1,3-D fumigation increased marketable tuber yield relative to untreated. However, 1,3-D and fluensulfone at 3.93 kg/ha significantly reduced midseason free-living nematode soil abundances relative to untreated, although this effect dissipated by harvest. Based on this trial, nematicides can help manage *M. enterolobii*, with 1,3-D fumigation being the most effective option, but fluensulfone and fluazaindolizine also having efficacy. This trial will be repeated in 2023 to validate results.

## Interactions between HG types of the soybean cyst nematode and the different α-SNAPs of the rhg1 resistance

Haarith, Deepak and A. Bent

University of Wisconsin-Madison, Dept. of Plant Pathology, Madison, WI 53726

### Abstract

We developed stable T4 soybean cis-genics that over-express a copy of either the *rhg1-a* or *rhg1-b* type α-SNAP under a soybean ubiquitin promoter in *rhg1-a* type background soybean plants. When challenged with soybean cyst nematode (SCN, *Heterodera glycines*), we had observed earlier that the cis-genics significantly reduced the female index (FI) for a HG 0 population and a HG 2.5.7 population compared to the parental background. However, we did not observe any change in FI with respect to a HG 1.3.6.7 population. In greenhouse trials, we did not observe any significant difference in seed quality in terms of seed protein, moisture, and oil, nor difference in quantity in terms of seed weight with our HG 2.5.7 SCN. Recently, we observed significant changes in the levels of *rhg1-a* type α-SNAP protein accumulation in infected roots amongst the different HG type populations compared to un-inoculated roots. This reveals clues on how α-SNAPs interact with SCN at protein levels. We are currently studying relative expression of the α-SNAPs and other related genes using qPCR, to dissect the differences between the pathways employed by the different α-SNAPs in resisting SCN HG types and their regulation.

## Grafting tomato to manage root-knot nematode and southern blight disease

Hajihassani, Abolfazl^1^ and A. P. Keinath^2^

^1^Department of Entomology and Nematology, Fort Lauderdale Research and Education Center, University of Florida, Davie, FL 33314

^2^Plant and Environmental Sciences Department, Coastal Research and Education Center, Clemson University, Charleston, SC 29414

### Abstract

Root-knot nematodes (RKN; *Meloidogyne* spp.) and southern blight (SB) disease, caused by the soil basidiomycete *Athelia rolfsii* (formerly *Sclerotium rolfsii*), are present throughout the southeastern United States causing damage to tomato (*Solanum lycopersicum*). These two pests have been managed mainly with soil fumigation or fungicide/nematicide applications. One promising alternative to these control practices is grafting tomato onto rootstocks resistant to soilborne pathogens. In greenhouse experiments, we previously characterized a high level of resistance in *S. sisymbriifolium* (sticky nightshade) against multiple RKN species including *M. incognita*, *M. arenaria*, *M. haplanaria*, and *M. enterolobii*. This study aimed to determine the effects of grafting tomato on *S. sisymbriifolium* used as a rootstock to control southern blight and RKN in plasticulture systems. Separate field trials using *A. rolfsii* and *M. incognita* were conducted in 2020 and repeated in 2021. In the SB study, tomato cultivar Roadster was grafted on three cultivars/lines (White Star, Diamond, and SisSynII) of *S. sisymbriifolium* to examine the incidence of SB disease. In the RKN study, Roadster was grafted only on the line SisSynII and treated with four non-fumigant nematicides (Nimitz^®^, Velum Prime^®^, Vydate^®^, and Majestene^®^) applied through drip irrigation systems. The susceptible Roadster tomato was included in both studies as non-grafted and self-grafted treatments for comparison. In the SB study, all rootstocks significantly reduced the incidence of southern blight disease and increased crop vigor ratings compared to control treatments. White Star and SisSynII rootstocks significantly increased marketable weight compared to control treatments. In the RKN study, the rootstock SisSynII and Red Bounty (an RKN-resistant cultivar used as control) reduced root galling and soil population density of RKN more than self- and non-grafted treatments. There was no difference in marketable tomato weights among the rootstock, self-, and non-grafted treatments, regardless of which nematicide was used. Results showed the high potential of the *S. sisymbriifolium* rootstock to manage two widely distributed soilborne diseases of tomato production in the southeastern United States to minimize the use of harmful pesticides.

## On a new species of needle nematode *Longidorus* (nematoda: Longidoridae) from Maryland and California, USA

Handoo, Zafar^1^, M. Kantor^1,2^ and S. Subbotin^3^

^1^Mycology and Nematology Genetic Diversity and Biology Laboratory, USDA, ARS, Beltsville, MD 20705

^2^Plant Pathology & Environmental Microbiology Department, The Pennsylvania State University, University Park, PA 16802

^3^Plant Pest Diagnostic Center, California Department of Food and Agriculture, 3294 Meadowview Road, Sacramento, CA 95832

### Abstract

Needle nematodes, *Longidorus* spp. are polyphagous or obligate root ectoparasites of many plants. In 2020, during a survey of natural grass, tall fescue, *Lolium arandinaceum* (Schreb.) Darbysh *Longidorus* spp. was found along the bank of the Western Branch of Patuxent River in Upper Marlboro, MD. Females and juveniles of an undescribed needle nematode were recovered from soil samples using the sugar centrifugal flotation and Baermann funnel extraction methods. Female body length ranged from 3.8 to 5.1 mm having a truncate, set off lip region which was slightly swollen anteriorly. The odontostyle measured (77–92 μm), and odontophore (40–53 μm). The vulva was located at 46–50% and tail was conoid to bluntly rounded. No males were found. This new *Longidorus* species is morphologically similar to *L. breviannulatus*, *L. elongatus*, *L. martini*, *L. americanus, L. grandis, L. sabalanicus* and *L. sturhani* but differs from these species either by the odontostyle, odontophore, total stylet length and in having a set off lip region, which is slightly swollen/rounded laterally. The amphideal fovea was pocket-shaped without lobes at base and a broadly rounded peg like tail terminus was observed. Phylogenetic analysis of 28S rRNA gene sequences placed this new *Longidorus* species in a clade with *L. litchi, L. fangi, L. jonesi, L. diadecturus* and *Longidorus* sp. The 28S rRNA sequence of this new *Longidorus* species was similar to that of *Longidorus* sp. collected from *Juglans* sp. growing in Butte County, California, USA. Morphological observations and molecular analysis of the 28S rRNA, ITS1 rRNA and *COI* gene sequences indicated that the specimens collected from soil around the rhizosphere of grass from Upper Marlboro, MD and Butte County, CA represents a new needle nematode species.

## Construction of esophageal connectome of *Heterodera* glycines

Han, Jaeyeong, G. Warner, U. Reuter-Carlson and N. E. Schroeder

University of Illinois at Urbana-Champaign, Dept. of Crop Sciences, Urbana, IL 61801

### Abstract

The soybean cyst nematode (*Heterodera glycines*) is a devastating soybean pathogen. Feeding behavior in *H. glycines* is essential for its survival and reproduction. Understanding the underlying neural mechanisms can provide insights into the development of targeted control strategies. However, there is limited information available on the *H. glycines* esophageal nervous system that regulates its feeding behavior. In this study, we aimed to investigate the esophageal anatomy and connectivity of the infective second-stage juvenile (J2) of *H. glycines* using electron microscopy (EM). We utilized high-pressure freezing followed by freeze substitution to fix *H. glycines* J2s. Ultrathin (50–70 nm) serial longitudinal and transverse sections of the anterior region were collected and imaged using scanning electron microscopy with a backscatter detector to acquire TEM-like high-resolution images. Initially, we successfully collected and imaged longitudinal serial sections. We are currently working on collecting and imaging ultrathin cross serial sections. Subsequently, neuronal cells, synapses, gap junctions, and neuronal processes will be identified and annotated to reconstruct the esophageal anatomy including synaptic connections. We expect the results to reveal the neuronal classes and connectivity within the esophageal nervous system of *H. glycines*, providing insights into the structural basis of *H. glycines* feeding behavior. This study will help advance the understanding of the esophageal nervous system of *H. glycines* and its role in feeding behavior, contributing to the development of a novel targeted control strategy.

## Characterization of a soybean mitogen-activated protein kinase kinase that regulates defense response during soybean cyst nematode infection

Hawk, Tracy, S. Piya, S. Zadegan, P. Li, J. Rice and T. Hewezi

The University of Tennessee, Dept. of Plant Sciences, Knoxville, TN 37996

### Abstract

Mitogen-activated protein kinase cascades play important roles in plant defense response. Once such MAP kinase cascade, that includes the MAP kinase kinase 2 (MKK2), has been thoroughly studied in *Arabidopsis* and found to both negatively and positively regulate plant immune signaling. The soybean ortholog GmMKK2 was recently identified as a major soybean kinase hub gene whose expression is differentially expressed in the soybean cyst nematode feeding site, the syncytium. To evaluate the role of GmMKK2 in the relationship between soybean and soybean cyst nematode, we generated and overexpressed a kinase-dead mutant using the transgenic hairy root system. Overexpression of inactivated MKK2 resulted in a dramatic reduction in soybean susceptibility to soybean cyst nematode, whereas overexpression of the endogenous GmMKK2 had no effect on susceptibility. RNA-seq analysis revealed that in response to soybean cyst nematode infection, both MKK2 variants displayed distinct gene expression changes. The analysis also revealed that a unique set of genes may be under negative regulation of GmMKK2. Among these genes were many involved in salicylic acid signaling and reactive oxygen species ROS production. To understand how GmMKK2 mediates transcriptional reprogramming, we analyzed phosphoproteomes regulated by each variant under noninfected and soybean cyst nematode-infected conditions. It appears that GmMKK2 mediates defense response through regulation of a number of transcription and translation factors. GmMKK2 also facilitates downstream signaling through activation of several protein kinases and phosphatases. Taken together, our results indicate that GmMKK2 is critical for the compatible interaction between soybean and soybean cyst nematode by negatively regulating plant defense and immunity signaling pathways.

## Phosphonate soil treatments improve tree health and suppress foliar nematode populations in American beech with beech leaf disease

Herms, Daniel A.^1^, D. R. Volk^2^, C. E. Hausman^2^, A. Persad^3^ and B. L. Holko^1^

^1^The Davey Tree Expert Company, Kent, OH, 44240

^2^Cleveland Metroparks, Parma, OH, 44134

^3^ACRT Services, Stow, OH, 44224

### Abstract

American beech (*Fagus grandifolia*) exhibiting symptoms of beech leaf disease were treated with soil applications of potassium phosphite from 2017–2022. Treated trees on average had 3-fold greater canopy cover as untreated control trees, and half the number of fine dead twigs and branches. Potassium phosphite treatments also reduced densities of foliar nematodes presumed to be the causal agent by 20-fold relative to densities in untreated trees.

## Investigating pathogenicity and competition in co-infestations of *Globodera ellingtonae* and *Globodera pallida* on russet potato

Hickman, Paige and L. M. Dandurand

University of Idaho, Dept. of Entomology, Plant Pathology, and Nematology, Moscow, ID 83844

### Abstract

*Globodera ellingtonae* was first described in 2012 and has been found in Oregon and Idaho potato fields. Although it has high reproduction on potato, it is not currently regulated like the closely related species *G. pallida* and *G. rostochiensis*. Initial studies on *G. ellingtonae* found that it did not cause yield loss even at high infestation levels. However, the effects of co-infestations between *G. ellingtonae* and other potato cyst nematode species have not yet been evaluated. *Globodera pallida* is a quarantine pest in Idaho because it is a major threat to the Idaho potato industry. It is important to further evaluate the risk associated with *G. ellingtonae* as it is also present in Idaho. This research seeks to investigate the competitive interactions between *G. ellingtonae* and *G. pallida*. To determine impact of co-infestations, Russet Burbank was inoculated with single and co-infestations of *G. ellingtonae* and *G. pallida* at 0, 5, 10, and 20 eggs per gram of soil. Data was collected on tuber yield, aboveground biomass, root mass, and progeny cysts. Preliminary results show that while increasing progeny cysts correlated with increasing infestation levels, there was not significant yield loss. It is important to further assess yield impact of these species; data from a different study suggest that under certain conditions, Russet Burbank is tolerant of *G. pallida* at high infestation levels despite prolific reproduction. In the first trial results, *Globodera pallida* alone had more reproduction than *G. ellingtonae* alone. Further analysis using quantitative PCR will determine proportions of each species in mixed infestation samples to assess whether one species is able to outcompete the other and to what extent. It is essential to continue to study *G. ellingtonae* to elucidate any risks associated with it alone or in co-infestations with *G. pallida*.

## Biosolarization with recycle olive waste as a new tool to control plant parasitic nematodes

Hodson, A.K., Jungo-Garcia, A., Simmons, C. and Fernandez-Bayo, J.D.

University of California, Department of Entomology and Nematology, Davis, CA 95616

### Abstract

Olive pomace, comprised of olive meat, seed and skin, is currently considered a waste byproduct of oil production. Simultaneously, perennial crops need alternative strategies to manage plant parasitic nematodes as chemical intervention has become increasingly regulated. Using pomace for pest suppression could solve two problems at once, reducing waste products and controlling nematode pests. Biosolarization, a process which combines solar heating and microbial activity, likely intensifies these effects, creating fermentation products from the pomace break down which act as natural fumigants. This study evaluated olive pomace, both alone and combined with biosolarization, as a strategy to manage plant parasitic nematodes. In a microcosm experiment, simulated biosolarization with pomace reduced *Meloidogyne incognita* survival when applied at scaled down rates of 4, 8 or 16 wet tons/acre compared to untreated controls. A field experiment was also conducted comparing: 1) biosolarization with pomace, 2) tarp alone 3) pomace alone, 4) traditional pre-plant fumigation with Telone, and 5) an untreated irrigated control. Fewer *M. incognita* inoculated into cloth bags were recovered from plots that had been fumigated, tarped only, or had been biosolarized, than those that had received pomace only. In addition to *M. incognita*, other plant parasitic nematodes in the family Tylenchidae were also common in the bags. Compared to controls, both fumigation and biosolarization reduced Tylenchidae recovery. When all nematodes in the bag were counted, including free living bacterial and fungal feeders, fumigation reduced nematode recovery compared to all other treatments. These experiments suggests that biosolarization does not have strong effects on the overall nematode community, like fumigation, but reduces plant parasites in a more targeted way, while olive pomace alone has little effect.

## *Paratylenchus* spp. management and impact in daylily (*Hemerocallis* spp.) ornamental plants in Michigan

Howland, Amanda D. and M. Quintanilla

Michigan State University, Department of Entomology, East Lansing, MI

### Abstract

Michigan is the third largest producer in the United States’ ornamental plant industry and is the second largest producer of herbaceous perennial plants. Within the herbaceous perennial plants category, daylily (*Hemerocallis* spp.) is a major component, with an economic value of $16.8 million in 2020. A major pathogen affecting the ornamental plant industry is plant-parasitic nematodes. In Michigan, *Paratylenchus* spp. are commonly found in ornamental plant fields. Even though a small greenhouse trial testing the host status of daylily against a known host, sweet corn, was inconclusive, since *Paratylenchus* spp. is known to be a problem in other ornamental plants, such as anthurium, research on how to effectively manage these nematodes in ornamental plant fields needs to be conducted. Therefore, a three-year field trial was established to determine potential management options. The field trial was established at a commercial nursery in Zeeland, MI, in a field with a high *Paratylenchus* spp. population. Six treatments were applied each spring to the plots; treatments included were 1) Indemnify Root Dip (plants dipped in Indemnify before planting) (Dip) + TerraClean 5.0 soil drench, 2) Dip + Indemnify soil drench, 3) Dip + AzaGuard soil drench, 4) Dip + 101 Starter Blend Compost, 5) 101 Starter Blend Compost by itself, and 6) an untreated control. Treatments were arranged in a randomized block design with five replications. Soil samples were taken three times/year and plant height measurements were taken annually in the summer to determine any impact of annual treatment applications on daylily plant growth. After three years, the field trial was harvested by digging up three plants/plot to take plant measurements on, such as fresh shoot and root weight (g), final plant height (cm), crown width (cm), and yield. Results show that three treatments, Dip + Indemnify, Dip + TerraClean 5.0, and Dip + 101 Starter Blend Compost, significantly reduced *Paratylenchus* spp. population levels compared to the control by 80%, 85%, and 94%, respectively. Final plant measurements show that the Dip + Indemnify and the Dip + TerraClean 5.0 treatments resulted in plants with the highest plant biomass and yield. These results give the ornamental plant industry concrete solutions to effectively manage *Paratylenchus* spp. in ornamental plant production fields.

## Nitrogen and biomass changes in tomato under CO_2_ and nematode stress

Hughes, Kody, S. Mishra, G. Keating and P. M. DiGennaro

Department of Entomology and Nematology, University of Florida, Gainesville, FL 32611

### Abstract

Rising global CO_2_ levels are expected to impact the future of agriculture. High CO_2_ growth conditions can result in higher biomass of some plants, but this can also decrease the nutritional value of the crop as the carbon-to-nitrogen ratio shifts. Organisms which rely on these plants may then be forced to consume more biomass to meet nutritional requirements. This is not only true for humans, but also for plant pests, which can result in even higher crop losses worldwide. Understanding how abiotic stressors (high CO_2_ levels) and biotic stressors (plant pests) interact is vital to preparing management strategies to mitigate potential decreases in yield and quality under projected increasing global CO_2_ levels. Here we employed root-knot nematode (RKN) due to its agricultural significance, ubiquity, and wide host range in a tomato pathosystem. We analyze nitrogen and biomass shifts and gall ratings under ambient and elevated CO_2_ for both control and RNK-infected plants. We also quantifed nitrogen assimilation rates by fertilizing the plants while under these conditions with heavy labeled nitrogen, and we examined both 15N/14N and C/N ratios with an Isotope Ratio Mass Spectrometer (IRMS) and an elemental analyzer. Comparisons of heavy labeled nitrogen isotope ratios and characterization of assimilation rates, nematode proliferation, and weight ratios yielded novel insights into the potential impacts to crop yield and quality as our climate changes over the next century.

## Cyst-forming nematodes in Costa Rica: species, variability, and biology

Humphreys-Pereira, Danny, L. Núñez-Rodríguez, R. Sandoval-Ruiz and L. Flores-Chaves

Laboratory of Nematology-CIPROC, Agronomy School, University of Costa Rica, San José, Costa Rica, 2060

### Abstract

Only two cyst-forming nematode species have been identified in Costa Rica, the pale potato cyst nematode, *Globodera pallida,* and the clover cyst nematode, *Heterodera trifolii*. The species, *G. pallida* was found in 2005 and officially described in 2009, in the northern region of Cartago, the main potato-producing area in the country. Recently, we identified this species in Zarcero, Alajuela, the second-largest potato area. This nematode has become a threat to the potato industry in Costa Rica, enhanced by its wide distribution in the potato seed production area. The sequence analyses of the ITS and the *cytb* gene of 15 populations showed a single haplotype of *G. pallida* in Costa Rica. A unique haplotype suggests a single introduction in the country and spread with contaminated material to the rest of the potato-producing areas. Furthermore, the *cytb* haplotype found in Costa Rica is similar to the northern Peru populations, which are recognized to reproduce on resistant potato materials. In contrast, *G. pallida* populations from North America, Japan, and Europe have their origin in southern Peru. The population dynamics and life cycle of *G. pallida* were studied in two plots (each with an area of approx. 1000 m^2^) in a commercial potato farm in La Pastora, Cartago with a record of nematode damage. Five individual plants per plot were collected every week, starting the second week up to the eleventh week after planting. Roots from each individual plant were stained and nematode life stages were quantified under a microscope. The highest density of J2 was observed at 28 days after planting (DAP) in both plots (2152 and 1698 J2/10g of roots), whereas females showed the highest density at 49 DAP with 694 females/10 g in plot 1, and at 56 DAP with 212 females/10 g in plot 2. Six populations of *Heterodera* from two weeds (*Rumex obtusifolius* and *Trifolium repens*) associated mostly with potato fields from different localities in Costa Rica were characterized using three DNA markers (ITS, D2-D3, and *cox1*) and identified as *H. trifolii*. This was the first official description of a *Heterodera* species in the country. The pathogenicity assays showed that these two weeds are susceptible and there are differences in the nematode reproduction factor. In contrast, the nematode did not reproduce on table beet. The life cycle of two *H. trifolii* populations was studied on the same hosts, the first cyst was observed at 35 days after inoculation (DAI) in *R. obtusifolius* and at 43 DAI in *T. repens.* Caution must be taken with *H. trifolii* because it is well known its damage on ornamental plants in other countries.

## Advancing soybean nematode, sudden death syndrome, frogeye leaf spot and target spot management employing a novel seed-applied technology

Ireland, D. S. and A. Simon Simmons

Syngenta Crop Protection – Seedcare

### Abstract

Soybean Cyst Nematode (SCN) is estimated as the number one pathogen in US soybean. Agricultural economists estimate most years the US soybean grower loses more to SCN than the next five soybean pathogens added together. One of the most recent additions to a comprehensive SCN management program is using a seed-applied nematicide (SAN). In combination with all other management tools, a SAN offers additional protection and potentially reduces the heavy reliance on single genetic SCN resistance sources. Since healthy root development is vital to establishing the most stable yield potential, SANs have been one of the most anticipated and rapidly adopted new seed-applied technologies offered in recent years. Not only has TYMIRIUM™ technology has been shown to reduce SCN damage as measured through SCN cysts/gram of root compared to current commercial standards, but also this new molecule reduces the fecundity of remaining SCN as measured by reduced egg count/cyst in remaining cysts compared to same commercial standards. Under moderate to heavy SCN field pressure TYMIRIUM™ technology (0.075 mga/seed) seed treatment in soybean outperformed FLPM (0.075 mga) by an average of +2.3 bu/A driven by a 71% win record (n=45; 2020–2022). Under the same conditions TYMIRIUM technology (0.075 mga) outperformed ABA (0.15 mga) by an average of +2.0 bu/A with a 75 percent win record (n=101; 2015–2022). Under moderate to heavy Sudden Death Syndrome infection TYMIRIUM technology (0.075 mga) outperformed FLPM (0.15 mga) by an average of +4.3 bu/A leading to a 86% win record (n=29; 2018–2022). TYMIRIUM technology also statistically reduces early season Septoria Brown Spot, Frogeye Leaf Spot and Target Spot when compared to Check treatment. When registered, TYMIRIUM technology will deliver a new level of soybean protection performance across multiple important soybean pathogens.

## Root penetration and reproductive abilities of root-lesion nematode, *Pratylenchus penetrans* in cover crops

John, Matthew, A. Plaisance and G. Yan

North Dakota State University, Department of Plant Pathology, Fargo, ND, 58108

### Abstract

Root-lesion nematode, *Pratylenchus penetrans* is an important migratory endo-parasitic nematode pest, causing significant economic and crop loss. The use of nematicides can be detrimental to other beneficial organisms and the environment. The demand for alternative management strategies has increased because of the deregistration of some synthetic chemical pesticides. Cover crops that are nonhosts, poor hosts, and/or have nematicidal activities may assist in managing *P. penetrans* in infested fields. Greenhouse experiments were conducted to screen 11 entries of cover crops, seed meals, and controls for their hosting and population reduction abilities to *P. penetrans* under controlled conditions. Experiments were arranged in a completely randomized design with five replications for each treatment and control. The host status of crops was categorized based on reproductive factor (Rf = final population density/initial population density). Alfalfa (Vernema) and potato (Castle Russet) showed a poor hosting ability for *P. penetrans* reducing the initial nematode population by 31% and 82%, respectively. Pacific Gold seed meal alone without plants reduced the nematode initial population by 88% but it was not significantly different from the non-planted control. Mustard seeds of Pacific Gold and IdaGold, litchi tomato and winter rye (Dacold) increased the nematode population showing good or excellent hosting abilities with Rf ranging from 2.3 to 4.3. A second trial is ongoing to validate the host suitability of the tested crops. Additionally, two greenhouse trials were conducted to ascertain the root penetration ability of *P. penetrans*. Alfalfa (FSG 527) and annual ryegrass (Tetilia) showing the highest population reproduction from our previous trials were selected, while potato (Red Norland) and Faba bean (Petite) identified as good hosts were included for comparison to investigate the root penetration at different time points (20, 40, and 80 days after planting) using root staining with red food color dye. Alfalfa (FSG 527) which was previously and consistently identified as a poor host, had no *P. penetrans* penetrated in the roots examined at all three time points. Annual ryegrass (Tetilia) that greatly reduced the initial population was found to have *P. penetrans* penetrated inside roots at the last harvest time. Faba bean (Petite) continued to increase the nematode population in the soil at different time points but the root penetration of *P. penetrans* was only observed at 80 days after planting. The susceptible potato (Red Norland) allowed *P. penetrans* to penetrate the plant roots at all the time points in all the trials. The cover crops with poor hosting ability and no penetration of the plant roots identified in this study have a great potential to be utilized in infested fields for managing *P. penetrans* to minimize yield loss.

## Screening commercial soybean cultivars for resistance to soybean cyst nematode

Johnson, Conner^1^, L. Lindsey^2^ and H. Lopez-Nicora^1^

^1^ The Ohio State University, Dept. of Plant Pathology, Columbus, OH 43210

^2^The Ohio State University, Dept. of Horticulture and Crop Science, Columbus, OH 43210

### Abstract

*Heterodera glycines,* commonly known as soybean cyst nematode (SCN), is the leading cause of yield loss for soybean in North America. Most commercial varieties derive their SCN resistance from cultivar PI 88788. Since the introduction of this resistance source, these varieties have been grown continuously throughout the Midwest, putting great pressure on SCN populations to adapt. In Ohio, more than 60% of fields with high SCN levels are HG type 2-, which is defined by its ability to reproduce on PI 88788 at least 10% as effectively as it reproduces on susceptible cultivars. While most commercial varieties are labeled “resistant to SCN,” this evolving situation requires study of how commercially available varieties perform against adapted SCN populations which are now found throughout the state. This study screened 128 commercial soybean cultivars against SCN populations collected from Ohio fields. Three replicates of each cultivar were inoculated with an HG type 2-population at planting. Cultivars Lee 74 and Williams 82 were used as susceptible controls. Plants were grown for 30 days in the greenhouse, then females (cysts) were dislodged and collected from the roots. The average number of females collected from each variety was divided by the average number collected from the susceptible cultivars planted in the same test to determine the female index (FI) for each variety. Varieties with less than 10% FI were categorized as “resistant,” 10%–30% as “moderately resistant,” 30%–60% as “moderately susceptible,” and greater than 60% as “susceptible.” Out of 128 varieties screened, only 12% were classified as “resistant,” 31% as “moderately resistant,” 22% as “moderately susceptible,” and 35% as “susceptible.” This specific SCN population is highly virulent to PI 88788, so farmers planting in fields with similar pathotypes can expect significant yield loss. Based on these results, recommendations to farmers include rotating with different resistance sources and non-host crops, and routinely monitoring SCN levels and population types. For breeders, this highlights the urgent need for new varieties with different sources of SCN resistance.

## Microbiome diversity associated with Beech Leaf Disease in American Beech

Kantor, Mihail^1^, A. Miles^2^, S. Crandall^1^ and Cristina, Rosa^1^

^1^Department of Plant Pathology and Environmental Microbiology, Pennsylvania State University, PA 16802

^2^Intercollege Graduate Degree Program in Plant Biology, Pennsylvania State University, PA 16802

### Abstract

Beech leaf disease (BLD) represents one of the greatest threats to the ecology of hardwood forests in the Northeastern U.S. The causal agent of BLD is a foliar nematode, *Litylenchus crenatae* spp. *mccannii*, also known as the North American beech leaf nematode. To date, there is limited information available on the BLD microbiome. This microbiome study is the first to analyze and compare the differences between the banded and non-banded leaf tissue, as well as to compare them to the uninfected leaves. For this study, three sites from the Allegheny National Forest were chosen. Microbiome data were collected from 18 leaves found in two infected trees and one naïve tree (three leaves per tree). Bud samples from symptomatic and asymptomatic trees were also analyzed. Chlorophyll measurements from all samples were recorded. Data collected from symptomatic and asymptomatic leaf tissues suggests a difference in the bacterial and fungal communities. The microbial communities were evaluated based on their relative abundance at the genera-level for both bud and leaf samples. The top five bacteria genera for banded leaf tissue were *Methylosinus* spp., *Hymenobacter* spp., *Sphringomonas* spp., *Beijerinckia* spp., and *Spirosoma* spp.; for non-banded tissue were *Methylosinus* spp., *Hymenobacter* spp., *Sphringomonas* spp., *Beijerinckia* spp., and *Wolbachia* spp.; for healthy tissue from asymptomatic trees were *Methylosinus* spp., *Sphringomonas* spp., *Terriglobus* spp., and *Beijerinckia* spp. The top five fungi genera for banded tissue were *Knufia* spp. *Taphrina* spp., *Ramularia* spp., *Cladophialophora* spp. and *Cystocoleus* spp.; for non-banded tissue were *Cladophialophora* spp., *Knufia* spp., *Ramularia* spp., *Taphrina* spp., and *Peltula* spp.; and for healthy tissue were *Opegrapha* spp., *Peltula* spp., *Cladophialophora* spp., *Ramularia* spp., and *Knufia* spp. The top five bacterial genera for the asymptomatic tree buds were *Terriglobus* spp., *Sphingomonas* spp., *Methylosinus* spp., *Kaistobacter* spp., and *Bdellovibrio* spp.; for the symptomatic tree buds were *Methylosinus* spp., *Hymenobacter* spp., *Terriglobus* spp., *Sphingomonas* spp., and *Spirosoma* spp. The top five fungal genera for the asymptomatic tree buds were *Cystocoleus* spp., *Cladophialophora* spp., *Opegrapha* spp., *Cyphellophora* spp., and *Briancoppinsia* spp.; for the symptomatic tree buds were *Knufia* spp., *Taphrina* spp., *Dimidiographa* spp., *Cystocoleus* spp., and *Cyphellophora* spp. The bacteria in genus *Erwinia* spp., *Massilia* spp., *Methylobacterium* spp., *Pseudomonas* spp., *Serratia* spp., *Spirosoma* spp., and *Wolbachia* spp., along with fungi in genus *Knufia* spp. and *Taphrina* spp. identified in this study will be considered for further studies. In conclusion, morphological and metabolic differences exist between the symptomatic and asymptomatic leaves which could directly or indirectly affect the microbial communities. This study provides very important data on the different microbial and fungal communities present in symptomatic, asymptomatic, and naive leaves. It represents the first microbiome community comparison between the banded and non-banded portions of the same leaf.

## Project nematoda

Kantor, Mihail^1^ and J. D. Eisenback^2^

^1^Department of Plant Pathology and Environmental Microbiology, Pennsylvania State University, University Park, PA 16802

^2^School of Plant and Environmental Sciences, Virginia Tech, Blacksburg, VA 24061

### Abstract

Phylum Nematoda comprises an abundant group of animals that live in diverse habitats including parasites of animals, invertebrates, and plants, and as free-living forms that occur in freshwater, marine habitats, and terrestrial environments. Beginning with the very first descriptions of nematodes by Carl Linneaus in 1758, to date, more than 25,000 nematode species have been described. Since the original descriptions of many nematodes are only available in-print and sometimes in foreign and obscure places, significant effort is required to collect all of the species from a particular genus or other taxonomic group. Digital collections of these descriptions preserve them and make them readily accessible to interested users. Project Nematoda attempts to digitize and preserve the original descriptions of all species of nematodes from every habitat. The collection began by digitizing the reprint collections of Drs. Eisenback, Hirschmann, Miller, Sasser, and Triantaphyllou in the late 1980s and was greatly accelerated when the portable document format (PDF) was developed by Adobe^®^ in 1993. In 2017, the Society of Nematologists supported the creation of a Zotero^®^ library to host the collection of PDFs. Currently, Project Nematoda includes over 7,500 nematodes species descriptions. Searching, exporting, and contributing to the Project Nematoda with new descriptions of nematodes is very user friendly. This workshop will demonstrate how to navigate and use Project Nematoda and how to add species descriptions to make the project more complete.

## Unearthing the hidden world: exploring the endosymbionts in nematode communities through soil sampling

Kaur, Amandeep and A. M. V. Brown

Department of Biological Sciences, Texas Tech University, Lubbock, Texas

### Abstract

Recent studies of economically important plant-parasitic nematodes (PPNs) have revealed the presence of bacterial partners such that together the PPN and microbes may act as a holobiont. PPNs are a significant threat to global agriculture, causing billions of dollars in crop losses each year. This study aims to investigate the endosymbionts hidden within nematode communities by exploring the distribution and diversity of the endosymbiont *Wolbachia* in nematodes from soil samples. *Wolbachia* is a bacterial endosymbiont that infects a wide range of arthropods and a narrow range of nematodes, having major impacts on the biology and ecology of its hosts. Strains of *Wolbachia* can cause reproductive manipulations – male killing, feminization, parthenogenesis, and cytoplasmic incompatibility in its arthropod hosts and can be obligate nutritional mutualists that are essential for survival in some arthropods and filarial nematode hosts. *Wolbachia* has been used as a biological control agent in mosquitoes to control their populations through cytoplasmic incompatibility and *Wolbachia*-targeting antibiotics can control filarial nematode disease. These outcomes raise interest in finding and characterizing PPN *Wolbachia* strains for potential biological control approaches. In the current study, broad sampling was conducted to collect root and soil samples from different locations in 21 US states covering ~300 sampling sites from agricultural fields, grasslands, and forests. The soil samples were processed to extract the nematode communities through the Baermann funnel technique. Novel PCR assays were developed for PPN-type *Wolbachia* by designing specific primers using Geneious Prime with alignments to PPN-type strains recently discovered or extracted from database mining. Primer specificity was further confirmed by Sanger sequencing of the PCR products followed by phylogenetic analysis. Sequences clustered with known PPN-type *Wolbachia*, but also formed numerous other deep branches within genus *Wolbachia*, basal to all other known strains. Screening with these PCR primers showed that approximately 45% of the nematode communities processed so far tested positive for the presence of *Wolbachia*, or these *Wolbachia*-like deep branching strains. This high prevalence was unexpected, but suggests a hidden world of endosymbionts yet to be discovered. Future work will focus on whole genome sequencing of these *Wolbachia*-hosting communities and from polished genomes also examined signatures of selection across genes and pathways to infer the potential roles of these mysterious endosymbionts.

## Greenhouse evaluation of root knot nematode resistant pepper lines against meloidogyne floridensis

Khanal, Churamani^1^, W. Rutter^2^, M. Alam^1^, I. Alarcon^1^ and D. Harshman^1^

^1^Clemson University, Dept. of Plant and Environmental Sciences, Clemson, SC 29634

^2^USDA-ARS, U.S. Vegetable Laboratory, Charleston, SC 29449

### Abstract

Six pepper (*Capsicum annum*) lines (‘Carolina Wonder’, ‘Charleston Belle’, HDA149, HDA330, PM217, and PM687) carrying different root-knot nematode (RKN, *Meloidogyne* spp.) resistance gene(s) were screened in repeated greenhouse and growth chamber environments against *Meloidogyne floridensis*. The USDA line PA136, considered a universal RKN susceptible line, was also included as a control along with an F1 hybrid of ‘Carolina Wonder’ X PA136. For each experiment, six-week-old pepper lines with four replications of each genotype were grown in six-inch plastic pots containing 1.5 kg steam-sterilized soil and were inoculated with 10,000 eggs of *M. floridensis*. Across all pepper lines, the number of eggs per gram of dry roots two months after inoculation ranged from 24 to 91,996. Interestingly, the susceptible control PA136, showed significantly less *M. floridensis* reproduction and galling compared to the RKN resistant lines Carolina Wonder, Charleston Belle, and HDA149. While the RKN resistant lines HDA330 and PM687 produced a statistically intermediate reproduction. The nematode reproduction on the F1 ‘Carolina Wonder’ X PA136 and PM217 were statistically similar to PA136. The results from these screens support previous observations from other host crops that *M. floridensis* has a very different host specificity than related clade I *Meloidogyne* species. They also suggest that line PA136 contains at least one dominant gene that confers *M. floridensis* resistance. Results from this study will be useful in *M. floridensis* resistant pepper breeding programs.

## Identification, characterization and validation of reniform nematode (*Rotylenchulus reniformis*) resistance in soybeans

Kiarie, Lucy and T. Watson

LSU AgCenter, Department of Plant Pathology and Crop Physiology, Baton Rouge, LA 70803

### Abstract

The reniform nematode (*Rotylenchulus reniformis;* RN) is among the most economically important plant-parasitic nematode associated with soybean production in the southern United States, including Louisiana. This injurious nematode establishes a permanent-feeding site (syncytium) after root penetration where the females feed semi-endoparasitically. As a result, soybean fields infested with this damaging nematode show significant yield reduction, empty pods, root decay and unthrifty growth, with above ground symptoms that include areas with uneven stunted plant growth and foliage chlorosis. Several resistant soybean genotypes have been identified for soybean cyst and root knot nematodes; however, less effort has been dedicated to identifying and evaluating host resistance to RN. Resistant soybean germplasm against RN has been identified among soybean cyst nematode resistant breeding lines originally derived from the cultivar Peking. Unfortunately, soybean genotypes classified as resistant to RN are few compared to susceptible soybean germplasms. The aim of the present research study is to screen diverse soybean plant introductions (PIs) from the USDA-ARS Soybean Germplasm collection for RN resistance and to identify additional new quantitative trait loci associated with resistance using a genome-wide association study (GWAS). A density of 1000 mixed-stage RN were used as initial population density and inoculated into sterile soil in cone-tainers planted with 367 different soybean PIs from maturity groups 4 and 5. After 11 weeks of growth in the greenhouse RN soil population densities was quantified. Results from the present GWAS will be validated using small plot field trials conducted in Louisiana. Field trials conducted in 2021 and 2022 have demonstrated a lack of RN resistance in the top commercially planted soybean varieties in Louisiana; however, planting a RN resistant soybean breeding line significantly reduced RN soil population densities and improved yield relative to commercial varieties.

## Effects of bed architecture and drip emitter spacing on nematicide effectiveness for control of root-knot nematodes in cucumber

Kidane, Selamawit^1^, J. Desaeger^2^ and A. Hajihassani^1^

^1^University of Florida, Department of Entomology and Nematology, Fort Lauderdale Research & Education Center, Davie, FL 33314

^2^University of Florida, Department of Entomology and Nematology, Gulf Coast Research and Education Center, Wimauma, FL 33598

### Abstract

Florida is a top producer of vegetables in the US. Vegetables are grown year-round on raised beds covered with polyethylene plastic mulch which helps conserve moisture and regulate soil temperature. Extensive cultivation using plastic beds increases the risks of pests and pathogens including some species of plant-parasitic nematodes. Root-knot nematodes are major pests of vegetable crops in this plasticulture system, and their management has mainly relied on chemical fumigants and non-fumigant nematicides. A field trial was set up at the Gulf Coast Research and Education Center (GCREC), Wimauma, FL in the Fall of 2022 to study the effect of bed architecture and drip emitter spacing on the efficacy of nematicides to manage root-knot nematodes. A complete factorial experiment in a split-split plot design with 5 replications was established to evaluate two non-fumigant nematicides (Velum Prime® and Vydate®), two drip emitter spacings (6′ and 12′), and two bed designs [standard (350 ft long × 2.5 ft wide) and compact bed design (350 ft long × 1.64 ft wide)]. Untreated controls were established in each block for comparison. Cucumber (*Cucumis sativus*) variety ‘Dasher II’ was directly seeded into beds (20 plants/plot). The soil at GCREC is classified as Myakka fine sand. The beds were covered in black plastic mulch and there was one drip tape per bed. Nematicides were injected using pressurized CO_2_ tanks into drip lines. The field of the study has a history of infestation with *Meloidogyne javanica*. Root gall ratings were taken from five plants per plot based on a 0–10 scale where 0 indicates no visible root galling and 10 represents 100% galled roots. Data were analyzed using R studio. Preliminary results indicated that the interaction of bed design and drip tape emitter spacing had a significant difference (*P* = 0.00738) in the total cucumber yield. Higher yield (58 marketable-size cucumbers) was obtained from standard beds. Mid-season gall ratings showed significant differences among treatments; all compact beds had lower gall ratings (0.5 – 1) than other treatment combinations and the untreated controls. End-season gall ratings also showed a significant difference between compact and standard beds. These findings show that non-fumigant nematicides combined with the compact bed design and 15 cm drip tape emitter spacings can provide good management of root-knot nematodes in raised-bed vegetable production systems.

## Control of virulent *Globodera pallida* populations using new sources of plant resistance

Kiewnick, Sebastian

Julius Kühn Institut, Federal Research Centre for Cultivated Plants, Institute for Plant Protection in Field Crops and Grassland, Messeweg 11/12, 38104 Braunschweig, Germany

### Abstract

Potato cyst nematodes (PCN), and in particular *Globodera pallida*, are a major constraint for the potato production in Europe. Due to their quarantine status in the European Union, restrictions apply once PCNs are detected in a field. In the areas of Germany and the Netherlands, where starch potatoes are intensively grown, the use of resistant potato varieties was the most efficient way to control *G. pallida*. As part of the EU regulation, only varieties with a high level of resistance (0–3% relative susceptibility) can be grown in infested fields as part of the official control program. However, intensive use of these varieties with Grp1 resistance has led to the selection of a new virulence type of *G. pallida*, which was found in the Emsland region in Germany. After the official report in 2014, authorities from The Netherlands also reported the presence of *G. pallida* populations of a new virulence type from areas with intensive starch potato production. Intensive testing of available potato varieties with resistance to a-virulent Pa2/Pa3 populations revealed that no commercial varieties are available to the farmers for control. Therefore, the selection of new sources of resistance against these virulent populations was needed. Controlling populations of this new virulence type is challenging, as they seem to have a higher biological fitness resulting in a high reproduction rates on both, resistant and susceptible cultivars. In addition, once selected for virulence, this process is not reversible even after several reproduction cycles on susceptible cultivars. Within the framework of the projects ‘PARES’ (FKZ 22016614) and ‘SERAP’ (FKZ219NR413) funded by the Fachagentur fuer Nachwachsende Rohstoffe (FNR) on behalf of the Federal Ministry of Food and Agriculture (BMEL), new sources of resistance from a wide range of accessions of wild potato species were screened in pot experiments against the well-characterized virulent population ‘Oberlangen’. To improve the phenotyping process, tissue culture plants were utilized shorten the selection process for resistant genotypes. From this screening process, over 50 genotypes were identified that carry a high level of resistance against ‘Oberlangen’ as well as the reference Pa3 population ‘Chavornay’. These selected genotypes are now included in breeding programs and investigated for the modes of action that are responsible for suppressing *G. pallida*. Based on the genotypes tested, resistance is based on similar modes of action. In addition, the level of resistance is independent from the *G. pallida* population used for testing. One main goal is to combine different modes of resistance to breed for a durable resistance in starch potatoes.

## *Meloidogyne enterolobii*, an emerging regulated quarantine nematode species in the European Union

Kiewnick, Sebastian^1^ and S. Koenig^2^

^1^Julius Kühn-Institut, Federal Research Centre for Cultivated Plants

^2^Institute for Plant Protection in Field Crops and Grassland, Institute for National and International Plant Health, Messeweg 11/12, 38104 Braunschweig, Germany

### Abstract

The root-knot nematode *Meloidogyne enterolobii* Yang & Eisenback (syn. *M. mayaguensis*) has received a lot of attention since it has been first reported on cucumber and tomatoes in two Swiss greenhouses in the year 2008. Due to the wide host range and its ability to overcome resistance against other tropical *Meloidogyne* species, *M. enterolobii* was identified as a major threat to crops worldwide. In 2009, the European Plant Protection Organization (EPPO) performed a risk analysis, which came to the conclusion that this species was recommended for regulation and placed on the EPPO A2 list in 2010. Following numerous interceptions over the past years, it was concluded that *M. enterolobii* fulfilled the conditions provided in Article 3 and Section 1 of Annex I to Regulation (EU) 2016/2031 in respect of the Union territory and therefore should be listed in Part A of Annex II to Implementing Regulation (EU) 2019/2072 as Union quarantine pest. The measures for all plants for plantings in relation to *M. enterolobii* apply therefore from 11 January 2023 onward. As for other quarantine nematode species, surveys must now be conducted at least once within a 7-year cycle by national plant protection services, which might lead to more reports in the future. It is still unknown how widespread *M. enterolobii* might be present in the EU, as there was only one new finding reported from Portugal in recent years. Although EPPO standards on detection and identification are in place, *M. enterolobii* might have been often overlooked and identified as highly virulent tropical *Meloidogyne* species, such as *M. incognita*. With the new regulation in place, several interceptions have been made, which suspect the presence of *M. enterolobii* in different commodities. In preparation for potential new introductions and or spread throughout the EU, it is critical to determine the host range, the potential to adapt to non- and poor-hosts as well as the genetic diversity of *M. enterolobii*. This will allow for the development of integrated management strategies to mitigate the potential losses and ensure sustainable production of high value crops in particular for southern Europe.

## Selectively killing parasitic nematodes via P450 bioactivation of small molecules

Knox, Jessica^1,2^, A. R. Burns^1,2^, B. Cooke^1,2^, S. R. Cammalleri^1,2^, M. Kitner^3^, J. M. P. Castelli^1,2^, E. Puumala^1^, J. Snider^2^, I. Stagljar^1,2^, L. E. Cowen^1^, I. A. Zasada^3^ and P. J. Roy^1,2,4^

^1^Department of Molecular Genetics, University of Toronto, Toronto, ON

^2^Terrence Donnelly Centre for Cellular and Biomolecular Research, University of Toronto, Toronto, ON

^3^United States Department of Agriculture – Agricultural Research Service (USDA-ARS) Horticultural Crops Research Laboratory, Corvallis, OR

^4^Department of Pharmacology and Toxicology, University of Toronto, Toronto, ON

### Abstract

Our group is interested in early stage ‘drug’ development focused on compounds that kill parasitic nematodes. We previously identified 67 small molecule scaffolds with nematode selectivity. From these nematicidal candidates, we identified several whose activity is cytochrome P450-dependent. In this presentation, I focus on one of these called Cyproside-3. We screened 846 molecules with physicochemical features similar to Cyproside-3 and identified one that we call Cyproside-3c that has potency against diverse plant parasitic nematodes, including *Meloidogyne hapla, Ditylenchus dipsaci,* and *Pratylenchus penetrans*. Through various screens that I will describe, we identified the *C. elegans* and *Meloidogyne incognita* P450s that bioactivate Cyproside-3c. We used LCMS to demonstrate that Cyproside-3c is metabolized into an electrophilic reactive product and show through heterologous assays that P450-generation of the metabolite is sufficient to kill cells. We conclude that Cyproside-3c is a promising lead molecule with the potential to control parasitic nematode infestation of crops.

## Soil biological responses at the West Virginia university long-term organic field crop/livestock systems trial

Kotcon, James

Division of Plant and Soil Sciences, West Virginia University, Morgantown, WV 26506

### Abstract

The WVU Long-Term Farming Systems Trial was initiated in 1999 in an effort to evaluate the response of field crop systems to key organic management practices. Soil quality, crop yields, pest populations and microbial activity were determined to track responses to the farming systems. Four farming systems: with and without manure, with and without livestock (adding pasture in the crop rotation) in all combinations; have been evaluated since 2000, with a potato-soybean-wheat-brassica rotation. Corn replaced potato after 2010. Plots with composted manure received 20 tons per acre prior to corn and wheat. Plots with livestock added 3 years of orchard grass/red clover pasture (ley) after the brassica crop, and were harvested for hay or grazed with sheep. Legume cover crops (rye/vetch and cowpea) were used to increase nitrogen fertility. Soil samples were collected in November each year and analyzed for soil fertility and various soil biota. Soil organic matter content increased significantly in all farming systems, but was greater in plots with manure than plots without and in unmanured plots with the ley in the rotation. Soil phosphorus and potassium showed somewhat similar trends. Earthworm populations fluctuated greatly, but were greater in manured plots than unmanured. Combining manure with the ley tends to conserve nitrogen until crop plants need it most. Population densities of plant parasitic (*Pratylenchus crenatus*, *P. penetrans*, *Xiphinema rivesi*, *Helicotylenchus* spp., *Hoplolaimus galeatus*) were variable but tended to remain low in spite of planting of susceptible crops, suggesting soil suppressiveness. Carnivorous nematodes (*Clarkus papillatus*) remained at low populations in all treatments while bacteriovore nematode densities were greater in plots with manure than without. Nematode-trapping fungi increased over the trial, but did not differ significantly among treatments. After 20+ years, these organic farming systems have generated significant differences in soil fertility, but few differences in nematode populations. Work to determine the mechanism of nematode suppressiveness is underway.

## Reproduction of *Pratyenchus scribneri* and *P. penetrans* on seven cultivars of industrial hemp

Kotcon, James and N. Alvarado

Division of Plant and Soil Sciences, West Virginia University, Morgantown, WV 26506

### Abstract

Cultivation of industrial hemp (*Cannabis sativa*) was legalized in the Farm bill in 2018. Little information is available regarding the susceptibility to common plant-parasitic nematodes, and no previous studies from the US of root lesion nematodes have been identified. We evaluated reproduction of *Pratylenchus scribneri* and *P. penetrans* on seven grain/fiber cultivars of *C. sativa* (Bialobrzeskie, Fedora 19, Felina 32, Futura 75, Orion, Tygra, USO-31) and soybean cv. Viking 1202N (positive control). Nematodes were extracted from infected corn and soybean roots, respectively, from greenhouse cultures using Baermann funnels for 4 days. In the first experiment, 2-to-3-week-old seedlings were transplanted into 300 g of a sterile sand-soil mix and three plants each were inoculated with 0 or 40 *P. scribneri*. Plants were maintained on a lab bench at 22C under artificial light with an 18-hour photoperiod. Plants were harvested after 39 days, and nematodes were extracted from roots as above. In the second experiment, three plants each were inoculated with 180 *P. scribneri*, 180 *P. penetrans,* or were uninoculated. Plants were harvested after 36 days, and nematodes were extracted from roots. In the first experiment, soybean cv. Viking 1202N and *C. sativa* cvs. Futura and USO-31 had more *P. scribneri* per plant than Tygra, however, *P. scribneri* population differences were not significant in the second experiment. Soybean produced more *P. penetrans* than all hemp cvs. except Tygra. Tygra and Felina had greater populations of *P. penetrans* than Orion, Bialobrzeskie, or USO-31, while Futura was intermediate. *P. penetrans-*inoculated soybean had reduced root weight compared to controls, but no root or shoot weight reductions were evident in any hemp cultivar at these inoculum levels for either nematode. Because lesion nematodes are widespread in hemp-producing areas, field studies to assess yield loss at a range of population densities may be warranted, and screening of more cultivars for resistance could be useful for growers with infested fields.

## Discovery and initial analysis of a novel rhabdovirus associated with the Idaho population of the potato cyst nematode *Globodera pallida*

Kud, Joanna^1,2^, J. Dahan^1^, G. E. Orellana^1^, L. M. Dandurand^1^ and A. V. Karasev^1^

^1^University of Idaho, Dept. of Entomology, Plant Pathology and Nematology, Moscow, ID

^2^Department of Entomology and Plant Pathology, University of Arkansas, Fayetteville, AR

### Abstract

*Globodera pallida*, a potato cyst nematode (PCN), is a globally regulated plant parasitic nematode that may reduce tuber yields up to 80% in infested potato (*Solanum tuberosum*) fields if left unmanaged. Since its detection in Idaho, in 2006, PCN is under strict quarantine and eradication programs in the United States. Restrictions on use of chemical pesticides have put studies on alternative methods of nematode control in the spotlight. Many examples show that viruses are viable as biological control agents for limiting the impact phytopathogens, such as fungi, bacteria, insects, and even other viruses. A novel rhabdovirus genome was assembled from the transcriptomics dataset from the Idaho PCN populations. The presence of this virus, named potato cyst nematode rhabdovirus (PcRV), was validated and confirmed for the different life stages of the *G. pallida* populations maintained in the quarantine greenhouse in Idaho (USA), and the virus whole genome was re-sequenced using Sanger methodology. Another isolate of PcRV was identified among the publicly available transcriptomics outputs generated from the Scottish populations of *G. pallida*, with the genome sequence almost identical to the Idaho isolate of PcRV. Two other *Globodera* spp. reproducing on potato and reported in the US, *G. rostochiensis* and *G. ellingtonae*, tested negative for PcRV presence. PcRV has a 13,604-bp long, single-stranded RNA genome encoding five open reading frames and is the most closely related to soybean cyst nematode rhabdovirus (ScRV). Based on their similar genome organizations, the phylogeny of their RNA-dependent RNA polymerase domains (L gene), and relatively high identity levels in their protein products, we propose to create a new genus with a tentative name *Gammanemrhavirus*, comprising two virus species, representing PcRV and ScRV, respectively.

## Biological control of soybean cyst nematode by beneficial fungi

Kunwar, Vijay, M. C. Aime and L. Zhang

Department of Botany and Plant Pathology, Purdue University, West Lafayette, IN 47907

### Abstract

Soybean cyst nematode (SCN) is the most devastating pathogen of soybean in the US, causing annual yield losses estimated at $1.5 billion. However, the frequently used soybean varieties with resistance from the breeding line PI 88788 become less effective in controlling this pest due to the emergence of widespread virulent SCN populations that have been able to overcome host resistance. One increasing deployed strategy to combat the rise in virulent SCN has been growing soybean seeds treated with biologicals according to two surveys conducted in 2015 and 2020 by the SCN Coalition. However, one issue with commercially available seed treatment products using bacterial biologicals is the lack of consistency in controlling SCN. Our goal is to develop beneficial soil-dwelling fungi as biologicals to effectively and consistently control SCN. Prior collaborative efforts isolated and assayed hundreds of fungal isolates from a global distribution of cyst nematode samples, identifying 63 fungal species with antagonistic potential against cyst nematodes. Here we further screened and identified those fungal species that are specifically antagonistic to SCN. We have developed a fast *in vitro* screening pipeline to evaluate the biocontrol potential of fungal isolates by assessing egg parasitism, cyst parasitism, egg viability, hatch inhibition and second-stage juvenile mortality. At least six fungal isolates have shown promising results in controlling SCN. We have been conducting more assessment through *in planta* assays. We expect to provide growers with new methods for sustainable control of SCN and reduce soybean yield losses to this devastating pest.

## The effects of nickel on *Meloidogyne enterolobii* embryogenesis and egg hatching

Kuo, Hao-yu and J. Yang

Department of Plant Pathology and Microbiology, National Taiwan University, No. 1 Roosevelt Rd., Sec. 4, Da-an Dist., Taipei City, 10617, Taiwan

### Abstract

Root-knot nematodes (*Meloidogyne* spp., RKN) have a broad host range and are distributed worldwide. These nematodes induce galls on host roots, reduce plant growth, and cause approximately US$ 1.7 trillion in global economic losses annually. The second-stage juvenile (J2) is the infecting stage. However, the embryogenesis process and the egg-hatching behavior of RKN before the J2 stage are essential for later successful host parasitism. The eggshell of the RKN egg is constructed with multiple layers and is known to protect the embryo from being harmed by environmental stresses. Previous studies have shown that the stylet thrusting and enzymes such as lipase and chitinase are critical in these processes. Prior studies in our laboratory revealed that environmental nickel shortened the embryonic development period but delayed the hatching of *M. enterolobii* eggs. The result indicated that *M. enterolobii* might have a unique heavy metal reaction mechanism never reported before. Therefore, this study aimed to investigate the effect of nickel on the *M. enterolobii* hatching mechanism. Physiological experiments were conducted within environments containing nickel concentrations (100, 2000, and 3000 ppm). The ratio of abnormal eggs to unhatched eggs significantly increased, and the hatching rate decreased by 63.3% at 2,000 ppm and 3,000 ppm, respectively. As the concentration increased, the time needed for the hatching rate to reach 50% was longer. There was an acceleration trend from the multi-cell stage to the first-stage juvenile (J1) stage at 100 ppm. Additionally, the rate of abnormal embryonic development was as high as 71.1% and 78.8% at concentrations of 2,000 ppm and 3,000 ppm, respectively. The stylet thrusting frequency of unhatched J2 decreased by 14.4% at 2,000 ppm and 46.5% at 3,000 ppm. However, nickel didn’t affect the unhatched J2 morphology, such as the stylet length and the thickness of the cuticle or muscle fiber. Future studies of the eggshell structure and the lipase and chitinase activity of unhatched J2 will reveal the influence of nickel on eggshell permeability. Eventually, transcriptome analysis and qRT-PCR will be used to evaluate the overall effect of nickel on RKN embryogenesis and to reveal possible mechanisms for regulating egg hatching of *M. enterolobii*.

## Field efficacy of nematicidal compounds against the spiral nematode, *Helicotylenchus microlobus* on turfgrass in Korea

Kwon, Chanki^1^, A. O. Mwamula^1,2,3^, O. G. Kwon^2^ and D. W. Lee^1,2,3^

^1^Department of Plant Protection and Quarantine, Kyungpook National University, Daegu, 41566, Republic of Korea

^2^Department of Entomology, Ecological Science, Kyungpook National University, Sangju, 37224, Republic of Korea

^3^Research Institute of Invertebrate Vector, Kyungpook National University, Sangju 37224, Republic of Korea

### Abstract

Several plant-parasitic nematodes (PPNs) are major pests on turfgrass worldwide. The spiral nematode, *Helicotylenchus microlobus*, often misidentified as *H. pseudorobustus* in turfgrass growing areas, is one of the most damaging PPNs of economic importance on turfgrass health. Control and management of PPNs on turfgrass is still at the basic level, and reliable control strategies are much needed to safeguard golf courses. In this study, field experiments were conducted to evaluate the efficacy of two non-fumigant chemical nematicides (imicyafos and fluopyram) and the novel cyclobutrifluram against the spiral nematode. The three nematicides were applied in two different formulations (granules and concentrates). Imicyafos, fluopyram and cyclobutrifluram granule formulations were applied at a rate of 6, 10 and 1 g/m^2^, respectively. Soluble concentrate of imicyafos (30%) and suspension concentrate of cyclobutrifluram (50%) and fluopyram (34.8%) were applied using drench method at a rate of 2.5, 2.78, and 1.25 ml in 10 L/m^2^, respectively. Granular application of imicyafos greatly suppressed spiral nematode population compared to the untreated control, the suppression being 95 and 87% after 30 and 60 days, respectively. Treatment with the soluble concentrate formulation of imicyafos yielded suppression rates of 72 and 73% after 30 and 60 days, respectively. The two fluopyram formulations (suspension concentrate and granules) showed limited effect on nematode population after 30 days (suppression rate of 0–18%). However, significant nematode-population suppression of 73% was evident after 60 days. Similarly, the granule and concentrate formulations of cyclobutrifluram showed moderate suppression effects of 16% (granular treatment) to 44% (concentrate treatment) after 30 days. However, significant suppression effects of 82% (granular treatment) and 70% (concentrate treatment) were recorded after 60 days. Therefore, cyclobutrifluram potentially has a sustained population suppression effect in the soil after application. The results from this study suggest that the non-fumigant nematicide imicyafos, and cyclobutrifluram, a novel succinate dehydrogenase inhibitor, have a potential to serve as an alternative to fumigants in the management of turfgrass parasitic nematodes.

## Genome scan for selection signatures reveals candidate virulence genes of *Heterodera glycines* on resistant soybean

Kwon, Khee–Man^1^, J. P. G. Viana^2^, K. K. O. Walden^3^, M. E. Hudson^2^ and M. G. Mitchum^1^

^1^University of Georgia, Dept. of Plant Pathology and Institute of Plant Breeding, Genetics and Genomics, Athens, GA 30602

^2^University of Illinois, Dept. of Crop Sciences, Urbana, IL 61801

^3^University of Illinois, Carver Biotechnology Center, Urbana, IL 61801

### Abstract

The soybean cyst nematode (SCN, *Heterodera glycines*) is a major pest of soybean primarily managed through resistant cultivars. However, sexually reproducing SCN populations evolve virulence as their gene frequencies change due to uniform selection pressures driven by repeated monoculture of the same resistance sources. Resistance to SCN HG type 0 (Race 3) in plant introduction (PI) 548402 (Peking) and PI 437654 requires an epistatic interaction between *Rhg1* (*rhg1-a*) and *Rhg4*, mediated by an α-SNAP and a serine hydroxymethyltransferase, respectively. SCN populations that can overcome this type of resistance likely contain higher frequencies of individuals with virulence genes, although their identity remains largely unknown. To identify the candidate genomic regions, genes, and single nucleotide polymorphisms (SNPs) that may have evolved under selection for SCN virulence, we conducted whole genome resequencing of pools of individuals (Pool-Seq) for two pairs of avirulent and virulent SCN populations adapted to the *rhg1-a*/*Rhg4*-mediated resistance independently derived from Peking and PI 437654. We scanned the genome using population differentiation (*F*_ST_) - and principal component analysis (PCA)-based approaches to detect signatures of selection. Our Pool-Seq results identified clear signatures of selection in five genomic regions spanning four chromosomes. Some candidate regions were detected in both pairs of SCN populations, while others were unique genomic regions under selection from each pair. By finding overlapping SNPs discovered from multiple outlier detection methods, a total of 316 SNPs from both population pairs met our thresholds for significantly overly differentiated SNPs. These SNP-harboring candidate genes are being tested for their correlation to virulence followed by molecular functional studies to 1) provide new insights into the genetic mechanisms that enable SCN to overcome soybean resistance and 2) inform the development of molecular markers for rapidly screening the virulence profile of a SCN-infested field.

## Population dynamics of plant-parasitic nematodes affecting golf course turfgrass in South Florida

Larkin, Jacob^1^, W. Crow^2^, M. Schiavon^3^ and A. Hajihassani^1^

^1^University of Florida, Ft. Lauderdale Research and Education Center, Dept. of Entomology and Nematology, Davie, FL 33314

^2^University of Florida, Dept. of Entomology and Nematology, Gainesville, FL 32611

^3^University of Florida, Ft. Lauderdale Research and Education Center, Dept. of Environmental Horticulture / Turfgrass Science, Davie, FL 33314

### Abstract

Golf is a major contributor to Florida’s economy and provides enjoyment for visitors and residents alike. In over 1,000 golf courses in Florida, it has been estimated that they have an annual revenue of $4 billion and employ more than 70,000 people. As a result, there is a lot of vested interest in the maintenance and care of golf courses. A major contributor to golf course damage is plant-parasitic nematodes (PPN) that feed on turfgrass roots and negatively affect many aspects of an optimal course such as evenness of the playing field. PPN are particularly troublesome in Florida due to the favorable environmental conditions and sandy soil that facilitates a greater ease of movement through the large soil pores. Despite their problematic nature in golf courses, PPN are still largely misrepresented in pest management research, especially in the South Florida region. The objective of this study was to analyze the population dynamics of three groups of PPN, including sting (*Belonolaimus longicaudatus*), lance (*Hoplolaimus* spp.), and root-knot (*Meloidogyne* spp.) nematodes in three active golf courses, and one golf course-like turf research plot in Southern Florida. These nematode species were selected as they are considered the most damaging to turfgrass systems and thus the most compelling to monitor for management purposes in Florida. The three golf courses were selected to be sampled since the superintendents of these courses noted recurring nematode damage on the play area of their courses. The golf course-like turf research plot has also exhibited prolific nematode damage and is considered a control for the study as we can personally oversee the factors that go into the turf’s management. Three different areas within the playfields of the locations were selected in September 2022 that were particularly symptomatic for nematode damage and/or have had previous nematode reports. Beginning in October 2022, we collected soil samples from each of the locations once per month. After collection, nematodes were extracted from 100 cm^3^ of soil using a standard sugar flotation method, and nematode abundance and diversity were recorded. Preliminary results indicated a slight decrease in sting, lance, and root-knot population densities between October and November 2022 then an increase beginning in December 2022 through February 2023. A clear pattern of PPN population fluctuations will become available when our study is complete in October 2023. In addition, the population fluctuations will be connected to several edaphic, spatial, and environmental variables to potentially elucidate additional population change prediction variables. This information could help superintendents apply control measures preventatively per season rather than the current model of reactionary control.

## Characterizing indicator microbial communities associated with the northern root-knot nematode (*Meloidogyne hapla*) occurrence and soil health conditions

Lartey, Isaac^1^, G. M. N. Benucci^2^, T. Marsh^3^, G. Bonito^2^ and H. Melakeberhan^1^

^1^Agricultural Nematology Laboratory, Department of Horticulture

^2^Department of Plant, Soil, and Microbial Sciences

^3^Department of Microbiology and Molecular Genetics, Michigan State University, East Lansing, MI 48824, USA

### Abstract

*Meloidogyne hapla* is among the soil-dwelling plant-parasitic nematode with known parasitic variability (PV) where morphologically and genetically indistinguishable populations reproduce at different rates in the same host. While populations that come from mineral soils had higher PV than those from muck soils, why such differences in PV exist between nematode populations from the two soil groups and what specific soil conditions elicit these differences remains unknown. This is particularly significant in terms of soil health and biophysicochemical conditions because *M. hapla* exists in soils ranging from healthy to degraded conditions. More recently, it was shown that *M. hapla* was present in mineral and muck soils with disturbed and/or degraded conditions, as defined by the soil food web (SFW) model. Further PV tests of populations isolated from the disturbed and degraded soils showed a cluster of high, medium and low PV. The high and medium PV populations came from degraded mineral soils. In addition to suggesting linkages between *M. hapla* PV and soil health conditions, the results raised a question of what biophysicochemical changes, soil microbiome in particular, may be associated with the soils where the *M. hapla* populations were isolated. The objective of this study was to identify indicator-microbes associated with *M. hapla* occurrence and the soil health conditions as described by the SFW model. Our working hypothesis was that there are microbial indicators associated with *M. hapla* or soil health conditions. We characterized the bacterial and fungal communities using high-throughput sequencing of 16S and Internal Transcribed Spacer rDNA in the same soils harboring the high-, medium- and low-PV populations of *M. hapla*. The bacterial and fungal community abundance varied by soil group, soil health conditions, and/or *M. hapla* occurrence. However, 25 bacteria operational taxonomic units (OTUs) were distinctively associated with the high- and medium-PV populations of *M. hapla* from fields with degraded mineral soils compared to the low-PV populations from mineral or muck soils with other soil health conditions. Out of 1,065 soil health indicator bacterial OTUs, 73.9% were indicator of the maturing (best case for nutrient cycling and agroecosystem fitness), 8.4% of the disturbed, 0.4% of the degraded soil condition, and no indicators were common to the three categories. The hypothesis is supported. These findings provide a foundation for understanding the environment where *M. hapla* exists and the conditions associated with PV.

## Single worm long read sequencing reveals genome diversity in free-living nematodes

Lee, Yi-Chien^1,2,3^, H. -H. Lee^1^, H. -M. Ke^4^, Y. -C. Liu^1^, M. -C. Wang^5^, Y. -C. Tseng^5^, T. Kikuchi^6^ and I. J. Tsai^1,2^

^1^Biodiversity Research Center, Academia Sinica, Taipei 115, Taiwan

^2^Biodiversity Program, Taiwan International Graduate Program, Academia Sinica and National Taiwan Normal University, Taipei, Taiwan

^3^Department of Life Science, National Taiwan Normal University, 116 Wenshan, Taipei, Taiwan

^4^Department of Microbiology, Soochow University, Taipei, Taiwan

^5^Marine Research Station (MRS), Institute of Cellular and Organismic Biology, Academia Sinica, I-Lan County, Taiwan, Department of Integrated Biosciences, Graduate School of Frontier Sciences, The University of Tokyo, Chiba, 277-8562, Japan.

### Abstract

Obtaining sufficient genomic DNA for long-read sequencing in many organisms is currently one of the major operational bottlenecks in the study of evolution and genomic diversity in nature. In this study, we employed multiple displacement amplification (MDA) and Smartseq2 to amplify nanograms of genomic DNA and mRNA, respectively from individual *Caenorhabditis elegans* nematodes. Although reduced genome coverage was observed in repetitive regions, we produced assemblies covering 98% of the reference genome from the sequenced Oxford Nanopore long reads. Annotation with the sequenced transcriptome coupled with the available assembly showed that gene predictions were more accurate, complete and contained far fewer false positives than *de novo* transcriptome assembly approaches. We sampled and sequenced the genomes and transcriptomes of 13 nematodes from Dorylaimia, Enoplia, and early-branching species in Chromadoria. These free-living nematode species had larger genome sizes, ranging from 147–792 Mb, compared to those of the parasitic lifestyle. Nine mitogenomes were fully assembled and show a complete lack of synteny to other species. Phylogenomic analyses based on the new annotations revealed strong support for Enoplia as sister to the rest of Nematoda. Our study illustrates the utility of MDA to study the genome diversity in the phylum Nematoda and beyond.

## Elucidating the role of MigPSY peptides in interaction between plants and root-knot nematode

Lin, Ching-Jung^1^, H. Z. Yimer^2^, D. D. Luu^1^, A. C. Blundell^1^, M. F. Ercoli^1^, P. Vieira^3^, V. M. Williamson^1^, P. C. Ronald^1^ and S. Siddique^2^

^1^Department of Plant Pathology, University of California Davis, One Shields Avenue, Davis, CA, 95616, USA

^2^Department of Entomology and Nematology, University of California, Davis, California, One Shields Avenue, Davis, CA, 95616, USA

^3^USDA-ARS Mycology & Nematology Genetic Diversity & Biology Laboratory, Beltsville, MD 20705, USA

### Abstract

Plant-parasitic nematodes pose a severe threat to global food production. These parasites invade plant roots and establish permanent feeding sites, which serve as their sole source of nutrients. To manipulate host responses, they secrete effectors such as phytohormones or peptides that hijack the host’s cellular machinery. Plants produce a family of peptides called *PLANT PEPTIDE CONTAINING SULFATED TYROSINE* (PSY) that promote root growth via cell expansion and proliferation. Intriguingly, the bacterial pathogen *Xanthomonas oryzae* pv. *oryzae* also produce a PSY-like peptide called RaxX (required for activation of XA21 mediated immunity X), which contributes to bacterial virulence. Our previous research has identified a group of secreted peptides called MigPSYs in root-knot nematodes (*Meloidogyne* spp.) that resemble plant PSY peptides and stimulate root growth in *Arabidopsis*. We found that *MigPSY* transcript levels are highest during the early stages of infection in rice and tomato plants. Furthermore, down-regulating expression of *MigPSY* results in reduced root galling and egg production, suggesting that the MigPSYs serve as nematode virulence factors. To gain a better understanding of the roles of MigPSYs, we plan to characterize the mechanisms underlying their function and host perception in plants. This research is expected to provide valuable insights into the mechanism of nematode infection and may lead to the development of new methods for controlling plant-parasitic nematodes.

## Struggles, frustrations, and a glimmer of hope: tales from beech leaf disease management trials

Loyd, Andrew^1^, M. Borden^1^, J. LaMondia^2^ and R. Cowles^2^

^1^Bartlett Tree Research Laboratories, 13768 Hamilton Rd. Charlotte, NC 28278

^2^Valley Laboratory, Connecticut Agricultural Experiment Station, 153 Cook Hill Rd. P.O. Box 248, Windsor, CT 06095

### Abstract

Beech leaf disease (BLD) is an emerging threat to beech trees *Fagus* spp. in the United States. Since the initial detection in 2012 in Lake County Ohio, the disease has spread to eleven additional states and one Canadian province. The disease is caused by the recently described phytophagous nematode *Litylenchus crenatae* ssp. *mccannii* (*Lcm*), which is likely an introduced exotic pathogen. Beech trees have high ornamental value in the planted landscape and are a dominant component of Eastern North American forests, providing food and shelter for wildlife, and shade for understory plants. BLD will have lasting consequences to these ecosystems in ways that are difficult to predict. Although chemical management may be difficult on a large scale (i.e., entire forests), pesticides should be an important part of a BLD integrated pest management program for landscapes and beech tree ex-situ conservation. From 2018-present, laboratory and field trials explored potential efficacy of the following active ingredients: abamectin, acephate, emamectin benzoate, fluopyram, oxamyl, paclobutrazol, potassium phosphite, and thiabendazole. Root flare injections and drenches with oxamyl in spring of 2022 suppressed the *Lcm* populations in affected *F. grandifolia* based on leaves collected in autumn compared to non-treated controls. Foliar applications of fluopyram reduced the *Lcm* populations in laboratory assays by 90% and reduced BLD symptoms of *F. sylvatica* by nearly 80% in field trials. While initial field trials used the turfgrass nematicide Indemnify (34.5% fluopyram) at a rate of 0.7 mL/L (8.5 fl oz/100 gal), laboratory assays with the landscape fungicide Broadform (21.4% fluopyram) applied at 0.6 mL/L (8 fl oz/100 gal) also proved efficacious with a 99% reduction in *Lcm* compared to non-treated controls. Field trials conducted in 2022 with Broadform applied multiple times to target the dispersal period of *Lcm* resulted in fewer overwintering *Lcm* in dormant buds in winter of 2023. Further, *in vitro* dose response assays of fluopyram against *Lcm* yielded an EC50 of 1.2 ppm, supporting the sensitivity of *Lcm* to this SDHI activity. Field trials conducted with root flare injections with abamectin, acephate, and emamectin benzoate products were not effective at suppressing *Lcm* or reducing BLD symptoms. Similarly, drenches with acephate, fluopyram, and potassium phosphite products did not prove efficacious in beech trees one year after treatment. Ongoing data is being collected from trees treated by root flare injection with thiabendazole and drenches with paclobutrazol, in addition to disease severity ratings of trees treated with oxamyl and Broadform in the experiments conducted in 2022. This presentation will highlight these efficacy trials and the needs for further BLD management strategies.

## Homeostatic plasticity: one of the mechanisms nematodes use to adapt to anthelmintic stress

Martin, Richard J.

Department of Biomedical Sciences, Iowa State University, Ames, IA 50011

### Abstract

We are interested in the mode of action and mechanisms of resistance of anthelmintic drugs used for treating nematode parasites of humans and animals. Chemotherapy with anthelmintics produces stress responses in the parasite that kills, or if it is not sufficient, initiates stress and defense responses. Nematodes are eukaryotes with 50–250 Mb genomes and nervous systems that allow worms to adapt to xenobiotics in complex ways that include: pore forming defenses pathways; epigenetic modifications; behavior changes (learning, plasticity); gene expression changes; mutations supported by selection pressures; and Phase I, Phase II and Phase III metabolism and excretion responses. Homeostatic plasticity is one of the mechanisms that excitable cells of invertebrates and vertebrates use to regulate their activity to compensate for adjustments to long-lasting stimulation. We have studied the adaptation of *Brugia malayi*, a filarial nematode that causes elephantiasis, to the selective nicotinic anthelmintic, levamisole. We find that levamisole produces: an initial spastic paralysis followed by a flaccid paralysis, and finally, a recovery of motility with loss of sensitivity to levamisole over 4h. Motility, calcium imaging, patch-clamp and molecular experiments show that the muscle ACh receptors are dynamic with mechanisms that adjust their properties and sensitivity to levamisole. I describe components of the homeostatic plasticity that allows the nematode parasite to adapt to the stress of exposure and resist effects of the anthelmintic.

## Molecular characterization of lance nematodes on creeping bentgrass putting greens in Missouri and Indiana

McCurdy, Asa^1^, J. Barizon^2^ and G. L. Miller^1^

^1^Purdue University, Dept. of Botany and Plant Pathology, 915 Mitch Daniels Blvd, West Lafayette, IN 47907

^2^University of Missouri, Soybean Cyst Nematode Lab, 1054 E. Campus Loop, Columbia, MO 65201

### Abstract

Plant-parasitic nematodes (PPN) can have detrimental effects to the health, visual quality and playability of creeping bentgrass (*Agrostis stolonifera L.*) putting greens. Lance nematodes (*Hoplolaimus* spp.) pose a significant threat due to their dual ectotrophic and migratory endotrophic feeding habits. Knowledge regarding the lance nematode species that affect putting greens in the transition zone is lacking, but may shed light on seasonal population dynamics, feeding habits and the development of appropriate management practices. Twelve samples (2 cm width, 25 cm depth) were randomly collected along a stratified W-pattern from one creeping bentgrass putting green in St. Louis in 2021 and ten in Indiana in 2022. In Indiana, lance nematodes used for molecular characterization were obtained during the months of April, June, August and October, and lance nematodes from St. Louis were obtained solely in October of 2021. Samples (100 cc volume) were processed using a semiautomatic elutriator followed by the centrifuge flotation method, then pooled to a total sample volume of 5 mL and their populations were recorded. Individual lance nematodes were separated from other PPN species by hand-picking and subsequently suspended in 200 uL of nuclease-free water. DNA was extracted from individual lance nematodes using the Extract-N-Amp tissue kit. Only amplicon bands of *H. stephanus* were detected from 15 samples on electrophoresed agarose gels following amplification with species specific primer sets of *H. stephanus*, *H. galeatus*, and *H. columbus*. The entire ITS region was amplified, sequenced and compared to Genbank accessions with phylogenetic analysis. Of 15 samples, 14 amplicons of the complete ITS rDNA region were 98–99% identical to *H. stephanus* (KP303671.1) with the other having 98–99% identity to *H. magnistylus* (KP303682.1). Scanning-electron micrographs of a lance nematode collected from an Indiana site provides morphological confirmation of *H. stephanus*. This individual had 25 longitudinal striae on the basal lip annule, and four labial annules. These results indicate *H. stephanus* is a common lance nematode species present in bentgrass putting greens in the Midwest transition zone region, conflicting with previous literature reporting *H. galeatus* as the predominant species.

## *Heterodera glycines* is associated with shifts in fungal community composition in Ohio agricultural soils

Medina López, Melanie^1^, T. Ralston^2^, H. D. López-Nicora^2^ and S. Benitez Ponce^1^

^1^The Ohio State University, Dept. of Plant Pathology, Wooster, OH 44691

^2^The Ohio State University, Dept. of Plant Pathology, Columbus, OH 43210

### Abstract

The soybean cyst nematode (SCN), *Heterodera glycines*, is the most yield-limiting pathogen of soybean, causing losses of over $1.5 billion in the U.S. Strategies to manage this nematode are limited and have focused on the use of resistant cultivars. Unfortunately, SCN is overcoming the most prevalent genetic source of resistance in commercially available soybean cultivars. Some fungi have shown promise as biocontrol agents against SCN infestation, while some pathogens of soybean have been reported to have synergistic relationships with this nematode. However, the fungi associated with field soils in Ohio are underexplored. This limits our understanding of the presence and geographical distribution of both potential biocontrol agents of SCN as well as soybean soilborne pathogens that interact with the nematode. To address this, we collected 171 soil samples from soybean fields across 26 Ohio counties in the years 2019 and 2021 to estimate SCN abundance and fungal community composition. From each sample, the abundance of SCN was determined by counting the number of nematode eggs per 100 cm^3^ of soil. This abundance ranged from 0 to 15,800 SCN eggs/100 cm^3^ of soil. From the same bulk soil samples DNA was extracted, and the ITS region of the rDNA was sequenced to characterize the fungal communities of the soil. We found that the fungal community composition of the soil was influenced by the location of sampling. This was further demonstrated using a k nearest-neighbor analysis where samples within the same region were significantly assigned as near-neighbors using their fungal community composition. SCN abundance was also identified as a significant factor influencing this composition. Principal component analysis on the fungi associated with different levels of SCN suggests that the nematode is driving selective pressure on the fungal community. Further, core community analysis showed that at different levels of SCN, different fungal taxa are classified as core members of the community. Within these taxa, at least two potential soybean pathogens were more prevalent in soils infested by SCN than in fields where the nematode was not detected. On the other hand, a potentially nematophagous fungus, *Clonostachys rosea,* was identified as a core member of all soil samples. Such fungus was enriched in soils with high levels of SCN abundance. This agrees with our current understanding of the formation of soil suppressiveness, which tends to occur under long-term high pathogen pressure. Together, these results suggest that SCN significantly shifts the fungal community composition in Ohio field soils. Additionally, the association of plant pathogens with different levels of SCN abundance could have implications on multi-pathogen interactions in soybean fields. Finally, the prevalence of nematophagous fungi in most of the fields sampled warrants further study for potential biocontrol applications.

## Investigating fitness costs associated with virulence of *Heterodera glycines* on resistant soybean

Mekidani Salu, Jacob^1^ and M. G. Mitchum^1, 2^

^1^University of Georgia, Dept. of Plant Pathology, Athens, GA 30602

^2^University of Georgia, Institute of Plant Breeding, Genetics and Genomics, Athens, GA 30602

### Abstract

Soybean cyst nematode (SCN), *Heterodera glycines*, is a major pathogen of soybean that causes significant yield losses worldwide. Host plant resistance is an effective strategy to manage SCN, but the development of resistance-breaking populations has emerged as a major challenge to this approach. Moreover, our understanding of the molecular mechanisms underlying virulence and any associated fitness costs remain unknown. In an ongoing study, we are investigating whether virulence in SCN populations is associated with a fitness cost. For this, we are evaluating the virulence and fitness of two SCN populations, derived from a population adapted to reproduce on broad-spectrum resistance, that have become less virulent over time due to their maintenance on a different host genotype. The virulence of each SCN population is being assessed based on the number of SCN cysts and eggs produced, as well as possible genetic modifications within these virulent nematodes. Our preliminary data has shown that there is a decrease in the percentage of individuals virulent on the broad-spectrum resistant genotype within each population in the absence of selection pressure. We are currently carrying out assays to compare the reproductive parameters of these SCN populations on resistant and susceptible host genotypes. Furthermore, we will be examining possible genetic modifications by carrying out metabolite and effector profiling between the parasitic life stages of these SCN populations. The results of this study could potentially aid in devising strategies to limit the selection of virulent SCN populations in the field, and may provide opportunities for the development of new strategies to manage this devastating pathogen.

## Identification and evaluation of the pathogenicity of entomopathogenic nematodes in Haiti for the production of bio-pesticide

Micodeme, Esterlin

Gent University, Dept. of Agro- & Environmental Nematology, Faculty of Science, Gent 9000, Belgium

### Abstract

Haiti is a country with an agricultural vocation since more than half of the population depends on agriculture. Roots and tubers, including cassava, sweet potato, potato, and yam, are among the most cultivated and consumed agricultural commodities in the country and are extremely necessary in the fight against food insecurity. However, the production of these plants remains very difficult due to the major problem of parasitic attacks (mainly beetles) which devastate the crops, considerably reduce the yield of agricultural plots. Farmers have always used insecticides to fight this scourge, which further complicates the situation given that these products are toxic (can have a direct impact on biodiversity and they are extremely carcinogenic to humans) and extremely expensive. Another method is biological control using entomopathogenic nematodes (EPN) as bio=pesticides that can parasitize the beetles and thus reduce the population densitie of these insects. The EPN are an ideal complement to synthetic bio-pesticides because of their environmental and human health safety characteristics (Ehlers, 1996). In this perspective, an experiment will be carried out with the aim of identifying EPN species in the Haitian environment and then isolating, characterizing and evaluating pathogenicity on the insects of choice (in that specific case the beetles). The nematodes will be isolated from the soil using an insect bait technique, morphological and molecular identification will be made. Subsequently, we will proceed to the culture of the isolated EPNs. The efficacy of the isolated EPNs will be tested on beetles (adults and juveniles) that specifically attack roots and tubers using treatments based on the number of infective juveniles (IJ) per ml of solution. We expect to evaluate five treatments which differ according to the number of IJ of the EPNs per ml of solution (0, 50, 100, 150 and 200 IJ/ml). Note that the treatment without IJ refers to the control. Once the specific nematodes have been identified, we will proceed to culture the EPN in the laboratory to have enough IJ to produce a bio-pesticide and to inoculate plants in the fields. The nematodes will be cultured in a culture medium that will provide nutrients and a favorable environment for their growth and reproduction (culture will be done in Petri dishes, where the temperature, humidity and pH conditions will be carefully controlled to ensure optimal growth). Symbiotic bacteria, which are necessary for insect control by EPN, will also be cultured in this medium. Once the nematodes are ready, they will be used to treat insect pests. The application will be made in the form of a liquid suspension, which will be sprayed on the plants or in the soil. The nematodes will enter the body of their host, where they will release symbiotic bacteria that will kill the insect.

## Nematode diversity in suspended canopy soils of the Pacific Northwest

Moats, Zachary E.^1^, J. L. Kane^1^, J. B. Kotcon^1^, K. Mafune^2^ and E. M. Morrissey^1^

^1^Division of Plant and Soil Sciences, West Virginia University, Morgantown WV 26506

^2^Dept. of Civil and Environmental Engineering, University of Washington, Seattle, WA 98026

### Abstract

Old-growth forests are some of our greatest ecological allies. These systems act as major carbon sinks by storing vast amounts of organic matter in soil and woody biomass. In the Pacific Northwest, this accumulation of organic carbon and nutrients in soil is not limited to the forest floor. In the canopy of ancient trees, soil forms on branches. These canopy soils provide a unique habitat for soil microorganisms. These organisms decompose the detritus that accumulates in the canopy, releasing nutrients back into these unique soils. These nutrients can be absorbed by trees that extend adventitious roots from their limbs into canopy soils, as well as by epiphytes growing on the large branches. Nematodes play a crucial role stimulating nutrient cycling in soils across the globe, and may be similarly important for canopy soil function, but their diversity in these ecosystems remains unknown. To address this knowledge gap, we surveyed nematode communities in canopy soils of the Pacific Northwest. Specifically, we sampled canopy soils of bigleaf maple trees (*Acer macrophyllum*) in Olympic National Park (Washington, USA). Nematode communities were extracted using Baermann funnels from 3-g samples of soils collected from 21 branches ranging from 5 to 20 meters in height across seven trees (three branches per tree), and from the forest floor adjacent to each tree. Preliminary results suggest that nematodes are at least twice as abundant in the canopy soil as in ground soil. In total, 15 families and 18 genera were observed. Of these genera, 11 were unique to the canopy while two were unique to the ground. Five genera were present in both the canopy soils and the forest floor. Of these 15 families, *Dorylaimidae* was the most abundant (31.9 % of nematodes recovered) followed by *Rhabditidae* (17.8 %) and *Tylenchidae* (11.9 %). Within the 18 genera described, fungivores, bacteriovores, and predatory nematodes were present. These data indicate that a wide range of nematode diversity, including multiple trophic levels, exists within the canopy soils, suggesting these nematodes may be key to carbon and nutrient cycling in canopy soils. Understanding how these diverse populations of nematodes interact with the rest of the microbial community to determine canopy soil function provides insights into the unique biogeochemical cycles of these ancient forests.

## Exploring distribution of nematodes in an urban ecosystem: belowground life at the Ohio State University

Mondal, Sandip^1^, T. Burgos-Hernandez^2^ and H. D. Lopez-Nicora^1^

^1^The Ohio State University, Dept. of Plant Pathology, Columbus, Ohio, 43210

^2^The Ohio State University, School of Environment and Natural Resources, Columbus, Ohio, 43210

### Abstract

Global biodiversity is significantly impacted by rapid urbanization. There is still little information available on how urbanization affects nematodes and alters soil biodiversity. We investigated the nematode community in urban soils across The Ohio State University campus (n = 99). Soil samples (500 g) were collected either from barren land or turf soil at three soil depths (0–10 cm, 10–30 cm, and 30–90 cm). Plant-parasitic nematodes (PPN) were identified up to the genus level and the rest were classified into trophic groups. Physical (bulk density, soil texture) and chemical properties (pH, EC, total-macro and micro-nutrients) of soil were analyzed. Total nematode abundance showed a decreasing pattern with increasing depth. Abundance of trophic groups and index of trophic diversity also followed a similar pattern. Among major PPN, *Helicotylenchus* was the most prominent genus followed by *Criconemella*, *Pratylenchus*, *Tylenchorhynchus*, *Paratylenchus*, *Hoplolaimus* and *Heterodera*. This pattern was consistent across all three soil depths. However, the Shannon diversity index, Margalef’s richness and Pielou’s evenness for PPN were higher in 10–30 cm than in the topsoil layer. This could be due to the dominance of *Helicotylenchus* (relative prominence value = 43.27) in surface soil over other PPN. Both the nematode channel ratio and Wasilewska index were found to be highest in the topsoil layer indicating a predominant bacterial decomposition pathway. The maximum similarity in PPN community composition was observed between 0–10 cm and 10–30 cm soil depth. Higher abundance of genera like *Xiphinema*, *Longidorus* and *Paratrichodorus* could be the reason for the distinct community composition of PPN at 30–90 cm soil depth. Soil organic carbon, bulk density, pH, and total nitrogen showed significant correlation with different nematode trophic groups. Nevertheless, no significant impact of soil texture was observed. The study generated baseline information on the nematode community in a highly disturbed soils in urban environment.

## Characterization of *Diplogasteroides* sp., A cryptic population of the *haslacheri* group; and *Parasitorhabditis terebranus* associated with monochamus alternatus from Korea

Mwamula, Abraham Okki^1,2^, S. M. Lee^3^, Y. H. Jung^3^, H. W. Lee^4^, Y. S. Kim^1,2^, Y. H. Kim^1,2^ and D. W. Lee^1,2,4^

^1^Department of Entomology, Kyungpook National University, Sangju, 37224, Republic of Korea

^2^Research Institute of Invertebrate Vector, Kyungpook National University, Sangju 37224, Republic of Korea

^3^SM Biovision Co., Jinju, 52849, Republic of Korea

^4^Department of Ecological Science, Kyungpook National University, Sangju, 37224, Republic of Korea

### Abstract

The family Diplogastridae is constituted by morphologically diverse genera. Species of *Parasitorhabditis* and *Diplogasteroides* are taxonomically confounding, with no clear genus-specific apomorphies (in *Diplogasteroides*), and phenotypic characters that overlap with other closely related genera. Integrative taxonomy considering both morphological characters and DNA-based inferences provide better supported approach in nematode identification. However, entomophilic nematodes have not been given the necessary attention, and DNA sequence data of many of these species is still unavailable. During a survey in pine forest ecosystem in Korea, a diplogastrid (*Diplogasteroides* sp.) and rhabditid (*Parasitorhabditis* sp.) nematode were recovered from the frass in beetle galleries in dead pine. The two populations were morphologically characterized and their linked DNA (18S-rRNA, 28S-rRNA, ITS-rRNA and COI) sequence data supplied. Females and males of *Diplogasteroides* sp. and *Parasitorhabditis* sp. from Korea conformed to the original species descriptions of *D. haslacheri* and *P. terebranus* from Europe and USA, respectively, with variations in a few details in morphometrics. However, despite the identical morphological congruity between the studied *Diplogasteroides* sp. and *D. haslacheri,* it cannot be designated as *D. haslacheri* due to the existence of cryptic species complex within the *haslacheri* group (*D. haslacheri*, *D. asiaticus, D. nix, D. andrassyi,* and *D. carinthiacus*); a condition that necessitates hybridization studies to allow deductions on species identity within the group. Also, based on DNA inferences, particularly the nearly full-length 18S-rRNA and D2-D3 region, very limited interspecific sequence variations of 1–7 bp has been recorded among these species. However, significant interspecific sequence differences among these cryptic species are evident in the COI gene (8.3% [51 bp] to 11.7% [72 bp]). Thus, in addition to the vital hybridization tests, the COI might be a powerful DNA barcoding marker for the precise identification of these cryptic species within the genus. This is the first molecular characterization of *P. terebranus*, and the species is herein recorded for the first time outside its type locality. The obtained and edited DNA sequences for the two species were submitted to the GenBank database under the accession numbers OQ704207 - OQ704210 (18S-rRNA), OQ291287 - OQ291290 (28S-rRNA), OQ305560 - OQ305564 (ITS-rRNA) and OQ281740 - OQ281743 (COI gene).

## Population dynamics of *Heterodera humuli* and occurrence of plant-parasitic nematodes associated with hop in the Pacific Northwest

Núñez-Rodríguez, Lester A.^1^, K. Altendorf^2^, A. Tawril^2^, D. Gent^3^ and I. A. Zasada^4^

^1^Department of Botany and Plant Pathology, Oregon State University, Corvallis, OR, USA

^2^USDA-ARS Forage Seed and Cereal Research Unit, Prosser, WA, USA

^3^USDA-ARS Forage Seed and Cereal Research Unit, Corvallis, OR, USA

^4^USDA-ARS Horticultural Crops Disease and Pest Management Research Unit, Corvallis, OR, USA

### Abstract

The United States is the world’s largest producer of hop with 40% of the worldwide acreage, with production valued at $618 million in 2022. The Pacific Northwest (PNW; Washington, Oregon, and Idaho) accounted for ~98% of the US hop production. Hop is affected by different biotic factors, among them, plant-parasitic nematodes (PPNs). Of the PPN, *Heterodera humuli* is the most common in hop. Despite this, little is known about the: 1) population dynamic of *H. humuli* under field conditions, and 2) occurrence and distribution of PPNs in hop. In April 2022, a two-year population dynamic study was initiated. One field in Washington and Oregon were selected for sampling weekly from April to September and monthly from October to March. There was a peak in *H. humuli* second-stage juveniles (J2) densities in soil by the end of May, and then a decline with the lowest densities observed in August. During fall 2021 and 2022, a survey of PPNs in hop was conducted in the PNW. A total of 185 soil samples were collected from 24, 28, and 41 hop yards in Idaho, Oregon, and Washington, respectively. Ten different PPN were identified. *Heterodera humuli* J2 were present in 54% of samples with an average density of 45 J2/250 g of soil. *Heterodera humuli* cysts were found in 75% of samples with a maximum density of 804 cysts/100 g of dried soil. Other PPN commonly found in hop yards were *Bitylenchus*, *Tylenchorynchus* and *Helicotylenchus* with >30% occurrence. When separated by state, Washington had the highest diversity with 10 PPNs; *H. humuli* was the most frequent PPN encountered with 67% occurrence, followed by *Bitylenchus*, *Tylenchorynchus* and *Helicotylenchus* with >50% occurrence. Oregon had the lowest diversity with seven different PPNs, with *H. humuli* being the most frequent. Three Trichodoridae and one *Pratylenchus* population from Idaho were molecularly identified using the *28S* gene and the partial *cox1* gene, respectively. The Trichodoridae had 100% identity with *Paratrichodorus allius*, while the *Pratylenchus* population had 100% identity with *Pratylenchus neglectus*. Our results show that *H. humuli* has one generation per year and that this species is widely distributed in PNW hopyards. Additionally, some of the most important PPN in the PNW, such as *Pratylenchus*, can be found on hop in the region. Future research should focus on determining the pathogenicity of PPN to hop and the development of management strategies.

## Distribution, abundance, and virulence profile of soybean cyst nematode (SCN) in Ohio

Newman, Emma^1^, Z. Ralston^1^, A. Dorrence^2^ and H. Lopez-Nicora^1^

^1^Department of Plant Pathology, The Ohio State University, Columbus, OH, USA

^2^Department of Plant Pathology, The Ohio State University, Wooster, OH, USA

### Abstract

The soybean cyst nematode (SCN) is the most economically important pathogen of soybean in North America. SCN continues to spread throughout Ohio. Samples from 2018 to April 2023 were submitted for testing from 62 of 88 Ohio counties. A total of 1,163 samples were tested during that period, with over 63% positive for SCN. Most samples had less than 2,000 eggs per/100 cm^3^ soil and less than 10% had more than 5000 eggs/per 100 cm^3^ soil. Soil samples that had SCN levels greater than 500 eggs/100 cm^3^ soil (n = 64) underwent a modified HG type test, here in after referred to as a SCN type test. Indicator lines used for the SCN type test were PI 548402 (Peking), PI 88788, and PI 437654, additionally cv. Williams 82 and Lee 74 were used as susceptible checks to generate the female index. More than 85% of the SCN populations in Ohio can reproduce on PI 88788 (SCN Type 2) at levels between 30–60% of a susceptible soybean. There are few SCN populations that can reproduce on Peking (SCN Type 1) at very lows levels (10–30% of susceptible) PI 437654 remains highly resistant to our SCN populations, but it is not easy to find soybean cultivars with the source of resistance. Of the 64 samples that were type tested, 17% were SCN type 0, therefore any resistance would be effective. Additionally, soil from each field was analyzed for edaphic factors showing a correlation to SCN population density for several variables. Active management of SCN begins by knowing your numbers, but most importantly knowing your SCN type will improve soybean cultivar selection with effective SCN resistance.

## Strategies to develop and apply a test for detection of ringspot viruses in dagger nematodes in small fruits and grapevines

Olaya, Cristian^1,3^, L. Reinhold^2^, D. Mollov^3^ and I. A. Zasada^3^

^1^ORISE-Oak Ridge Associated Universities, USA

^2^USDA-ARS, National Clonal Germplasm Repository, Corvallis, OR 97330

^3^USDA-ARS Horticultural Crops Disease and Pest Management Research Unit, Corvallis, OR 97330

### Abstract

Small fruits and grapevine are important commodities for the Pacific Northwest (PNW) of the United States. Tomato ringspot virus (ToRSV) and tobacco ringspot virus (TRSV) have been reported in the PNW as well as their vector, the dagger nematode (*Xiphinema americanum sensu lato*). The presence of both virus and vector represents a risk factor for susceptible crops. Currently available diagnostic methods are likely missing low titer infections or unknown ToRSV and TRSV variants. Also, lack of sensitivity makes it difficult to detect the viruses in the nematode. In this research we focused on ToRSV to develop a sensitive test to detect the virus in the dagger nematode. To achieve this goal, we first compiled a diverse collection of at least 33 ToRSV isolates for primer design; second, we surveyed small fruit crops in the PNW to determine the presence of the virus and the vector; third, we created a growth chamber protocol to produce dagger nematodes that harbor ToRSV. Available RT-qPCR assays detected 32 of 33 ToRSV isolates enabling the development of new primers for more comprehensive virus detection. Most of the samples collected in 2021 in the PNW tested negative for ToRSV. Only currants (*Ribes* spp.) tested positive for ToRSV. Dagger nematodes were found in 29% and 38% of samples collected in OR and WA from grapes and small fruit crops. When detected, population densities of dagger nematodes ranged from six to 451 per 250 g of soil. For the development of a protocol to produce nematodes that harbor ToRSV, cucumber plants were mechanically inoculated with ToRSV and exposed to at least 20 dagger nematodes. RNA from single nematodes will be isolated and tested for ToRSV. Preliminary results show that dagger nematodes are widespread on grapes and small fruits in the PNW and that there is a need for sensitive and comprehensive detection methods for ToRSV.

## Potato cyst nematodes in Kenya

Orage, Calvince^1^, D. L. Coyne^1^, L. M. Dandurand^2^, S. Haukeland^3^, J. Jones^4^ and C. Opperman^5^

^1^International Institute of Tropical Agriculture (IITA), PO Box 30772-00100 Nairobi, Kenya

^2^University of Idaho, 875 Perimeter Drive MS 2332, Moscow, ID 83844-2332, USA

^3^International Centre of Insect Physiology and Ecology (ICIPE), P.O. Box 30772-00100 Nairobi, Kenya

^4^Norwegian Institute for Bioeconomy Research (NIBIO), PO Box 115, NO-1431 Ås, Norway, Uganda, Cell & Molecular Sciences Department & Social Economic and Geographical Sciences Department, The James Hutton Institute, Dundee DD2 5DA, UK

^5^Department of Entomology and Plant Pathology, North Carolina State University, Box 7903, Raleigh, NC 27607

### Abstract

In Kenya the golden nematode (GN), *Globodera rostochiensis*, a globally regulated pest, causes an 80% yield reduction in potato. Infestations of this nematode has caused farmers to shift to different crops. In Kenya, potato is the second most consumed crop after maize. However, the dominant cultivar, Shangi, is highly susceptible to GN. Difficult to manage, growers are seeking better control measures for this plant parasitic nematode. Researchers are conducting trials on innovations to suppress GN levels and increase yields. Resistance to GN in cultivars with similar traits as Shangi is being assessed under Kenyan field conditions. Eight test lines prevented multiplication of PCN in field trials and increased yields. In addition to screening for resistance, a study was conducted to evaluate consumer acceptance based on sensory evaluation of either the boiled-whole or boiled-mashed Shangi-like potato clones. The study consisted of 600 farmers, stakeholders, university personnel, or college students from different locations. They appreciated the new lines and by choice preferred the new clones over Shangi. Farmers also indicated willingness to pay more for PCN-resistant varieties. The best two performing lines in terms of high yielding, PCN resistance, and consumer acceptance are now being assessed in multilocation trials for future release in Kenya. These new lines offer a potential option for the management of PCN. Additionally, our innovative “Wrap and Plant’ technology,” where potato is wrapped with biodegradable banana paper treated with ultra-low doses of fluopyram (a conc. of 0.0125 ml of Velum + 6.8 ml of H_2_O per A4 size paper) is being tested in infested fields in Kenya. Trials have been conducted over the period of five seasons in all potato growing areas in 4 × 3m plots with 50 tubers planted per plot. For each plot, soil was sampled, and nematodes were extracted from 200g by using a Fenwick can to obtain initial and final populations. We have found a significant (*P* < 0.05) yield increase and PCN suppression when a susceptible potato is treated with our “Wrap and Plant’ technology compared to the non-wrapped treatment. In addition to controlling GN, the treated paper promoted populations of beneficial nematode and provided zero emission due to nanogram doses applied which qualifies the ‘Wrap and Plant’ technology as a climate smart innovation.

## A new biological product shows promising control of the northern root-knot nematode, *Meloidogyne hapla*, in greenhouse tomatoes

Palmisano, Abigail, E. Darling, H. Chung, and M. Quintanilla-Tornel

Department of Entomology, Michigan State University, 288 Farm Ln, East Lansing, MI, 48843

### Abstract

The northern root-knot nematode, *Meloidogyne hapla,* is a significant pest that causes yield loss and affects development of it’s host plants, such as tomatoes. While conventional methods of chemical management have been utilized for nematode control in the past, there has been a growing demand by growers for more environmentally considerate and organic means of control. For this study, we established a greenhouse trial of susceptible tomato (cv. Rutgers) plants and studied the potential of a new, biologically based product (referred to as ‘MN21.2’) on controlling *M. hapla* populations. The outcomes of this study may provide organic tomato growers with valuable strategies to manage the plant parasitic nematode, *M. hapla* if product efficacy is supported under field conditions.

## Manure-based amendments influence on soil biological parameters and its correlation with *Pratylenchus penetrans* infection in potato plants

Parrado, Luisa, E. Darling and M. Quintanilla

Michigan State University, Department of Entomology, East Lansing, MI, 48824

### Abstract

Michigan ranks 8th in the nation with more than 17,806 ha of potato and a $1.24 billion economic value. In potato production, the root-lesion nematode *Pratylenchus penetrans* can interact with the wilt-inducing fungi *Verticillium dahliae* causing a disease known as potato early die. This disease causes severe damage to the plant root and vascular system, reducing yield by 30 – 50%. In Michigan, pre-potato planting soil fumigation with metam sodium is the most effective way to reduce early die complex inoculum. Nevertheless, early die management remains one of the top industry priorities in Michigan potato production, highlighting the need for new sustainable management alternatives. Applications of raw poultry manure and a compost blend made from poultry and cattle manure have been significantly effective at reducing *P. penetrans* infection in potatoes. It has been hypothesized that the pesticidal properties of organic soil amendments come from the diversity of their microbial communities. However, the contribution of microbes from the manure-based amendments to the native soil microbial communities where potatoes are generally grown has not been elucidated. Therefore, the main goal of this study was to determine if the influence of these contrasting manure-based amendments on soil microbial communities correlated with reductions of *P. penetrans* infection in potato plants. To accomplish this, a greenhouse experiment was set up using field soil from a commercial potato field with a known history of conventional management for early die. Certified disease-free potato ‘Russet Norkotah’ were used, and the treatments were poultry manure autoclaved vs. non-autoclaved, the compost blend autoclaved vs. non-autoclaved, Velum Prime, and sterile water as a control. Each treatment had five replications, each inoculated with 3 *P. penetrans*/g of soil, and the experiment was repeated twice. Soil samples for microbial analysis and nematode quantification were taken before treatment application, mid-season, and harvest. In addition, plant height measurements were taken every week and root samples were taken at mid-season and at harvest. Our results show that *P. penetrans* reproduction in potato roots was significantly lower in soil treated with poultry manure compared to the compost blend. Poultry manure significantly increased the overall soil microbiome functionality. Microbes with fungicidal, nematicidal, and insecticidal activity significantly increased overtime as well as those associated with enhancing plant defense and growth. Results provide evidence of the role of enhancement of soil microbial functionality on *P. penetrans* suppression in potato plants by applications of manure-based amendments.

## Harnessing the power of sorghum-sudangrass hybrids to transform soil health in annual row crop systems: a narrative on nematodes and beyond

Paudel, Roshan

Department of Plant and Environmental Protection Sciences, University of Hawaii at Manoa, Honolulu, HI 96822

### Abstract

Managing soil health in tropical Oxisols for an annual row crop rotation system is challenging. The combination of slow rate of soil carbon accumulation, high rate of organic materials degradation, year-round reproduction of soil-borne pathogens and nematodes, and year-round weed pressure pose significant obstacles to adopt conservation tillage. Sorghum and sorghum-sudangrass hybrids (SSgH) exhibit versatile properties that can resolve multiple soil health issues when managed properly. SSgH are fast-growing, carbon rich, drought-tolerant plants with deep roots that enhance soil tilth and contain the allelopathic compound dhurrin, which converts to hydrogen cyanide upon tissue maceration and acts as a biofumigant against nematodes and fungal pathogens. However, there are different varieties of SSgH awaiting for evaluation. The overarching goals of this Ph.D. project were to 1) identify the best varieties of SSgH, 2) determine the optimal termination method for SSgH cover crops to enhance soil health, and 3) unravel the mystery of soil health indicators that are meaningful to farmers or agricultural industries. Four greenhouse trials were conducted to compare the amendments of SSgH varieties to sunn hemp and an unamended control against *Meloidogyne incognita* infection on mustard green ‘Hirayama’ and *Rotylenchulus reniformis* on ‘California Blackeye’ cowpea. Energy sorghum ‘NX2/NX-D-61’, and SSgH ‘LA/Latte’ and ‘542×43’ were the most suppressive to *M*. *incognita* and *R. Reniformis*. Field trials were conducted to compare seven SSgH varieties to bare ground followed by eggplant cropping. Energy sorghum ‘NX2’ had the highest plant biomass, soil organic carbon, soil moisture, soil microbial respiration, and microbial biomass in all trials. Though sorghum improved many soil health properties, it did not suppress plant-parasitic nematodes or improve water infiltration in a no-till system. When SSgH varieties were terminated by strip- and low-till in a successional field trial, water infiltration was improved along with volumetric aggregate stability. In addition, ‘NX2’ sorghum increased total nematode metabolic footprints including enrichment, and structure footprints compared to the bare ground control. Canonical correspondence analysis of nematode and other soil health indicators from strip-till trials consistently revealed a positive relationship between eggplant yield, soil moisture, and biomass of arbuscular mycorrhizal fungi. SSgH biomass and biomass of saprophytic fungi were positively related to soil carbon. In summary, ‘NX2’ sorghum transformed the soil food web from a highly degraded and disturbed condition to a highly enriched and less disturbed state within 2 years of a SSgH-eggplant rotation. This study highlights the potential benefits of SSgH as a cover crop for improving soil health in degraded tropical soils. More work is needed to maximize eggplant yield in a sorghum-based crop rotation system. In addition, this study shows an opportunity for SSgH seed industries to breed and select SSgH varieties that offer not only high biomass yield but also significant soil health benefits.

## How long does it take for a low-till sorghum/sorghum-sudangrass cover cropping to improve soil health in a degraded tropical Oxisol?

Paudel, Roshan and K. -H. Wang

Department of Plant and Environmental Protection Sciences, University of Hawaii at Manoa, Honolulu, HI 96822

### Abstract

Sorghum and Sorghum-sudangrass hybrids (SSgH) are versatile cover crops that add soil organic matter, improve soil tilth, and increase water retention, while suppressing soil-borne pathogens and weeds. Seven SSgH varieties were examined for their efficiency in 1) suppressing *Rotylenchulus reniformis* through allelopathic effects, 2) soil water conservation, and 3) overall soil health improvement in a degraded tropical Oxisol. Two 8×3 (varieties × plant age) factorial designed greenhouse trials were conducted to compare the allelopathic effects of 1-, 2-, and 3-month old tissues from 7 SSgH varieties and sunn hemp (*Crotalaria juncea*) against infection of cowpea by *R*. *reniformis* in sterile sand:soil mix. An unamended control was included. The sand:soil mix was amended with macerated tissues of the respective plant tissues at 1% (dw/dw). A ‘California Blackeye’ cowpea (*Vigna unguiculata*) seedling was planted and inoculated with 200 juveniles of *R. reniformis* per pot. Number of females per gram of root were examined at 2 months after inoculation through acid fuchsin root staining. Energy sorghum ‘NX-D-61’ (hereby abbreviated as ‘NX2’) and SSgH ‘Latte’ and ‘542’ had the lowest number of *R. reniformis* females regardless of the age of the tissues. All other SSgH had higher female development for 2- and 3-month-old tissues although the SSgH were all lower than the unamended control. Two years of a successional field trial was conducted at Poamoho Experiment Station where 7 SSgH varieties were grown for 3 months, terminated using a flail mower followed by cultivating eggplant (*Solanum melongena*) for 5 months. SSgH biomass was soil incorporated following a strip- and low-till practice (20 cm wide, 10 cm deep) using a handheld tiller. While SSgH ‘542’and ‘NX2’ produced the greatest biomass in both years, ‘NX2’ resulted in greater nematode community structure index and total nematode metabolic footprints of free-living nematodes at the end of one cycle of SSgH-eggplant. In year 2, all SSgH increased total nematode metabolic footprints including enrichment, and structure footprints compared to the bare ground control. The improvement in soil food web structure by ‘NX2’ was accompanied by higher soil carbon and water infiltration (year 1), and volumetric aggregate stability (year 2). In both trials, total microbial biomass estimated by phospholipid fatty acid (PLFA) analysis was increased by ‘NX2’ 3 months after growing SSgH, though this effect dissipated towards the end of the eggplant crop. At 3 months after growing SSgH in both years, ‘Piper’ and ‘NX2’ enhanced the abundance of gram-negative and gram-positive bacteria, respectively. Greenhouse and field experiments suggested that ‘NX2’ was the most effective SSgH cover crop variety for suppression of *R. reniformis* and soil health improvement in Hawaii. In summary, ‘NX2’ sorghum transformed the soil food web from a highly degraded and disturbed condition to a highly enriched and less disturbed state within 2 years of a SSgH-eggplant rotation.

## Early detection of soybean cyst nematode (SCN)-infected soybean plants using near-infared (NIR) reflectance spectroscopy and machine learning

Peart, Alison^1^, T. Ralston^1^, C. Fearer^1^, A. O. Conrad^2^, P. Bonello^1^ and H. D. Lopez-Nicora^1^

^1^Department of Plant Pathology, Ohio State University, Columbus, OH 43210

^2^USDA Forest Service, Northern Research Station, West Lafayette, IN 47907

### Abstract

The soybean cyst nematode (SCN) is one of the most damaging soybean pathogens in North America. It is also one of the most insidious because it often causes significant yield reduction with no visible symptoms, which makes early detection critical for effective disease management. SCN detection is typically done through soil sampling, which can be incredibly labor-intensive and time-consuming depending on the size of a field, in addition to only being able to detect presence, but not exact location, within a field. A potential alternative to soil sampling is detection via near-infrared (NIR) reflectance spectroscopy, coupled with machine learning. This approach is based on the ability of this technology to detect host differences due to responses to an active infection. During the 2021 and 2022 growing seasons, soil samples were collected at planting from a field with an uneven distribution of SCN divided into a grid of 100, 28 × 33 ft quadrats to determine initial SCN populations (Pi). At the V9-V10 plant growth stage, NIR spectra were collected from five plants in the center of each quadrat. Quadrats included in the analysis had either high SCN populations (Pi > 1,000 eggs/100 cm^3^ soil) or were SCN-free (Pi = 0 eggs/100 cm^3^ soil). The relationship between soybean yield and the initial SCN population at planting (Pi) was also evaluated. Additionally, a similar greenhouse study was conducted using both inoculated and uninoculated plants. In both field and greenhouse studies, the foliage of infested and non-infested plants was visually indistinguishable. We are now in the process of analyzing the NIR spectral data by partial least squares discrimination analysis and support vector machine, a form of machine learning. If the model is successful at discriminating the leaves of highly SCN infested plants from SCN-free plants, NIR spectroscopy may be a useful tool for early detection of SCN and a valuable component of more proactive management strategies for this deceptive disease.

## Strategic rotations of resistance genes to combat soybean cyst nematode virulence

Pennewitt, Monica G. and G. L. Tylka

Department of Plant Pathology, Entomology and Microbiology, Iowa State University, Ames, IA 50011

### Abstract

Resistant varieties are critical for reducing soybean yield loss from the soybean cyst nematode (SCN). Although there are multiple plant introduction (PI) lines available to use for breeding that may provide host resistance against SCN, PI 88788 (*rhg1-b)* is the source of resistance used in 95 percent of soybean varieties for the state of Iowa over the past three decades. Prolonged, widespread use of PI 88788 resistance has resulted in increased virulence of SCN populations on this extremely common source of resistance. Rotating soybean genotypes with different resistance gene combinations has shown promise in lowering SCN population densities and preventing increases in virulence in greenhouse experiments. The objective of this experiment was to evaluate how rotating soybean lines containing various resistance gene combinations affect SCN population densities and the virulence of SCN populations in the field. Microplot experiments were established in Illinois, Iowa, and Missouri in 2019 and conducted for four growing seasons. Treatments included continuous planting of a susceptible genotype, soybean genotypes derived from a single source of resistance (PI 88788 with *rhg1-b*, Peking with *rhg1-a+rhg2+Rhg4*, PI 90763 with *rhg1-a+rhg2+Rhg4+*additional gene(s)), and novel genotypes containing pyramided resistance genes selected from *G. soja* and Chr. 10 from PI 567516C (pyramid 1 with *rhg1-b+G.soja*, and pyramid 2 with *rhg1-b+G. soja*+Chr.10). Also, additional two-year rotations of genotypes containing *rhg1-b* and the two gene pyramids with PI 90763 or *rhg1-a+Rhg4* were studied. The data presented herein are from the two Iowa experiments. In general, SCN population densities increased in all microplots over four years and year-to-year differences occurred among plots with rotated soybean genotypes, but results varied somewhat between locations. For most plots, rotated treatments had lower population densities compared to the treatments planted with the same genotypes continuously. Gene pyramid 2 (*rhg1-b+G. soja+*Chr.10) rotated with PI 90763 (*rhg1-a, Rhg4, rhg2*) resulted in the greatest suppression of SCN egg population densities at the end of the four-year experiment at both locations. Although this rotated treatment decreased the egg population density, the virulence of the SCN population increased, as reflected by the female index (FI) of the SCN population. In the Ames experiment, the SCN population used to infest the soil had an initial FI of 7 on PI 90763, however the continuous PI 90763 treatment and the rotation of pyramid 2 with PI 90763 caused the FI to increase to 27 percent and 12.5 percent, respectively, over three field seasons. The FI on PI 88788 remained well above 10 percent across all plots, even in populations not exposed to the *rhg1-b* gene. Additional shifts in virulence were observed but were less substantial in comparison. The results reinforce the concept that increased population densities and virulence are directly correlated to the selection pressure placed upon an SCN population and the continual use of similar resistance genes facilitate this shift over time.

## Evaluating soil health benefits of four tropical cover crops in the tropic

Pitiki, Melanie, B. Wiseman and K. -H. Wang

Department of Plant and Environmental Protection Sciences, University of Hawai‘i at Mānoa, Honolulu, HI 96822

### Abstract

Sweetpotato is a high yielding crop prone to plant-parasitic nematode (PPN) infection and responsive to soil health management. Objectives of this study were to identify tropical cover crops most efficient in carbon (C) building, water conservation and PPN suppression. A field trial was conducted at Poamoho Research Station comparing nematode suppression and contributions to soil health by: sunn hemp (SH; *Crotalaria juncea*), sorghum (SG; *Sorghum bicolor*), marigold (*Tagetes erecta*), and velvet bean (VB; *Mucuna pruriens*). Bare ground (BG) was included as control. The experiment was arranged in a randomized complete block design with four replications. All cover crops were terminated three months after planting (MAP) by flail mowing and biomass incorporated into the soil with a two-tine tiller (10-cm deep, 20-cm wide). Sweet potato was planted thereafter. Soil samples were collected at time of cover crop planting, 86 and 119 days after planting. Sorghum, VB, and SH produced abundant biomass (12, 8, 7 Mg/ha, respectively), but only VB contributed to significantly higher total soil C compared to BG (*P* ≤ 0.05). All cover crop treatments did not affect water infiltration rates and soil aggregate stability compared to BG at 3 MAP. Although none of the cover crops affected overall soil microbial respiration rates based on Solvita Burst Test, phospholipid fatty acid (PLFA) analysis showed VB increased microbial diversity, gram-negative bacteria biomass, total fungi biomass, arbuscular mycorrhizal fungi (AMF), and higher fungi: bacteria ratio (F/B) (*P* ≤ 0.05). Ammonia concentration was higher in SH and VB compared to other treatments. Canonical Correspondent Analysis (CCA) revealed that the first two canonical axes of the environmental and species variables explaining 77.71% of the variations. The CCA showed that: 1) cover crop biomass was positively related to nematode Enrichment index (EI), abundance of bacterivorous and fungivorous nematodes, and volumetric soil aggregate stability, besides microbial diversity and actinomycete biomass; 2) structure index (SI) of nematode communities were positively related to abundance of omnivorous nematodes, volumetric soil moisture (VSM), water infiltration, and total living microbial biomass (TLMB); unfortunately 3) cover crop biomass was negatively related to total C and NH_3_, microbial respiration, nematode richness, and abundance of PPN. However, this CCA also revealed that if total soil C were to be increased, it would be positively related to biomass of saprophytic fungi, AMF and F/B and would also lead to lower abundance of PPN. A scatter plot of all samples in CCA showed a distinct soil conditions of VB from all other treatments, and was most distant from BG. These results suggested that VB is the most promising tropical cover crop tested here based on the significant microbial profile changes. At this point, all PPN were below economic thresholds and not different among treatments. The experiment is ongoing to examine the longer-term impact of VB following a sweet potato cultivation.

## Aftermath of an environmental disaster: can nematodes provide insight?

Powers, Thomas^1^, P. Mullin^1^, R. Higgins^1^, T. Harris^1^, N. Gonzalez^1^, M. Rawlings^2^ and K. Powers^1^

^1^University of Nebraska-Lincoln, Dept. of Plant Pathology, Lincoln, NE 68503

^2^University of Nebraska-Lincoln, School of Natural Resources, Lincoln, NE 68503

### Abstract

In summer of 2017, the observation of mass honeybee die-offs at the University of Nebraska’s Eastern Nebraska Research and Extension Center (ENREC) led to an investigation that ultimately involved over two dozen federal and state agencies, non-profit organizations, and multiple universities and university departments. The disaster was an unanticipated result of producing ethanol from unsold pesticide treated seed. Wind-driven dispersal of exposed portions of an 84,000-ton pile of contaminated by-product (wet cake) and leakage from four lagoons for liquid waste totaling 175 million gallons contributed to excessively high levels of pesticides and break-down products in local streams and soil. Last year, in the summer of 2022, the UNL Water Center took a sample of at least 100 ml of water from six stream sites that were also sampled for nematode community analysis. The water samples were analyzed for the major pesticides commonly used in seed coatings; eight neonicotinoid and organophosphate insecticides, five strobularin fungicides and seven neonicotinoid degradation products. Abamectin was separately detected from three samples in and near the wastewater lagoons. Some of the wet cake was also distributed to local fields as a soil amendment. As part of a One-Health initiative to evaluate the impact of the contamination, we initiated a nematode survey of the streams and stream banks. To date, analysis of seven paired bank and sediment samples from the eastern watershed of ENREC has resulted in 83 morphologically identified nematode genera. Twelve genera were exclusively collected from stream sediment and 29 genera exclusively from soil banks. *Monhystera* and *Tobrilus* were the most frequently collected of the exclusively stream taxa. Previous studies have indicated the adaptability and persistence of tobrilids in extreme environments. However, the sediment sites with the highest levels of pesticide contaminants had the lowest levels of tobrilids.

## Investigating the relationship between soil nematode response and herbicide sensitivity of hemp (*Cannabis sativa*)

Ralston, Timothy^1^, S. Mondal^1^, A. Essman^2^, M. Kelly^1^ and H. D. Lopez-Nicora^1^

^1^The Ohio State University, Dept. of Plant Pathology, Columbus, Ohio, 43210

^2^The Ohio State University, Dept. of Horticulture and Crop Science, Columbus, Ohio, 43210

### Abstract

Ohio Senate Bill 57 was enacted on July 30, 2019 legalizing hemp (*Cannabis sativa*) production in the state. Consequently, field trials have become necessary to provide extension support to growers. A field trial was conducted in 2022 at Western Agricultural Research Station, South Charleston, OH to determine herbicide sensitivity of hemp and its relationship with soil nematode community. Vegetative clones of hemp (cv. Tangerine) were propagated under greenhouse conditions for 6 weeks. Dicamba and a mixture of glyphosate + dicamba were applied at the manufacture’s full label rates and sublethal rates (1/10x, 1/100x, 1/1000x, 1/10000x). Two days post herbicide application, hemp individuals were transplanted into the field site in rows covered with weed barrier fabric in a randomized complete block design. Before transplanting, soil samples were collected to enumerate the initial nematode population. Three months after transplanting and prior to termination, rhizospheric soil samples were collected to quantify any change in nematode population dynamics. A total of 55 nematode genera were identified from the hemp ecosystem. The initial soil nematode community was dominated by *Tylenchus* (relative prominence value, RPV=21.3), *Panagrolaimus* (13.5), *Filenchus* (13.1), *Aphelenchus* (12.9), *Rhabditis* (10.9), and *Aphelenchoides* (6.6). However, a significant increase of fungivore population (*Aphelenchus*, RPV= 33.5) was observed at the end of the season. Among economically important plant-parasitic nematodes, *Helicotylenchus* (RPV=14.8) was found to increase approximately 7-fold and thus could be one of concern to growers. Significant increases in nematode abundance, richness (Margalef’s index), and evenness (Heip’s index) were observed between planting and harvest. However, no significant changes were noticed between herbicide treatments. The Shannon diversity index did not vary either across treatment or time. Moreover, functional diversity measures, like maturity index, structure index, and enrichment index also showed no significant variation across treatments and times. This study generated baseline information on nematode community assemblages under hemp cultivation in Ohio.

## Detection and distribution of *Meloidogyne* species infecting subtropical crops in Florida

Riva, Gabrieli, H. X. Bui, M. Gu and J. A. Desaeger

Department of Entomology and Nematology, University of Florida Gulf Coast Research and Education Center, Wimauma, Florida, 33598

### Abstract

Although root-knot nematodes (RKNs; *Meloidogyne* spp.) are widespread in Florida, little is known about the distribution of different RKN species across the state. This is especially important in light of recent reports of *M. enterolobii* in the southeastern United States. As part of the FIND*Me* project (Focused Investigations on the Distribution and management of *Meloidogyne enterolobii*), we conducted a survey and collected infected roots and soils from 56 crops belonging to 22 different families cultivated in commercial vegetable and fruit farms, research institutions, horticultural gardens, and natural landscapes in 12 Florida counties, including Alachua, Charlotte, Collier, DeSoto, Hardee, Hendry, Highlands, Hillsborough, Manatee, Miami-Dade, Palm Beach, and Sarasota. We employed molecular methods to identify *Meloidogyne* species by using primer sets (TRNAH/MRH106) and (MORF/MTHIS). Also, *Meloidogyne* species-specific primers were used to confirm species identification. We sequenced mtDNA for identification when the two mentioned methods were inconsistent and submitted the results to the genbank (National Center for Biotechnology Information). *Meloidogyne* species were detected in 247 (81.25%) out of 304 soil and root samples collected in Central and South Florida between September 2019 and January 2023. Five *Meloidogyne* species (*M. arenaria*, *M. enterolobii*, *M. hapla*, *M. javanica* and *M. incognita*) were identified. *M. incognita* and *M. enterolobii* were the most prevalent species representing 76 (25%) and 76 (25%) samples, respectively. *M. javanica* was identified in 50 (16.44%) samples, followed by *M. arenaria* in 25 (8.22%) samples and *M. hapla* in 16 (5.27%) samples. Mixed populations of *M. enterolobii* and *M. incognita* were identified in 4 (1.31%) samples. These findings provide new insights into the distribution and prevalence of *Meloidogyne* species in Central and South Florida. It is clear from our survey that *M. enterolobii* is much more common in Florida soils than previously thought. Our survey is the first of its kind to study the distribution of *Meloidogyne* spp. in Central and South Florida which will help to develop more effective integrated nematode management strategies. We plan to continue the survey to include other counties and crops in order to more accurately map the distribution of RKN species in Florida.

## Dorsal gland RNA sequencing reveals novel effector candidates in *Meloidogyne incognita* adult females

Rocha, Raquel^1,3^, R. S. Hussey^1^, L. E. Pepi^2^, P. Azadi^2^ and M. G. Mitchum^1^

^1^University of Georgia, Dept. of Plant Pathology and Institute of Plant Breeding, Genetics, and Genomics, Athens, GA 30602

^2^Complex Carbohydrate Research Center, University of Georgia, Athens, GA 30602

^3^The Connecticut Agricultural Experiment Station, New Haven, CT 06511

### Abstract

Root-knot nematodes (RKN) represent one of the most economically important groups of plant-parasitic nematodes. When forming the gall, or “knot”, the RKN uses a protrusible stylet to secrete a cocktail of effectors that re-program host cells for their benefit. These effectors are produced within specialized secretory esophageal gland cells, one dorsal (DG) and two subventral (SvG), whose activity differs throughout the nematode life cycle. Significant research has been done to profile RKN effectors and their roles in parasitism. However, most studies have focused on early events of the RKN life cycle and the effectors that are targeting processes required for penetration and initiation of a feeding site. Here, we developed a new approach to enrich for the highly active DG cells of RKN adult females to identify the effectors, and ultimately, the processes they target in the host during late parasitism. A combination of manual cutting, sonication, vortexing, and filtering of female heads and their contents was used to obtain DG-enriched samples for transcriptomic and proteomic analysis. RNA-seq pairwise comparisons of DG-enriched samples with pre-parasitic second-stage juveniles and female heads led to the identification of 83 effector candidates with a predicted signal peptide but lacking transmembrane domains or homology to proteins in *Caenorhabditis elegans*. No associated functional annotation was found for 80 candidates. Of these, 19 were previously shown to be expressed in RKN esophageal gland cells. Thus, the remaining 64 represent newly discovered DG effector gene candidates expressed in adult females. Twenty-eight of the 83 candidates were also identified via an exploratory glycoproteomic analysis. *In situ* hybridization confirmed the DG-specific expression of 14 of these effector candidates in adult females. The lack of information available about such proteins reveals how much we still need to uncover about the key cellular and biochemical processes mediating nematode parasitism. Ongoing studies will further elucidate the mechanism of action of such candidates and the components they interact with in the host.

## Genetic resistance to *Meloidogyne javanica* in cucumber is conferred by mutations upstream of a host transcription factor that positively regulates defenses

Rutter, William^1^, A. Hajihassani^2^, Y. Wang^3,4^ and Y. Weng^3,5^

^1^United States Vegetable Laboratory, Charleston, SC 29449

^2^Department of Entomology and Nematology, Fort Lauderdale Research and Education Center, University of Florida, Davie, FL 33314

^3^Horticulture Department, University of Wisconsin, Madison, WI 53706, USA

^4^State Key Laboratory of Crop Genetics and Germplasm Enhancement, College of Horticulture, Nanjing Agricultural University, Nanjing, Jiangsu 210095, China

^5^Vegetable Crops Research Unit, USDA-ARS, 1575 Linden Drive, Madison, WI 53706, USA

### Abstract

The Javanese root-knot nematode (JRKN, *Meloidogyne javanica*) is a serious pest of cucumber (*Cucumis sativus*) and other vegetable crops grown in tropical and subtropical regions. The recessive *mj* resistant gene provides wild cucumber (*C. sativus* var. *hardwickii*) with species-specific resistance to JRKN and is currently the only reported source of resistance to any RKN species within *C. sativus*. In this study, we conducted genetic mapping and map-based cloning of the *mj* locus. Initial mapping placed the *mj* locus onto cucumber chromosome 1. Screening of a large backcross population with over 1,200 individuals identified 18 recombinants using two markers flanking the *mj* locus. By phenotyping and genotyping these recombinants, we were able to narrow down the candidate gene region into a 13 kb physical interval that contained only one annotated gene which was predicted to encode Systemic Acquired Resistance Deficient 1 (SARD1). SARD1 is a calmodulin-binding transcription factor that has been shown to be a master regulator of host defense responses. Importantly, there were no differences in the protein-coding sequence of SARD1 between JRKN resistant and susceptible *C. sativus* genotypes. The only sequence variants identified were within the promoter region of this gene, indicating that differences in this gene’s expression are responsible for the *mj* resistant phenotype. To compare the growth of JRKN in resistant and susceptible genotypes, we conducted a time course development experiment using acid fuchsin staining as well as histological comparisons of the nematode and its feeding site. Our results indicate that *mj* induces resistance later than many other nematode resistance genes with differences in JRKN development only becoming visible eight days post-inoculation. Taken together, our results suggest that the *mj* resistance trait is controlled by a unique loss-of-susceptibility mechanism that likely involves the expression of the SARD1 gene in cucumber. Further research into the mechanism of SARD1-based resistance to JRKN could provide valuable information on how this nematode manipulates host defenses, and potentially allow this resistance mechanism to be utilized in other host crops and against other RKN species.

## Sugar beet mediated belowground interactions between root-knot and root-lesion nematodes

Sadic, Busra and E. Lewis

University of Idaho, Department of Entomology, Plant pathology and Nematology, Moscow, ID 83844, USA

### Abstract

Plant parasitic nematodes (PPNs) are an important group of root feeders that regularly damage a range of crops. While CO_2_ is a major attractant for PPNs in the rhizosphere, small-molecule signaling is crucial in the interactions between host plants and nematodes and between multiple species of nematodes. Plant exometabolome and root exudates regulate PPN development and behavior whereas nematode pheromones, known as ascarosides, trigger plant defense metabolic pathways. Ascarosides play a central role in nematode chemical communication, regulating various aspects of nematode development and behavior. However, the specific signals, cues and mechanisms involved in these belowground interactions remain poorly understood. We investigated sugar beet (*Beta vulgaris* subsp. *vulgaris*) mediated interactions between two PPN species (*Meloidogyne incognita*, *Pratylenchus neglectus*) to test effects of plant root exometabolome (nonvolatiles) on PPNs. We collected root exudates at 1, 2 and 7 days exposure. Root exudates were collected from PPN-exposed and non-PPN exposed sugar beets grown hydroponically. Additionally, at 1, 2 and 7 days, exometabolome of conspecific and heterospecific PPNs without a host plant was measured. Two experiments were conducted. First, two-way choice tests were conducted in petri dishes using root exudates collected from infected and uninfected plants as cues. In the second experiment, glass olfactometers filled with sand were used to assess effects of plant exudates on PPNs behavior. The study revealed that PPNs preference varied with plant infection status. By focusing on both plant-mediated interactions and nematode-nematode interactions, our study provides valuable insights into the chemical ecology of plant-nematode and nematode-nematode interactions for future research.

## Effects of anaerobic soil disinfestation on root-knot nematodes in greenhouse in North Carolina

Sanabria-Velazquez, Andres D.^1^, A. Gorny^1^, T. Adhikari^1^ and F. Louws^1,2^

^1^Department of Entomology and Plant Pathology, North Carolina State University, 1575 Varsity Drive, VRB Module 6, Raleigh, NC 27695

^2^Department of Horticultural Science, North Carolina State University, 118 Kilgore Hall Raleigh, NC, 27695-7609

### Abstract

Anaerobic soil disinfestation (ASD) involves adding easily biodegradable sources to the soil, which are then consumed by soil microorganisms, leading to anaerobic conditions that reduce the populations of harmful soilborne pathogens. To effectively implement ASD in horticultural systems, local sources of carbon that are accessible to farmers are necessary. This study aimed to find alternative methods to the use of fumigants for pre-plant treatment by using ASD to control plant-parasitic nematodes and improve soil health. We tested five organic amendments, including spent brewery grain, mycelia (both fresh and composted), tobacco waste, biochar, and sweet potato waste from local industries, at a rate of 20.2 Mg/ha in greenhouse trials with ten replicates of each treatment. Control treatments either remained uncovered (aerobic control) or were covered with plastic mulch (anaerobic control). In each pot, the soil was combined with one of the available carbon sources, and a mixture of *Meloidogyne hapla* containing 1,000 eggs was added and thoroughly mixed. To avoid the exchange of gases, the pots were covered with plastic mulch to limit air gas exchange and incubated for four weeks. After the ASD process, tomato seedlings were planted, and their growth was monitored for 40 days. The study found that the use of ASD with these carbon sources significantly reduced root-knot nematode galling in tomato plants (*P* < 0.001). There were no *M. hapla* galls found on the roots of plants that were cultivated in any of the ASD-treated soils except for those amended with biochar. ASD, using these carbon sources or in combination with other inexpensive soil amendments available throughout the season, could be a sustainable alternative for managing root-knot nematodes in tomato fields in North Carolina.

## Southern root-knot nematode development in newly identified MG4 resistant soybeans

Santos Rezende, Josielle and T. T. Watson

Department of Plant Pathology & Crop Physiology, Louisiana State University Agricultural Center, Baton Rouge. LA 70803

### Abstract

*Meloidogyne incognita* is an important plant parasitic nematode with a wide host range and distribution in the United States. Also known as the Southern root-knot nematode (SRKN), *M. incognita* causes significant yield losses in a variety of crops in the U.S., including soybeans. Soybean can be drastically damaged by SRKN and this is a threat to farmers relying on the existing limited chemical and cultural management options for this nematode. Resistance is the most efficient and economical approach to control *M. incognita*. However, resistance to SRKN is very limited in Maturity Group 4 (MG4) commercial soybean varieties which have been planted in more than 15M acres each year. Additionally, the resistance mechanism involving *M. incognita* and soybeans is largely unknown. The objective of this study was to characterize soybean resistance mechanisms toward SRKN in newly identified MG4 genotypes containing novel quantitative trait loci for resistance. We therefore conducted a time course study to evaluate nematode development in 3 newly identified MG4 resistant soybeans. Soybean genotypes, including one resistant and two susceptible controls, were grown under greenhouse conditions in plastic pots filled with sterilized and fertilized sand arranged in a randomized complete block design, with four replicates per genotype on each sampling date. Samples were collected 4, 7, 14, and 21 days after inoculation (dai) with 2,000 second-stage juvenile (J2) SRKN upon cotyledon emergence. Washed root systems were cleared in dilute sodium hypochlorite and the number of nematodes representing each developmental stage (J2, third- and fourth-stage juveniles (J3/J4), and adult female) were quantified in whole root segments stained with acid fuchsin. Only J2 SRKN was observed inside the roots at 4 dai. By 7 dai, J3/J4 were detected in the susceptible genotypes and at the 14 dai time point, J3/J4 were observed in susceptible and resistant genotypes with females appearing at 21 dai in all genotypes examined. The total number of J3/J4 and adult females was greater for the susceptible genotypes than the resistant soybeans. Overall, our preliminary results suggest that newly identified MG4 resistant genotypes may delay nematode development as a possible resistance mechanism toward *M. incognita*.

## Winter cover crops and biological products to manage *Meloidogyne incognita* and promote soil health in sweetpotato

Schloemer, Claire^1^, K. S. Lawrence^1^, S. H. Graham^1^, K. -H. Wang^2^ and B. Sipes^2^

^1^Auburn University, Auburn, AL

^2^University of Hawai’i at Manoa, Honolulu, HI

### Abstract

Organic production is increasing across the Southeast, but there is a need to develop effective organic integrated nematode management practices for sweetpotatoes. To address this, field trials were established in Brewton, AL in a Benndale fine sandy loam soil and Dobson, NC in a Fairview cobbly sandy clay loam to determine the effect of selected winter cover crops and biological products in the suppression of nematode and insect pests. This was accomplished by planting sweetpotatoes into the footprints of selected winter cover crops. Entomopathogenic nematodes (*Steinernema feltiae*, *S. carpocapsae*, and *Heterorhabditis bacteriophora*), insect pathogen *Beauveria bassiana,* and OMRI bionematicide Majestene were applied to half of each plot to determine their combined ability to suppress pests. Ultimately, the cover crop mix of crimson clover, daikon radish, elbon rye, and wheat was associated with high marketable yields (+ 2000 lb/A over fallow), low insect damage, and lower *M. incognita* populations. The three way mix of biological products numerically increased marketable yields in both locations and significantly reduced internal *M. incognita* damage in Alabama. In Alabama, the legume winter cover crops crimson clover and field peas supported higher *M. incognita* populations at sweetpotato planting with 97 to 368 J2/100 cm^3^ soil, respectively. The remaining cover crops supported from 1–78 J2/100 cm^3^ soil. Total *M. incognita* populations through the season across 4 sampling dates increased on crimson clover to 454 and field peas to 863 J2/100 cm^3^ soil, and the lowest population of 345 J2/100 cm^3^ soil was observed following daikon radish. North Carolina populations were lower at sweetpotato planting ranging from 28 to 72 J2/100 cm^3^ soil for fallow and crimson clover. Total *M. incognita* populations across the season with 4 sampling dates were significantly lower on elbon rye, wheat, and mix compared to crimson clover. All cover crops in North Carolina increased free-living nematodes compared to the fallow, and across cover crops in both locations, bacterivores were most numerous followed by fungivores and predators. CO_2_ respiration increased over the season, and in Alabama the highest respiration was recorded near harvest. In North Carolina, the highest CO_2_ respiration was observed in the cover crop mix and lowest in the fallow. PLFA soil health values found total living microbial biomass ranged from 14,076 to 16,105 ng/g in North Carolina but was lower in Alabama ranging from 2,925 to 3,495 ng/g. Soil textural differences between locations likely contributed to these differences. Organic sweetpotato production is challenging, however the North Carolina cover crop mix was associated with higher yield, lower *M. incognita* populations, high CO_2_ respiration, and higher total living microbial biomass. Overall, the biological control products significantly reduced internal *M. incognita* damage in Alabama and numerically increased yields across both locations.

## Strategies for a digital nematode anatomy database

Schroeder, Nathan E.^1^, H. Imker^1^, A. Kenfield^1^, S. Luke^1^ and D. H. Hall^2^

^1^University of Illinois at Urbana-Champaign, Urbana, IL 61801

^2^Albert Einstein College of Medicine, Bronx NY 10461

### Abstract

Digital micrographs are essential data for many scientific investigations. Funding agencies and journals frequently require data management plans and availability statements on how data will be maintained long-term and shared with other researchers. The WormImage electron micrograph repository includes over 80,000 micrographs that were digitized from prints and negatives of serial-sections collected over multiple years. The majority of these data are of *Caenorhabditis elegans* and were originally collected at the Medical Research Council in the United Kingdom during the 1970s and 80s. This workshop presentation will explore facets of the development, continued maintenance, and growth of this database including digitization parameters, strategies for online sharing, and metadata organization.

## Toxic effects of the trap crop, *Solanum sisymbriifolium*, on the pale cyst nematode, *Globodera pallida*

Schulz, Lindsay^1^, L. M. Dandurand^1^ and I. Popova^2^

^1^Department of Entomology, Plant Pathology, and Nematology, University of Idaho, Moscow, ID 83844-2329

^2^Deparment of Soil Science, University of Wisconsin-Madison, Madison, WI 53706-1299

### Abstract

*Globodera pallida*, a potato cyst nematode, is a quarantined pest of potato that was first found in Idaho in 2006. Containment and eradication of this economically devastating pest of potato has been the focus of control since its’ discovery. *Globodera pallida* survives for 30+ years in the soil, can cause up to 80% potato yield loss on susceptible potato varieties, and is readily spread by infested soil. Soil fumigation has been the key effort in eradication. New methods for controlling *G. pallida* are essential for the success of the eradication program due to the banning of many nematicides. *Solanum sisymbriifolium*, commonly called litchi tomato or sticky nightshade induces hatch but limits reproduction of *G. pallida* and can be used as an alternative control measure. However, because *S. sisymbriifolium* has little economic value as a crop and seeds are largely unavailable, it has not been widely adopted for use by producers in Idaho. There is evidence that this plant kills the nematode through production of toxins although this is poorly understood. Previous research indicates that pure solanaceous glycoalkaloids may be toxic to *G. pallida* with glycoalkaloids reducing hatch, infection, and reproduction of *G. pallida* by 87%, 94%, and 99% respectively. Currently, our research indicates that glycoalkaloids are found in higher concentrations in the leaf tissue rather than stems or roots. Liquid-liquid extraction of *S. sisymbriifolium* leaf and stem tissue by hexane and 1-butanol reduced hatch by 65% and 72%, respectively. These extracts will be further fractionated by HPLC. *Solanum sisymbriifolium* plants that are infected with *G. pallida* will also be evaluated. Once fractionated, their impacts on *G. pallida* hatch and reproduction will continue to be evaluated. Potential discovery of novel chemistries for nematicide development would be a valuable achievement for Idaho producers, or anyone dealing with *G. pallida* infestations.

## Does mixing different soybean varieties improve yield and reduce soybean cyst nematode in no-till fields?

Schumacher, Lesley

USDA, Agricultural Research Service, Crop Genetics Research Unit, Jackson, TN 38301

### Abstract

Soybean cyst nematode (*Heterodera glycines*, SCN) is a major limiting factor of worldwide soybean production. Resistant varieties stemming from PI 88788 are the main form of management, but their overuse has led to a loss of efficacy. Innovative approaches to extending resistance genes are needed. Therefore, the aim of this study was to assess the utility of mixing SCN-resistant and susceptible varieties for SCN management. The study was conducted in west Tennessee at two no-till sites naturally infested with SCN HG Type 1.2.5.7 during the 2021 and 2022 seasons. Site 1 had low (less than 1000 SCN/100 cm^3^ soil) and site 2 had high (>1000 SCN/100 cm^3^ soil) SCN pressure upon initial soil sampling. Combinations of commercial maturity group V soybean varieties were counted using a seed sorter (250 seeds total per row) and replicated four times in a randomized complete block design. There were seven treatments (variety sources): 1) 100% susceptible; 2) 100% PI 88788; 3) 100% Peking; 4) 50% susceptible and 50% PI 88788; 5) 50% susceptible and 50% Peking; 6) 50% PI 88788 and 50% Peking; and 7) 33% susceptible, 33% PI 88788, and 33% Peking. The response variables analyzed were SCN counts (cysts, eggs per cyst) at planting and at harvesting, and yield. Soil samples were collected using a standard soil probe and nematodes extracted via an elutriator followed by sucrose-centrifugation. Data were analyzed using R version 4.0.3 and normality of residuals and homogeneity of variances checked before being submitted to one-way ANOVA, with means separated using Tukey’s HSD. There was a significant (*P* < 0.05) effect of year, so data were analyzed separately for 2021 and 2022. There were no significant treatment effects on cyst number at either site in both years. There were no differences in fecundity (eggs/cyst) at site 1 in either year. However, fecundity was significantly greater in the susceptible/Peking treatment (227 eggs/cyst) than in Peking-only (39 eggs/cyst) at site 2 in 2021. Plots with the three-way mix had greater fecundity (147 eggs/cyst) than the PI 88788/Peking treatment (24 eggs/cyst) in site 2 in 2022. In both years, there were no significant differences in yield between the treatments at site 1, nor in 2022 at site 2. However, at site 2 in 2021, yield was greatest in the Peking-only (3426 kg/ha) and least in susceptible-only (2,422 kg/ha) plots. 2022 was a particularly dry year, so nematode populations increased, and resistance did not improve yield. SCN reproduction occurred on both resistant varieties, indicating that once an aggressive HG Type already exists in the field, no significant reductions in SCN population densities using resistant varieties, nor yield advantages using resistant varieties, may be observed. The variety mixing approach may work better in a field with a less aggressive SCN population, or using varieties derived from PI 437654.

## Unraveling the virulence mechanism of *Meloidogyne hapla* through genomics and functional genetics

Shakya Pallavi^1^, A. Coomer^1^, S. van de Ruitenbeek^3^, M. Maulana^3^, V. M. Williamson^1^, M. Sterken^3^ and S. Siddique^2^

^1^Department of Plant Pathology, University of California Davis, One Shields Avenue, Davis, CA, 95616, USA

^2^Department of Entomology and Nematology, University of California Davis, One Shields Avenue, Davis, CA, 95616, USA

^3^Laboratory of Nematology, Wageningen University & Research, Droevendaalsesteeg 1, 6708 PB, Wageningen, The Netherlands

### Abstract

Root-knot nematodes (RKNs) belonging to the genus *Meloidogyne* are notorious for causing severe damage to plants. They are able to parasitize nearly all vascular plants and alter plant development, immunity, and physiology through the secretion of effector proteins from their gland cells. *Meloidogyne hapla* is a facultative meiotic parthenogenetic species reproducing sexually in the presence of males but asexually in their absence. This unique mode of reproduction facilitates the production of recombinant inbred lines between virulent and avirulent strains, enabling the segregation of virulence genes. Our previous study utilized two strains of *M. hapla*, VW9 and LM, which differ in their ability to infect a common bean cultivar called NemaSnap. Using a genetic map based on DNA polymorphisms, quantitative trait locus analysis revealed that a single major locus, NemaSnap Virulence (NSV), is responsible for the difference in *M. hapla’s* ability to reproduce on NemaSnap. However, due to limitations in the current reference genome, the genes in this locus remain uncharacterized. Here, we utilized PacBio’s long-read sequencing technology HiFi to assemble a contiguous genome of *M. hapla* strain VW9, spanning 60.8 Mbp. The genome is currently being annotated using Iso-Seq data generated from three life stages (eggs, second-stage juvenile and females). We aim to use this newly constructed reference genome to identify and functionally characterize genes located in the NSV locus, which will contribute to our understanding of the mechanism underlying nematode virulence Furthermore, the successful completion of *M. hapla* genome assembly will provide a valuable resource to the nematology community, enabling new strategies to combat RKNs.

## A sustainable grower-based method for entomopathogenic nematode production

Shapiro-Ilan, David^1^, C. Oliveira-Hofman^1,2^ and S. Steffan^3^

^1^USDA-ARS Southeastern Fruit and Tree Nut Research Station, Byron, GA 31008

^2^Bayer Crop Science, Chesterfield, MO 63017

^3^USDA-ARS, Vegetable Crops Research Unit Madison, WI 53706

### Abstract

Entomopathogenic nematodes in the genera *Steinernema* and *Heterorhabditis*, produced through *in-vitro* or *in-vivo* methods, are effective insect biological control agents. *In vivo* production yields good quality nematodes, but the costs associated with obtaining insects and labor make this production system have a low economy of scale. Conceivably, if growers can produce their own nematodes, then costs could be reduced. Grower-based production systems described to-date are not sustainable because they rely on outside sources to obtain or calibrate inoculum. Here, we describe a self-sufficient grower-based system where growers can produce in-house nematodes after obtaining the initial inoculum from a reliable source. We validated our approach in two experiments comparing *in-vivo* nematode production from standard White traps and a grower-based approach using polyacrylamide gel. For both tested species, *Steinernema carpocapsae* (Weiser) and *Heterorhabditis bacteriophora* Poinar, the grower-based approach produced equal to or more nematodes than the standard method. For example, when comparing the average yield of *S. carpocapsae* infective juveniles (IJs) per *Galleria mellonella* cadaver (n=30), the standard White trap method produced 159,114 ± 9,669 IJs, whereas the grower-based approach produced 244,029 ± 16,241 IJs. The sustainable system described herein has promise for wide adoption by growers.

## Molecular and morphological characterization of *Boleodorus volutus* from medicinal hemp in Maryland

Skantar, Andrea M.^1^, Z. A. Handoo^1^, M. R. Kantor^2^, M. N. Hult^1^, S. Li^1^ and L. K. Carta^1^

^1^Mycology and Nematology Genetic Diversity and Biology Laboratory, USDA-ARS-NEA-BA, Beltsville, MD 20705

^2^Department of Plant Pathology and Environmental Microbiology, Pennsylvania State University, University Park, PA, 16802

### Abstract

Medicinal hemp (*Cannabis sativa indica*) was recently deregulated for cultivation in the United States. A survey was conducted in October and November, 2019 from an organic hemp field in Baltimore County, Maryland that had been previously planted with strawberries. One of the plant-parasitic nematode isolates from that field that was characterized by morphological and molecular methods contained anatomical features and measurements that fit within ranges previously reported for *Boleodorus volutus*. Sequences that were obtained from the specimens included SSU 18S and LSU 28S rDNA, further expanding the sparse sequence representation of these genera. Phylogenetic analysis of SSU placed the *Boleodorus sp*. isolate into a polyphyletic clade with *B. thylactis* and *B. volutus*, but support was relatively poor for individual branches. BlastN of the LSU fragment against available sequences in GenBank strongly matched other *B. volutus* and one unnamed isolate. Phylogenetic trees based on alignment of 28S rDNA placed the isolate with other *B. volutus* sequences, and separation from the *B. thylactus* clade was well supported. Based on combined morphometric and molecular data, this isolate was identified as *Boleodorus volutus*, representing a new record for the United States.

## Survival and infectivity of entomopathogenic nematodes from desiccated living bombs

Silvester, N. P. and B. S. Sipes

Department of Plant and Environmental Protection Sciences, University of Hawai'i at Manoa, Honolulu, HI 96822

### Abstract

Entomopathogenic nematodes (EPNs) are usually applied as foliar sprays or via irrigation. These methods, however, leave EPNs exposed to UV radiation and desiccation. Living bombs are a relatively novel method in which EPNs are applied while within an insect host cadaver. The cadaver protects the EPNs from UV radiation and desiccation. Living bombs can be difficult to handle, consequently, formulating an EPN carrier into a dry cadaver would be beneficial. A laboratory experiment was conducted in which *Galleria mellonella* cadavers infected by *Heterorhabditis indica* were chemically desiccated with KCl. EPN-infected cadavers were incubated for 7 days before transfer into desiccation chambers filled with a saturated solution of KCL, providing 85% relative humidity, for 21 days. Cadavers were weighed before and after desiccation. Desiccated cadavers were placed into White traps after desiccation and infective juveniles (IJs) collected after 48 hrs. To examine viability of EPNs after desiccation treatment, Lab cultured *G. mellonella* larvae were exposed to desiccated or non-desiccated *H. indica* IJs in a petri dish and larval death recorded after 48 hrs. The experiment was repeated twice with 30 replications for each repeat. Cadavers averaged over a 40% loss in weight after desiccation. Desiccated cadavers were leather-like and easier to handle without fear of rupture as compared to the non-desiccated cadavers. The desiccated cadavers produced IJs equal in number to that of non-desiccated cadavers with both averaging over 200,000 IJs/cadaver. The IJ emerging from desiccated and non-desiccated cadavers infected and killed 100% of larvae of *G. mellonella* that were exposed. EPN infectivity and effectiveness were not impacted by desiccating the host cadavers. Subsequently, desiccated cadavers will be evaluated in field trials to evaluate the efficacy of living bombs as an EPN application method in a sweet potato field infested with sweet potato weevils (*Cylas formicarius*).

## Anaerobic soil disinfestation as a non-chemical tool for the management of nematodes and weeds in Sweetpotato

Singh, Simardeep^1^, M. Cutulle^1^, C. Khanal^1^, W. Rutter^2^ and P. Wadl^2^

^1^Clemson University, Department of Plant and Environmental Sciences, SC, 29634

^2^USDA-ARS Charleston, SC 29414

### Abstract

Nematodes and weeds have become increasing issues for the sweet potato growers because of the lack of resistance and reduced efficacy of nematicides and lack of registered herbicides in sweetpoato. Anaerobic soil disinfestation (ASD) has the potential to fit into current pest management practices as an alternative to pesticides. ASD can be achieved by the incorporation of carbon source followed by irrigating to field capacity and covering the soil with plastic mulch. Greenhouse studies were conducted at Clemson University to specifically look at the impact of carbon source and soil type on cumulative anaerobicity, nematode population, weed control, and sweet potato vigor. Nineteen-litre pots were filled with three different soil types (Charleston-loamy/native, Blackville-coarse high sand content, and Clemson-high clay content) and were mixed with cottonseed meal (CSM) or no carbon amendment. Each pot was inoculated with guava root-knot nematode. Tiff film was then sealed over the pots for 6 weeks. Sweet potato slips (Bayou Belle) were transplanted. Throughout the study, cumulative anaerobicity, weed counts, sweet potato vigor, weed biomass, and nematode counts were taken. Results suggest that the plots receiving the carbon amendment spent the most time under anaerobic conditions. Sweet potatoes grown in non-carbon source-treated pots (26.94%) had significantly higher gall percentages than CSM-treated pots (8.33%). Experimental units that went anaerobic (< 200 mv) generally had fewer nematodes (8 J2/100 cm^3^ soil) and weeds (54.65%) when compared to plots that did not go anaerobic (>200 mv). The addition of cotton seed meal as a carbon source resulted in an increase in the root biomass of sweet potato (59.27%). Overall, this study will improve our understanding of the use of anaerobic soil disinfestation for the management of root-knot nematode and weed infestation in sweet potato.

## Genetic data reveals two new haplotype groups of the *Xiphinema americum*-group from native tall prairies of Nebraska and Kansas

Sirengo, David, T. Harris, K. Powers, B. Higgins, P. Mullin and T. Powers

Department of Plant Pathology, University of Nebraska-Lincoln, NE 68583

### Abstract

Plant ectoparasitic nematodes from the genus *Xiphinema* infect a wide range of plant species within native grasslands, forests, orchards, vineyards, and annually cultivated crops. *Xiphinema* has a worldwide distribution and species are challenging to identify due to their subtle morphological differences and overlapping morphometric characters between species. Taxonomically they are often classified within two distinct groups: *Xiphinema* non-*americanum* and the *Xiphinema americanum* complex. The former includes over 200 species, whereas the latter encompasses approximately 61 species characterized by the presence of endosymbiont bacteria. Studies have also noted congruence between bacterial endosymbiont phylogeny and the phylogeny of their nematode hosts. Some, but not all species of both groups vector plant viruses that can cause significant damage to a diversity of crops. Owing to their ability to transmit viruses, the *X*. *americanum*-group is subject to regulation as pests in North America and classified as quarantine pests in the European Union. Evidence indicates that not all *X. americanum* species are virus vectors and presently, only seven species have been documented with the ability to transmit viruses. In an effort to establish a more comprehensive analysis of *X. americanum* in North America, we have initiated a mitochondrion cytochrome c oxidase 1 (COI) barcode characterization of isolates from native plant communities and agricultural sites. To date, a total of 222 sequences, consisting of 144 de novo sequences and 78 sequences from GenBank have been subjected to phylogenetic analysis. Most notable on the phylogenetic tree are two well-supported clades of 55 and 18 specimens respectively, that are comprised almost exclusively of *Xiphinema* from tallgrass prairies. The larger clade incudes specimen collected from five different remnant prairies in eastern Nebraska and Kansas. The smaller clade of prairie specimens includes a single corn-soybean production field indicating possible adaptation to soils cultivated annually. Shared haplotypes across different states and different agricultural production systems indicate that some *Xiphinema* isolates are polyphagous and widespread in North American soils.

## Relationship between soybean cyst nematode and soil texture in Ohio

Small, Ambria, T. Ralston, S. Mondal and H. D. Lopez-Nicora

The Ohio State University, Dept. of Plant Pathology, Columbus, OH 43210

### Abstract

Soybean cyst nematode (SCN), *Heterodera glycines,* is the most economically important pathogen of soybean in North America, resulting in approximately 30% yield losses without observable aboveground symptoms. It is not well understood how SCN populations change throughout the growing season in Ohio under different soil types. Soil types that support the majority of Ohio soybean crops are medium-textured, silty to clay soils due to historical glacial movement and the pre-existing parent materials. The objectives of this study were to evaluate SCN reproduction factor (RF = final population/initial population) in commercial soybean fields in Ohio and evaluate the relationship between SCN RF and soil texture. During the 2022 growing season, soybean growers and extension educators submitted thirty-two soil samples from across Ohio to the Soybean Pathology and Nematology Laboratory at Ohio State University for SCN quantification. The twelve soil regions of Ohio, described in the STATSGO database for the Natural Resources Conservation Service, were used to assess the relationship between SCN RF and soil texture. Five of the twelve soil regions were represented by the soil samples submitted for SCN count. An increase in SCN population with a RF ranging from 1.6 to 51.6, was observed in 38% (n = 14) of the samples. A decrease in population was detected in 34% (n = 11) of samples. Of these decreasing populations, 82% decreased to undetectable levels. An increase in SCN population was detected in 67% sand-prominent samples. Changes in population density found in sandy soils were significantly (*P* < 0.05) greater than in other soil types, such as soil regions containing high silt and clay composition. Samples composed of silt and clay soils represented 87% of the sites where SCN populations increased during the 2022 growing season. To better understand the relationship between SCN RF and soil texture in Ohio, particle analysis will be completed to determine precise soil texture of soil samples. Additionally, soil health analyses will be completed to determine parameters potentially associated to SCN RF (e.g., soil pH, CEC, soil respiration, wet aggregate stability, organic carbon content, macro-/micro-nutrient content, etc.). This study is currently being repeated in 2023 growing season to further evaluate SCN RF trends and their relationship with soil texture, soil health, and crop rotation in Ohio commercial soybean fields.

## Literature review of nematicidal bacteria helps to identify new strains to screen with multiple potential modes of action

Steger, Callie and J. P. Jensen

AgBiome - 104 TW Alexander Drive, Bldg 1, Research Triangle Park, NC 27709

### Abstract

Biological nematicides are becoming an important tool for nematode management due to their ability to deliver multiple modes of actions and favorable regulatory profiles. To understand the diversity and abundance of nematicidal bacteria, we conducted a thorough literature review across 129 articles where researchers have documented the nematicidal potential of 287 bacterial strains containing 101 unique proteins and 94 unique metabolites. When provided in the articles, we recorded genomic sequences pertaining to each strain’s 16s, proteins of interest, or biosynthetic gene clusters. AgBiome maintains a large bacterial collection with over 100,000 isolates with fully-sequenced genomes. Using our proprietary bioinformatic tools, we compared the literature-identified genomic sequences against our collection to highlight strains with relative sequence identity, limiting results to the top 5 hits per search term. Using the search term of 16s sequence, we identified 242 similar strains in our collection. Using the search term of gene sequences of proteins of interest, we found 329 strains that contain at least one gene of interest in our collection. We are continuing to identify biosynthetic gene clusters relating to the literature-identified metabolites, and will use those to search against our collection. With all of this information, we will conduct a highly targeted screening study to characterize the nematicidal activity of strains with multiple predicted modes of action, and identify new strains with strong product potential. Such targeted screening is a great opportunity to complement our ongoing empirical screening efforts, allowing us to efficiently discover and develop new biological nematicides.

## Nondestructive assessment of *Rotylenchulus reniformis* resistance in *Gossypium* spp.

Stetina, Salliana R and J. E. Erpelding

USDA Agricultural Research Service, Crop Genetics Research Unit, P.O. Box 345, Stoneville, MS 38776 USA

### Abstract

Nondestructive sampling to phenotype cotton (*Gossypium* spp.) plants for resistance to reniform nematode (*Rotylenchulus reniformis*) in early breeding generations would be highly useful for introgression of nematode resistance from exotic germplasm resources. Based on the number of females infecting the roots, a nondestructive method was successfully developed to assess host plant resistance to the reniform nematode. In the first set of experiments, the root system was removed at either 0, 1, 2.5, or 5 cm below the soil line. The number of females infecting the removed portion of the root system was counted, and it was determined that resistant lines could be identified while leaving up to 5 cm of roots with the shoot. In the next set of experiments, the rate of plant recovery and reproductive development was evaluated using a combination of shoot retention (all leaves, no leaves, leaves at bottom two nodes, or leaves at top two nodes) and root retention (all root, 2.5 cm, 1 cm, or 0 cm root) treatments. The treatment combination in which 2.5 cm roots and the top leaves were kept was the one in which plants recovered the quickest, and which performed comparably to the treatment in which neither roots nor shoots were modified. Even after modification to shoots and roots, all plants were able to produce flowers, fruits, and seeds of the next generation. This screening method can be used by plant breeders to identify resistant lines in early generations while still allowing the plant to produce seeds of the next generation.

## Interception of root lesion nematodes, *Pratylenchus bolivianus* (Nematoda, Pratylenchidae), on the Alstroemeria sp. plants from the Netherlands

Sun, Fengcheng^1^, C. Grenier^1^, B. Pearce^1^, R. Alfeche^1^ and Q. Yu^2^

^1^Canadian Food Inspection Agency, 3851 Fallowfield Road, Ottawa, ON, K2H 8P9, Canada

^2^Ottawa Research and Development Center, Agriculture and Agri-Food Canada, Ottawa, ON K1A 0C6, Canada

### Abstract

The root-lesion nematode *Pratylenchus bolivianus* Corbett, 1983, is an important pest of ornamental and crop plants including Inca lily (*Alstroemeria* spp.), Sword fern (*Nephrolepis exaltata (L.)* Schott. Stiff.), rooibos (*Aspalathus linearis* (Burm. f.) Dahlgren; oats, potato, tomato and carnation. On Alstroemeria, a prolonged infestation of *P. bolivianus* can result in a reduction in flower production and root size. In 2022, root-lesion nematodes were detected from samples of Alstroemeria plants collected from a shipment imported to Canada from the Netherlands under a routine import inspection by the Canadian Food Inspection Agency (CFIA). The nematodes were identified as *P. bolivianus* based on the morphological characters and morphometric characteristics of females: three distinct lip annuli; rounded tail terminus either smooth or striated; stylet length = 17.7 (17 to 18.7) μm; L = 642.8 (557 to 697) μm; a = 23.6 (21.6 to 25.7); V = 80.9% (75.8% to 82.29%); PUS = 26 (20 to 36.77) μm; tail length = 27.59 (19 to 32.4) μm; c’ = 1.5 (1.2 to 1.7); spermatheca inconspicuous, not filled with sperms and males absent, indicating that the population belongs to the parthenogenetic morphotype. The sequence of D2-D3 expansion segment of 28S rRNA gene from this population revealed over 99% identity with published DNA sequences of the same genomic region for *P. bolivianus* populations from South Africa, Bolivia, United Kingdom and Colombia. Alstroemeria is a popular cut flower crop worldwide. In Canada, importing the plants for planting is subject to plant phytosanitary regulations (ISPM No. 36 and CFIA Plant Health Directive D-08-04 and D-96-20). According to the regulations, the production of plants to be exported should be operated under a clean stock program in the exporting country in order to prevent introduction, spread and establishment of harmful organisms. The potential establishment and plant health risk of *P. bolivianus* in Canada still needs to be assessed. However, the Dutch production facility has been advised to use pest-free stocks.

## Biology and genome of the beech leaf disease nematode, *Litylenchus crenatae* subsp. *Mccannii*

Taylor, Christopher G., K. Scott, T. Ross, C. Chagas de Freita, J. Slot, P. Bonello and M. Roth

The Department of Plant Pathology, The Ohio State University, Columbus and Wooster, OH

### Abstract

Beech leaf disease (a.k.a BLD) represents a new threat to American beech trees (*Fagus grandifolia* Ehrh.) in forest ecosystems in the Eastern United States. Infected trees show, progressively, “banding” and “crinkling” symptoms on leaves in the early spring, followed by leaf senescence that occurs sporadically throughout the summer and into fall. Multi-year infections of the same tree can lead to branch dieback, and eventually tree mortality. Our team has corroborated findings by other researchers that suggests an association of the nematode *Litylenchus crenatae* subsp. *mccannii* is connected to symptomatic leaves. However, to date we cannot rule out the possibility that a microbial component may also be contributing to the lethal aspects of this disease. Little is known about the biological, developmental, and molecular progression of this disease and the various factors that contribute to its rapid spread throughout the Northeastern United States. To elucidate the biology/pathology of this disease, an extraction method was developed that allows for the isolation of large numbers of nematodes from infested leaves. We have created a high-quality draft genome assembly for *Litylenchus crenatae* subsp. *mccannii* using DNA extracted from harvested nematodes and a combination of MinION and Illumina sequence data. The assembly is 105.4Mb in size and is made up of 266 contigs. Insights from the annotated genome will provide clues to help us understand the biology and pathology related to this disease and its relations to other plant- and fungal-parasitic nematodes.

## Reklemel™ active (fluazaindolizine, salibro™) a new nematicide for the management of key plant-parasitic nematodes in multiple crops

Tewari, Sunil and T. Thoden

Corteva Agriscience, 9330 Zionsville Rd., Indianapolis, IN 46268, USA

### Abstract

Reklemel™ active (fluazaindolizine, Salibro™) is a novel, non-fumigant, chemical nematicide discovered and being developed by Corteva Agriscience for the control of key plant-parasitic nematodes infesting a wide range of annual and perennial crop groups in North America including tree nuts and tree fruits as well as fruiting vegetables, cucurbits, root and tuber vegetables, stone fruits, and grapes, among others. Reklemel is the first sulfonamide nematicide, has a unique mode of action (MoA), a favorable environmental and toxicological profile and can be applied in a variety of methods and timings. It has demonstrated selective and effective control of key nematode pests including a wide range of root-knot species (*Meloidogyne* spp.) as well as some root-lesion nematodes (*Pratylenchus* spp.), among others. The discovery, development, and characterization of this chemistry, by Corteva Agriscience, comprises over 10 years of extensive research at the global level encompassing thousands of laboratory studies, greenhouse, and field trials. This work has defined key attributes of this novel chemistry including, nematode activity, soil behavior, and soil health compatibility. A US-focused summary of the key insights gained from the development of Reklemel™ as well as label and product registration updates will be presented.

## Biological-based pest management of spotted wing drosophila, *Drosophila suzukii* using insect pathogenic nematodes and fungi

Thapa, Rambika, M. Usman, M. Quintanilla-Tornel and R. Isaacs

Department of Entomology, Michigan State University, East Lansing, 48823, MI

### Abstract

Spotted-wing drosophila (SWD) *Drosophila suzukii* is an important pest of soft-skinned fruits native to Southeast Asia. Since its first detection in the continental U.S. in around 2008 it has spread to most parts of the country causing a yearly loss of around $500 million. Due to the different problems associated with the use of conventional insecticides, it is better to identify an alternative approach to manage the SWD in a sustainable manner. Biological-based pest management using insect-pathogenic nematodes and fungi could be a promising alternative to conventional chemical insecticides. The objective of this study is to screen the entomopathogenic nematodes (EPNs) and fungi (EPFs) against the soil-dwelling stages of SWD. Last year we tested the pathogenic potential of three EPNs including *Steinernema carpocapsae*, *S. feltiae,* and *Heterorhabditis bacteriophora* against soil-dwelling stages of SWD and we found very promising results. So, we have decided to test a broad array of EPNs including *S. riobrave*, *S. glaseri*, *S. rarum*, *H. georgiana*, *H. megidis*, *H. floridensis,* and *H. indica* that have never been tested against SWD. Along with EPNs, we have a plan to test the different species/strains of EPF including *Beauveria bassiana*, *Metarhizium brunneum*, *Cordyceps javanica,* and *C. fumosorosae* against the soil-dwelling stages of SWD. Literature is scarce on the efficacy of EPNs and EPF against SWD. To the best of our knowledge, this will be the first time these EPFs are tested against the soil-dwelling stages of SWD. We are seeking potential synergy between the microbial agents. This study will help us find information on the possibility of using EPNs and EPF to mitigate the SWD problem. Even though entomopathogenic nematodes and entomopathogenic fungi may not be able to provide 100% control of SWD populations, they may be a valuable addition to the IPM toolbox.

## The dual effects of winter cover crops on abundance of *Meloidogyne incognita* and biological control

Timper, Patricia

Retired, USDA ARS, Tifton, GA 31793

### Abstract

In the southeastern USA, cotton growers utilize conservation tillage (strip tillage) and winter cover crops to protect soil structure and function. These practices can have a direct effect on abundance of a primary pathogen of cotton, the root-knot nematode *Meloidogyne incognita*, and may also indirectly affect the nematode by altering the abundance of its antagonists. Winter cover crops can serve as hosts for *M. incognita*; many legumes are good hosts for this nematode, while many grasses are poor hosts. In a field study, we showed that planting cover crops that are good hosts leads to greater galling and lower yields in the subsequent cotton crop compared to planting poor hosts or fallow winters. We hypothesized that growing winter cover crops would enhance biological control of *M. incognita*. In a greenhouse study, cover crops were grown for 1 month before killing them with an herbicide. Cotton seed and the fungus *Purpureocilium lilacinum* was applied to a furrow and inoculated with the nematode. Compared to the fallow control, greater nematode suppression by the fungus was observed when residue from the cover crop was left on the soil surface; removing the residue resulted in similar or lower suppression compared to the fallow. In a field study, two field sites that had been planted with cover crops were sampled after cotton was planted to determine the abundance of predatory nematodes and the suppressiveness of the soil community. Two bioassays were conducted: one compared the survival of *M. incognita* second-stage juveniles (J2) in heated vs native soil and the other measured the reproductive potential of the nematode on cotton in a greenhouse. At the Jones Farm, fewer J2 survived in the native vs the heated soil (55% suppression), whereas at the Belflower Farm, fewer J2 survived in the native vs the heated soil in only 1 of 3 years. The level of suppression in J2 survival was similar among cover crops and fallow. The relative abundance of predatory nematodes was positively correlated (*P* = 0.007, *r* = 0.51) with suppression suggesting that the predators contributed to the reduced survival in the native soil. As with suppression of J2 survival, the abundance of predatory nematodes was similar among cover crops and fallow. Reproduction of *M. incognita* was greater (*P* = 0.04) in the fallow treatment than in the rye or rye/clover mix at the Belflower Farm; reproduction was similar among cover crop treatments at the Jones Farm. These studies indicate that cover crops can enhance biological control of root-knot nematodes; however, enhancement does not occur in all fields. The level and type of suppression of *M. incognita* appears to be field specific.

## A numbers game: comparing turfgrass nematode assay efficiencies from ten state labs

Tucker, M. Aaron, D. S. McCall and J. D. Eisenback

School of Plant and Environmental Sciences, Virginia Tech, Blacksburg, VA 24061

### Abstract

Different extraction methods are used by nematode assay labs for quantifying plant-parasitic nematodes (PPN) from turfgrass samples. The objective of this research was to compare the extraction efficiencies of homogenous turfgrass samples by various state nematode labs. Random samples were collected from three locations, homogenized, and divided into 30 subsamples from each of the three localities: 1) Virginia Tech Turfgrass Research Center (Blacksburg, VA), 2) Augustine Golf Club (Stafford, VA), and 3) Philadelphia Country Club (Gladwyne, PA). A subsample consisted of 500 ml of soil, and 10 state labs received 3 subsamples from each of the 3 locations (n = 90). Populations of PPN were extracted with either semi-automatic elutriation (n = 27) or hand sieving (n = 63) from the following state labs: Alabama (AL), Florida (FL), Louisiana (LA), Georgia (GA), Massachusetts (MA), Michigan (MI), Mississippi (MS), North Carolina (NC), South Carolina (SC), and Virginia (VA). Six PPN genera were identified that include *Hoplolaimus*, *Mesocriconema, Tylenchorynchus*, *Meloidogyne*, *Helicotylenchus*, and *Paratrichordorus*. An analysis of variance using JMP Pro 16^®^ (Cary, NC) (α = 0.05) determined differences in PPN populations among states with the same extraction methods. Using semi-automatic elutriation, the Virginia lab extracted more *Hoplolaimus* sp. than either LA or NC. All other genera were similar among these three state labs. Hand sieving was most variable, with four of the six genera resulting in differences in PPN populations counts among the remaining seven labs. This research stresses the importance of using the same lab for consistent nematode assays that are comparable from year to year.

## Abundance and distribution of plant parasitic nematodes in hop yards in Michigan

Usman, Muhammad^1^, E. Darling^1^, A. Palmisano^1^, L. Núñez-Rodríguez^2^, I. A. Zasada^3^, H. Chung^1^ and M. Quintanilla-Tornel^1^

^1^Department of Entomology, Michigan State University, East Lansing, MI 48823

^2^Department of Botany and Plant Pathology, Oregon State University, Corvallis, OR 97331

^3^USDA-ARS Horticultural Crops Disease and Pest Management Research Unit, Corvallis, OR 97330

### Abstract

Hop is an important crop in the United States. With a total cultivated area of 24,757 ha in 2022, the U.S. is the top producer of hop globally. Michigan ranks fourth and is the largest producer of hop outside the Pacific Northwest including Washington, Idaho, and Oregon. Plant-parasitic nematodes are considered as important limiting factors that are responsible for yield reduction in hop. To date, literature is lacking on the abundance and distribution of plant-parasitic nematodes in hop yards in the Great Lakes region. No surveys have been conducted in Michigan to assess the abundance of plant-parasitic nematodes in hop yards. The objective of this study was to determine the abundance and distribution of plant-parasitic nematodes in Michigan hop production. In this survey, 28 hop yards were sampled in 2021. Soil samples were collected from three hop varieties (Centennial, Cascade, and Chinook) and nematodes extracted from soil and identified and quantified. Root lesion nematodes (*Pratylenchus* spp.) were found to be the most widely distributed plant-parasitic nematodes. Of the total fields sampled, *Pratylenchus* spp. were detected in 96% of fields. The hop cyst nematode (*Heterodera humuli*) was detected in 50% of surveyed fields. This survey provides baseline information on the distribution and abundance of plant-parasitic nematodes in hop yards in Michigan. Increasing our knowledge on these pests will help growers mitigate the effect of plant-parasitic nematodes on hop production via effective management strategies.

## Quarantine actions and growing requirements to protect the Florida citrus industry from burrowing nematode and citrus greening has resulted in a reduction in nematode pests of citrus and citrus nurseries

Vau, Silvia and J. Stanley

Division of Plant Industry, 1911 SW 34th St, Gainesville, FL 32608

### Abstract

In the 1950s, a citrus disease called ‘Spreading Decline’, found to be caused by the burrowing nematode, *Radopholus similis*, devastated citrus orchards in Florida. The damage caused by this disease prompted the adoption of internal phytosanitary measures and the implementation of a Citrus Certification Program aimed at preventing the spread of major citrus nematode pests (Rule Chapter 5B-44.003, Florida Administrative Code). In addition to the burrowing nematode, this program included two additional citrus nematode pests, the citrus nematode, *Tylenchulus semipenetrans*, and coffee lesion nematode, *Pratylenchus coffeae*. The Citrus Certification Program is composed of three phases, (i) site approval, (ii) pit approval (peat, clay, sand, shell gravel used for citrus, and roads near citrus groves), and (iii) pre-movement certification of young citrus trees before they are moved from the nurseries and transplanted into groves. The three phases of the program not only requires that citrus propagative material be produced following strict sanitation practices, but also, that non-infested orchards be protected from the introduction of citrus nematodes from contaminated sources or infested orchards. In 2006, the Citrus Nursery Stock Certification Program (Rule 5B-62, Florida administrative Code) was amended to include more stringent growing conditions for Florida citrus nurseries. This rule was amended to combat the devastating effects of citrus greening, *Candidatus Liberibacter asiaticus*. These new phytosanitary requirements also led to a significant decrease in nematode contamination in Florida citrus production. Per year, around 350 samples are certified and presently, no Florida citrus nurseries are known to be infested by citrus nematode pests. In fact, most citrus nursery samples are free of plant-parasitic nematodes with the exception of site and pit samples. The common nematodes found with no regulatory significance are, *Criconemoides* sp., *Peltamigratus christiei*, *Pratylenchus brachyurus*, *Trichodorus minor*, and *Xiphinema americanum*.

## Novel cellular insights of beech leaf disease

Vieira, Paulo

USDA-ARS Mycology & Nematology Genetic Diversity & Biology Laboratory, Beltsville, MD

### Abstract

The beech leaf disease nematode, *Litylenchus crenatae mccannii,* is recognized as a newly emergent nematode species that causes beech leaf disease (BLD) in beech trees (*Fagus* spp.) in North America. Since the first report of BLD on *Fagus grandifolia* in Ohio in 2012, the disease has rapidly spread to eleven additional states and Canada. Changes of leaf morphology induced by BLD can provoke dramatic effects into the leaf architecture and consequently to tree performance and development. The initial symptoms of BLD appear as dark green interveinal banding patterns of the leaf. The cellular contribution to the formation of such aberrant leaf phenotype remains unknown. To understand the cellular basis of BLD, we employed different approaches of microscopy to provide an exhaustive characterization of nematode-infected buds and leaves. Nematodes can infect and use bud scales and leaves as sources of nutrients. Fresh and fixed histological sections revealed a dramatic cell change composition of these nematode-infected tissues. Diseased bud scale cells were typically hypertrophied and showed a high variability of size, probably related to their stage of development during nematode interaction. Hyperplasia and hypertrophy of the different leaf cell layers, particularly of the spongy mesophyll, resulted in the typical interveinal leaf banding. These discrepancies in leaf cell structure were depicted by an abnormal rate of cellular division of the leaf areas infected by the nematode, promoting a significant increase of the leaf thickness. The formation of symptomatic BLD leaves is therefore orchestrated by distinct cellular processes, involving an initial ectopic cell division of undifferentiated cells, followed by abnormal cell expansion. These results revealed a highly specialized mode of parasitism of *L. crenatae mccannii*.

## Response of the nematode community to reduced-risk nematicides in low desert vegetable cropping systems

Waisen, Philip

Division of Agriculture and Natural Resources, University of California Cooperative Extension, Palm Desert, CA 92211

### Abstract

Considering the current global paradigm shift in favoring the use of environmentally conscious approaches, high-risk nematicides are either banned or their use is being restricted. Reduced-risk nematicides like fluazaindolizine (Salibro^®^) and fluopyram (Velum^®^ One) offer selective modes-of-action and are utilized as alternative to high-risk nematicides. However, limited information is available on their effects on soil health. Free-living nematodes are considered good soil health bioindicators. The objective of this study was to examine the target and non-target effects of Salibro and Velum on the nematode community. A field trial was conducted during the summer of 2022 in Coachella Valley, Riverside, California. Treatments included Salibro at 2.26 L/ha applied 2 weeks after planting, at 1.13 L/ha 2- and 4-weeks after planting; Velum at 0.50 L/ha 4- and 6-weeks after planting; and, an untreated control. The nematicide treatments were delivered through chemigation. Sixteen treatment plots each measuring 113 m × 1 m were directly seeded with okra (*Abelmoschus esculentus*). Each treatment was replicated 4 times and arranged in a randomized complete block design. Twelve composite soil samples were collected from the top 20 cm before chemigation and at monthly intervals thereafter for the duration of the okra crop. An aliquot of 100 cm^3^ soil was subjected to Baermann tray method for nematode extraction. *Meloidogyne* spp., bacterivores, fungivores, and omnivores were morphologically identified. Salibro suppressed *Meloidogyne* spp. without impacting the abundance of free-living nematode of all trophic groups. However, Velum suppressed both *Meloidogyne* spp. and free-living nematodes alike. Among the two reduced-risk nematicides tested, Salibro appeared to impose no threat to soil health.

## Long-term fertilization and cultivation impacts on nematode abundance and community structure in tall fescue turfgrass

Waldo, Benjamin^1^, F. Shahoveisi^2^ and M. Carroll^2^

^1^USDA-ARS, Mycology and Nematology Genetic Diversity and Biology Laboratory, Beltsville, MD 20740

^2^Department of Plant Sciences and Landscape Architecture, University of Maryland, College Park, MD 20742

### Abstract

Impacts of long-term fertilization and cultivation were evaluated on nematode communities associated with tall fescue turfgrass following 11 years of treatment applications. Fertilizer treatments of biosolid, synthetic, and plant-based fertilizers and cultivation treatments of 0x, 1x, and 2x aerification passes were applied to tall fescue plots. The experimental plots were arranged as a randomized complete block design with three replicates at the University of Maryland Paint Branch Turfgrass facility in College Park, Maryland. Free-living and plant-parasitic nematodes were identified, enumerated, and categorized into functional groups. Nematode count data were compared using generalized linear mixed modeling with negative binomial distribution and two-way ANOVA was used to compare nematode ecological indices. Biosolid fertilizer treated plots had lower omnivore-predator densities than plant-based fertilizer treated plots (*P* ≤ 0.001) and significantly greater *Hoplolaimus* densities than plant-based fertilizer plots (*P* ≤ 0.05). Synthetic fertilizer plots had the greatest bacterivore densities (*P* ≤ 0.001) of all fertilizer treated plots. Plots with 1x cultivation had fewer total bacterivores than 2x cultivated plots (*P* ≤ 0.01). These findings suggest that long-term turfgrass management practices can have variable impacts on nematode community abundance in tall fescue and may be informative for turfgrass managers depending on the nematodes present in their field.

## Nematode linkage to regenerative agriculture in the tropics/subtropics

Wang, Koon-Hui^1^, R. Paudel^1^, J. Marquez^1,2^ and P. Waisen^3^

^1^Department of Plant and Environmental Protection Sciences, University of Hawaii, Honolulu, HI 96822

^2^Hawaii Department of Agriculture, Honolulu, HI 96814

^3^Division of Agriculture and Natural Resources, University of California Cooperative Extension, Riverside, CA 92211

### Abstract

Soil degradation is happening faster in the tropics/subtropics than in temperate environments, making this region more vulnerable to climate change. Regenerative agriculture emphasizes reducing soil disturbance, maintaining soil cover, constantly growing living roots, while synergizing biodiversity through crop rotations to support ecosystem services for sustainable food production. Here we review the efficacies of cover cropping, conservation tillage, and crop rotation in improving soil food web structure while suppressing plant-parasitic nematodes (PPN) in the tropics/subtropics in an attempt to better prescribe soil health practices. Brown mustard is known for allelopathy against PPN through biofumigation when the biomass is macerated and tilled into the soil. This brown mustard termination method is also capable of enriching the soil by increasing bacteria-feeding nematodes and enrichment index (EI), but consistently reducing channel index (CI), and could not improve soil food web structure indicated by the structure index (SI) in the subsequent cash crops. Conversely, sunn hemp produces monocrotaline, when terminated by strip tilling and periodic clipping as surface organic mulch, suppressed PPN while increasing bacterial, fungal and omnivorous nematodes within the first cropping cycle, followed by an increase in SI in the second repeated cycle, hence improving soil food web structure of a cucumber crop. However, both brown mustard and sunn hemp are slow in adding soil organic matter (SOM). Despite lacking a nematode allelopathic effect, black oat, was able to increase SOM by 0.4% in the subsequent corn crop after 2 years of no-till farming but failed to improve water infiltration in a high clay Oxisol in Hawaii following a history of 9 years of no-till farming. Sorghum/sorghum-sudangrass hybrids (SSgH) offer another biofumigant, hydrogen cyanide, which upon hydrolysis, are effective against PPN if thoroughly soil incorporated. Under low PPN pressure, strip- and low-till SSgH improved water infiltration (I) and soil carbon (C) at cover crop termination compared to bare ground (BG) in Oxisol and led to lower root galls on eggplant but failed to maintain higher SOM on the subsequent eggplant crop. Strip-till of velvet bean outperformed SSgH for soil health management in a sweet potato agroecosystem. Seven multivariate analyses of various soil health parameters indicated an association of SI to increase in I, VSM, and SOM. However, when PPN pressure was high, SI was positively related to root-gall index but not I, VSM, and SOM. Instead, the biomass of arbuscular mycorrhizal fungi became the driving factor to increase I, VSM, SOM and soil aggregate stability. Clearly more work is needed to better prescribe regenerative agricultural practices for the tropics/subtropics that can improve resiliency in these vulnerable yet potentially productive agroecosystems. Keeping PPN pressure in check plays a role in facilitating the ecosystem services of cover crops, conservation tillage, and crop rotation.

## Entomopathogenic nematodes as biocontrol for Flatheaded Appletree Borer (Chrysobothris femorata)

Wong, Colin and D. Shapiro

USDA-ARS, SE Fruit and Tree Nut Research Laboratory, Byron, GA 31008

### Abstract

This study looked at the entomopathogenic nematodes (EPN) *Steinernema spp.* and *Heterorhabditis spp.* as biocontrol for *Chrysobothris femorata (*Flatheaded Appletree Borer), a pest of orchard and ornamental trees. The insect larvae cause damage which can severely impact the productivity or marketability of young trees, and can exacerbate other causes of poor tree health. *C. femorata,* like other wood boring pests can be difficult to manage due to their cryptic life style of living as larvae under the bark of trees. Many traditional chemical insecticides cannot reach insects under the bark of a tree. Some forms of biocontrol can target insects in these cryptic habitats due to their ability to move through the environment after application. *In vitro* laboratory assays using five strains of EPN have shown that *C. femorata* larvae are susceptible. Two strains, *Steinernema carpocapsae* (All), and *Steinernema riobrave* (355) were chosen to take to field trials. Field tests were carried out in two locations by spraying young trees in areas with previous damage from Flatheaded borer. Subsequent checks for damage found mixed results from the nematode treatments. The tests in a walnut orchard in California found that there was significantly less damage in one nematode treatment compared to the control, but a test in maple in Tennessee found no significant differences between treatments. These tests will be replicated in upcoming years.

## Mortality of the sweet potato weevil (*Cylas formicarius*) larvae caused by *Steinernema feltiae*

Wong, L., K. -H. Wang and B. S. Sipes

Department of Plant and Environmental Protection Sciences, University of Hawaii, Honolulu, HI, 96822

### Abstract

The sweet potato weevil (SPW), *Cylas formicarius*, is a cosmopolitan pest important to all sweet potato growing regions. SPW tunnel through the sweet potato leaving behind fecal pellets which ruins the marketability of the sweet potato. The entomopathogenic nematode *Steinernema feltiae* MG-14 is effective in the laboratory at a dose equivalent to 2.5 billion infective juveniles (IJ)/ha. SPW larval mortality was compared at various doses of *S. feltiae* MG-14. For each dose, five SPW larvae were placed in an inoculation court consisting of a 50 × 20 mm petri dish lined with Whatman filter paper #1. Each inoculation court was infested with 0, 105 IJ which was equal to 0.5 billion, 210 IJ (1 billion) or 525 IJ (2.5 billion IJ/ha). SPW larvae were exposed for 48 hrs with mortality evaluated every 24 hrs. Cadavers were placed on individual White traps and IJ emergence was determined after 21 days. The experiment was repeated three times. In the first two trials 3 replicates were used and the third trial 5 replicates were used. In the 0 IJ dose, mortality was 31.7%. At 0.5 billion IJ/ha, mortality was 78.3%. At 1 billion IJ/ha, mortality was 71.7%, and at 2.5 billion IJ/ha, mortality was 81.7%. The treatments were not different from each other, but all were different compared to the uninoculated control (*P* < 0.01). Emergence of IJ was observed from 56.9% inoculated cadavers. Lack of IJ emergence may have been due to incidental death of the SPW larva from handling or starvation. Future research will evaluate the performance of *S. feltiae* MG-14 in the field at these various doses.

## Automated counting of root-knot nematode eggs using PlantCV

Wram, Catherine^1^, T. Wiesner-Hanks^2^, X. Tang^2^, M. Lin^2^, B. Waldo^3^, P. A. Wadl^1^, T. Slonecki^2^, C. Beil^2^, M. Sheehan^2^ and W. Rutter^1^

^1^USDA-ARS US Vegetable Laboratory, Charleston, SC 29414

^2^Breeding Insight, Cornell University, Ithaca, NY 14853

^3^USDA-ARS Mycology & Nematology Genetic Diversity & Biology Laboratory, Beltsville, MD 20705

### Abstract

Estimating nematode reproduction is an important step in a wide range of research applications, including breeding for host resistance, evaluating nematicides, and evaluating the efficacy of management practices. In root-knot nematodes (RKN), *Meloidogyne* spp., this is traditionally done by extracting eggs from an infested root system using NaOCl, diluting the extracted sample, and enumerating by hand under a microscope. Counting eggs is time intensive, laborious, and requires special training to properly identify eggs. In this study, we developed and tested an automated counting pipeline to enumerate RKN eggs from microscope images taken in a 96-well plate format. We extracted *Meloidogyne incognita* and *Meloidogyne enterolobii* eggs using the NaOCl method from 51 sweetpotato accessions, including both known resistant and susceptible genotypes. For the traditional method, 1 mL aliquots from each egg extraction were diluted 1:16 or 1:64 and 3 counts were made of the dilutions under a dissecting scope and averaged to determine total eggs per a sample. To generate images for the automated egg counting algorithm, 4 independent aliquots were taken from each sample and serially diluted on a 96-well optical-bottom plate, with 6 egg samples represented on each plate. Each plate was imaged using a Keyence automated inverted microscope. Images were captured from the center of each well with four images collected per a dilution level (1:1,1:2,1:4,1:8,1:16, or 1:64) for each egg sample. Egg identification and counting was done using the image analysis software PlantCV, with filtering added to remove small objects that were not eggs, split up clumps of eggs, and remove objects that were not compacted and were not similar to the hand curated egg shape template. Overall higher dilution levels performed best, with the correlation coefficient determined by a Poisson regression between mean machine count and human count of 0.74 at the 1:1 dilution level. The correlation coefficient reduced to 0.38 at the 1:64 dilution level. Compared to traditional egg quantification methods, this pipeline provides a fast and efficient way to replicate egg counting for RKN that will speed up research efforts to combat RKN.

## Persistence of entomopathogenic nematodes in a novel capsule formulation

Wu, Shaohui^1^, M. D. Toews^2^, S. Arthurs^3^ and D. I. Shapiro-Ilan^4^

^1^Department of Entomology, Ohio State University, Columbus, OH 43210

^2^Department of Entomology, University of Georgia, Tifton, GA 31793

^3^BioBee USA, Tucker, GA 30084

^4^USDA-ARS, SE Fruit and Tree Nut Research Laboratory, Byron, GA 31008

### Abstract

Entomopathogenic nematodes (EPNs) naturally infect insect pests and can be used as biopesticides in pest management programs. However, the field persistence and efficacy of EPNs is highly dependent on environmental conditions. Here, we compared the persistence of the EPN *Steinernema feltiae* ENO2 strain in a novel capsule formulation versus the traditional aqueous application in laboratory and field tests by baiting with *Tenebrio molitor* larvae at various times after application. In the laboratory, soil-cup tests were used to compare the capsule formulation with the aqueous application of the ENO2 strain plus *S. feltiae* SN strain at 23°C at 0, 7, 14, 21 and 28 days after treatment (DAT). The capsule formulation had the longest persistence, followed by SN aqueous application, while the aqueous ENO2 had the shortest persistence. In particular, compared with 0 DAT, a significant decline in dead *T. molitor* was observed starting at 28, 14 and 14 DAT for ENO2 capsule, ENO2 aqueous and SN aqueous treatment, respectively. ENO2 capsule had significantly more dead *T. molitor* than ENO2 aqueous at 7–21 DAT but was not different from SN aqueous application. The field trials were conducted in a pecan orchard (located in Byron, GA) in 2021–2023 by applying ENO2 capsule and aqueous treatments in a randomized complete block design and baiting with *T. molitor* at 0, 3, 7, 14 and 21 DAT. Consistent with laboratory tests, ENO2 capsule showed enhanced persistence over the ENO2 aqueous application in the field. In fall 2021 and spring 2022, ENO2 capsule had peak activity at 3 DAT and declined persistence at 14–21 DAT; ENO2 aqueous treatment had short persistence and was not statistically different from the control starting at 3–7 DAT. Additional field trials in tilled soil in the pecan orchard are being evaluated in spring 2023. Thus far, our findings show that the capsule formulation may enhance EPN persistence and thereby potentially leverage the control efficacy against arthropod pests in commercial use.

## An investigation of management strategies to mitigate orchard replant disorder in cherry orchards

Yaghoubi, Ali, R. Yazdani and M. Quintanilla

Department of Entomology, Michigan State University, East Lansing, MI, 48823

### Abstract

Replant disorders can significantly reduce cherry orchard productivity. Some plant-parasitic nematodes are associated with replanting problems. In this study, we evaluated the effects of various chemical and organic amendments on plant-parasitic and beneficial nematode populations and cherry tree growth and development in two cherry cultivars, Emperor Francis and Ulster. A randomized block design with four replications was used to conduct the experiment in May 2022. The treatments were Seed Starter Compost + Dairy Doo + Straw bales, Dairy Doo, Seed Starter Compost, Velum, fumigation with Telone C-35, and untreated control. The nematodes present at the beginning of the trial were *Pratylenchus penetrans*, *Xiphinema americanum*, *Paratylenchus* spp., *Mesocriconema xenoplax*, and beneficial nematodes. Tree growth measurements and soil samples were taken in May and September 2022. The results showed that fumigation significantly increased tree trunk cross-section area, limb length, and canopy height in Ulster trees. In the Emperor Francis trees, limb length and canopy height increased compared to the untreated control, but not significantly, however, there was a significant increase in limb length and canopy height compared to Dairy Doo. Soil analysis revealed that the combined use of Seed Starter Compost + Dairy Doo + Straw bales significantly increased beneficial nematodes in the Ulster trees compared to the untreated control. The Velum treatment reduced densities of root lesion and beneficial nematodes during the season, but in the fumigation treatment, root lesion nematodes reproduced faster than the beneficial nematodes and had higher population levels than the initial soil sampling. The results showed that the combination use of Seed Starter Compost + Dairy Doo + Straw bales prevented the reproduction of root lesion nematodes and increased the reproduction rate of beneficial nematodes. We plan to evaluate plant yield and growth further by analyzing the bloom of each tree, measuring the length and width of 1-year-old non-fruit spurs, and determining the yield and quality parameters of the fruit in the next year.

## First draft genome assembly of the root-lesion nematode *Pratylenchus scribneri* generated using long-read sequencing

Yan, Guiping and D. Arora

North Dakota State University, Department of Plant Pathology, Fargo, ND 58108

### Abstract

The root-lesion nematode *Pratylenchus scribneri* is a biotrophic parasite of agriculturally important crops, including but not limited to potato, corn, and soybean. Infection by root-lesion nematodes can result in lesions on plant roots reducing the amount of root branching and the ability of the roots to absorb water and nutrients from soil, causing economic losses. Despite being an economically important plant-parasitic nematode group of more than 100 species, genome information related for *Pratylenchus* spp. is scarcely available. The objective of this research was to generate a contiguous and relatively complete annotated genome of the important nematode *P. scribneri*. In this study, two DNA extraction protocols for library preparation and sequencing were compared; one using 8,000 to 10,000 mixed-stage individuals of *P. scribneri* from the nematode suspension derived from carrot cultures, and another using 500 *P. scribneri* adults and juveniles handpicked from the original nematode suspension. The library prepared using DNA extracted from 500 handpicked nematodes was found much cleaner (fewer contaminants) as compared to the library prepared from DNA extracted directly from the suspension with a higher number of nematodes from the carrot cultures. Therefore, the *de novo* draft genome assembly of *P. scribneri* was subsequently generated on the PacBio Sequel II System using the ultra-low DNA input HiFi sequencing workflow with significantly fewer nematodes. The final assembly created using 5 ng of input DNA from 500 nematodes consisted of 276 decontaminated contigs, with an average contig N_50_ of 1.72 Mb, GC content of 32.8% and an assembled draft genome size of 227.24 Mb consisting of 51,146 predicted protein sequences. The Benchmarking universal single-copy ortholog (BUSCO) analysis indicated that the final decontaminated assembly had a completeness score of 65.4% with 24.0% being single copy, 41.4% duplicated, 1.8% fragmented, and 32.8% missing BUSCOs out of the total 3,131 orthologs used. The outputs from GenomeScope2 and Smudgeplots converged towards a diploid genome for *P. scribneri*. This is the first time that a nematode genome assembly using such low number of nematodes is being reported. The new workflow described here will facilitate the genome sequencing of new and existing species of plant-parasitic nematodes that cannot be mass-produced. The data provided here will facilitate future studies on evolutionary and lifestyle mechanisms of root-lesion nematodes, host plant-nematode interactions, and crop protection.

## The potential of applying plasma-water treatment in *Meloidogyne enterolobii* management

Yang, Jiue-in, S-c. Huang and J-r. Lai

National Taiwan University. Department of Plant Pathology and Microbiology, Taipei City 106216, Taiwan

### Abstract

The guava root-knot nematode, *Meloidogyne enterolobii,* is an aggressive plant-parasitic nematode with high pathogenicity. It can overcome known resistance genes in plants and has caused enormous economic losses on crops such as tomato and guava. Plasma-activated water (PAW) is a new eco-friendly material generated by treating water with cold atmospheric plasma. Various studies have shown the antimicrobial activity of PAW on bacteria and fungi. Therefore, this study aimed to investigate the physiological influences, the lethal effect, and the control efficacy in pot applications with *M. enterolobii*, hoping to provide a solid foundation for PAW application on plant nematode disease management. As a result, observations revealed that PAW inhibited the embryogenesis and hatching of *M. enterolobii* eggs. Of the PAW-treated eggs, 74% stopped development before reaching the first-stage juveniles, and the rest did not develop beyond that stage. PAW delayed the hatching of matured eggs, and the hatching rate decreased by 70%. When hatched second-stage juveniles (J2) were treated with PAW for 24 hours, 83% died. The total amount of reactive oxygen species in the PAW-treated J2 increased by 35%, indicating that PAW caused oxidative stress on J2 in a short period and influenced their survival. In a plate-inoculation experiment, the number of *M. enterolobii* J2 successfully invading tomato roots decreased by 65.3% in the PAW environment. In tomato pot experiments, when plants were irrigated with PAW, *M. enterolobii* J2 development slowed down. At 35 days post-inoculation, only 25% became adults, and the number of galls decreased by 40%. At the same time, PAW changed the nematode population structure and size; the male ratio increased, and the number of offspring decreased. Meanwhile, PAW reduced nematode disease symptoms and promoted tomato growth. Similar nematode control and plant growth-promoting efficacy were observed in another Chinese cabbage greenhouse trail. In summary, this is the first study to demonstrate that PAW caused oxidative stress to *M. enterolobii* and adversely affected every development stage of the *M. enterolobii* life cycle while promoting plant growth.

## Compost application as a sustainable approach to managing soybean cyst nematodes in corn-soybean rotations

Yazdani, Razieh^1^, S. Thapa^2^, A. Yaghoubi^1^, A. D. Howland^1^ and M. Quintanilla^1^

^1^Department of Entomology, Michigan State University, East Lansing, MI, 48823

^2^Corteva Agriscience Company

### Abstract

Soybean cyst nematode (SCN) continues to be a major yield-limiting factor for soybean growers, causing significant economic losses in the United States. In Michigan alone, SCN is responsible for an estimated $40 million in losses annually. Through the continual use of resistant cultivars and chemical treatments, the nematode has developed resistance to these methods, necessitating the exploration of alternative management techniques. Composting is an emerging strategy that offers a sustainable and effective solution for controlling SCN populations. To investigate the impact of different compost types - poultry manure, layer ash blend, and swine manure - on SCN, a randomized complete block trial with four replicates was conducted under field conditions in 2021–2022. Composts were applied at a rate of 1.25 tons per acre prior to corn planting, and soil samples were collected at various stages to assess the effect of composts on SCN populations. The study found that the use of layer ash blend and swine manure resulted in a lower number of SCN eggs (1,407 eggs/100 cm^3^ soil and 1,641.4 eggs/100 cm^3^ soil, respectively) compared to the untreated control, which had 2,885.8 eggs/100 cm^3^ soil at harvest time. Additionally, soybean yield increased by 3.24% and 6.48% with the use of layer ash blend and swine manure, respectively, compared to the untreated control. The untreated control also exhibited a significant increase in the reproduction factor of nematode eggs, with a ratio of 2.19 compared to 1.45, 1.58, and 1.31 for the layer ash blend, poultry manure, and swine manure treatments, respectively. These findings demonstrate the potential of composting, especially using layer ash blend and swine manure, as a sustainable and effective strategy for managing SCN populations and offering benefits to soybean growers by increasing yield and reducing nematode population and economic losses.

## An update of regulated and recent discoveries of invasive alien plant-parasitic nematodes species in Canada

Yu, Qing^1^ and F. Sun^2^

^1^Ottawa Research and Development Center, Agriculture and Agri-Food Canada, Ottawa, ON K1A 0C6, Canada

^2^Canadian Food Inspection Agency, 3851 Fallowfield Road, Ottawa, ON, K2H 8P9, Canada

### Abstract

With the most recent discovery of the invasive nematode species *Litylenchus crenatae* ssp. *mccannii* in Ontario, Canada, the etiological agent causing beech leaf disease, and the 2006 report of *Globodera rostochiensis* from potato in the Saint-Amable region, Quebec, it is time to provide updated information on the Canada-regulated nematode pests, and recent discoveries of invasive alien nematode species in Canada. Since 2012, the stem and bulb nematode *Ditylenchus dipsaci* has caused epidemic disease on garlic in Ontario and it was subsequently found in neighboring provinces. Around that time, *Ditylenchus destructor* was also discovered on garlic in Ontario. *Ditylenchus weischeri* was identified in yellow peas in the Prairie provinces not *Ditylenchus dipsaci* as first thought. In 2013, the soybean cyst nematode *Heterodera glycines* was deregulated. Other recently discovered invasive alien plant parasitic nematode species are: *Heterodera carotae, Meloidogyne naasi, Pratylenchus alleni, Pratylenchus thornei, Trichodorus primitivus, Xiphinema chambersi*. The current updated list of regulated nematodes in Canada includes: *D. dipsaci, D. destructor, Globodera pallida, G. rostochiensis, Longidorus* spp. (virus vector), *Meloidogyne chitwoodi, Trichodorus spp.* (virus vector), and *Xiphinema* spp. (virus vector).

